# Circulating neutrophil anti-pathogen dysfunction in cirrhosis

**DOI:** 10.1016/j.jhepr.2023.100871

**Published:** 2023-08-01

**Authors:** Irina Balazs, Vanessa Stadlbauer

**Affiliations:** 1 Department of Internal Medicine, Division of Gastroenterology and Hepatology, Medical University of Graz, Graz, Austria; 2 Center for Biomarker Research in Medicine (CBmed), Graz, Austria

**Keywords:** neutrophils, cirrhosis, phagocytosis, ROS, chemotaxis, NETs

## Abstract

Neutrophils are the largest population of leucocytes and are among the first cells of the innate immune system to fight against intruding pathogens. In patients with cirrhosis, neutrophils exhibit altered functionality, including changes in phagocytic ability, bacterial killing, chemotaxis, degranulation, reactive oxygen species production and NET (neutrophil extracellular trap) formation. This results in their inability to mount an adequate antibacterial response and protect the individual from infection. Prognosis and survival in patients with cirrhosis are greatly influenced by the development of infectious complications. Multidrug-resistant bacterial infections in patients with cirrhosis are currently a growing problem worldwide; therefore, alternative methods for the prevention and treatment of bacterial infections in cirrhosis are urgently needed. The prevention and treatment of neutrophil dysfunction could be a potential way to protect patients from bacterial infections. However, the reasons for changes in neutrophil function in cirrhosis are still not completely understood, which limits the development of efficient therapeutic strategies. Both cellular and serum factors have been proposed to contribute to the functional impairment of neutrophils. Herein, we review the current knowledge on features and proposed causes of neutrophil dysfunction in cirrhosis, with a focus on current knowledge gaps and limitations, as well as opportunities for future investigations in this field.

**Key points**

• Neutrophil dysfunction in cirrhosis is associated with the development of bacterial infections and higher mortality.
• The functions of neutrophils that are impaired in cirrhosis include phagocytosis, ROS production, killing capacity, neutrophil extracellular trap formation and chemotaxis.
• Further features of neutrophil dysfunction in cirrhosis are still to be described and standardisation of methodology is needed.
• Intrinsic and serum defects have been proposed to cause neutrophil dysfunction, although neither can fully describe the extent of neutrophil dysfunction and findings are contradictory.
• Due to the lack of clinical studies with neutrophil function as a primary or secondary outcome, there is only a vague understanding of the best strategy to prevent and treat neutrophil dysfunction in cirrhosis.
• Further research is needed to develop a neutrophil function screening panel and biomarkers for clinical practice, and to further describe neutrophil dysfunction, its causes and consequences, and approaches for its prevention and treatment.

## Introduction

Cirrhosis is a life-threatening chronic and progressive liver disease that develops primarily as a consequence of chronic hepatitis B or hepatitis C virus infections, or alcohol-related or non-alcoholic steatohepatitis, with other rarer causes including alpha-1-antitrypsin deficiency, haemochromatosis, primary sclerosis cholangitis, Wilson’s disease, etc.^1^^,^^2^ Being the 11th leading cause of death in the world, cirrhosis accounted for 2.2% of total deaths in 2016^3^ and 2.4% of total deaths in 2017,^4^ with an annually increasing mortality rate.^4^^,^^5^ Compensated patients with cirrhosis have an ∼5-fold increased mortality risk and decompensated patients a 10-fold increased mortality risk compared to the general population.^6^ The prognosis and survival of patients with cirrhosis are greatly influenced by the development of bacterial infections, such as spontaneous bacterial peritonitis, urinary tract infections, and pneumonia, among others.[7], [8], [9]

Around one-third of hospitalized patients with cirrhosis suffer from bacterial infections, which leads to increased hospitalization time,^7^ de-listing from the transplantation waiting list,^10^ acute kidney injury^11^ and, hence, a 4-fold increased mortality rate compared to non-infected patients with cirrhosis.^8^ Of those with cirrhosis who develop bacterial infections, 30% die within 1 month and more than 60% die within 1 year after the infection.^8^

Infections in patients with cirrhosis are mostly caused by gram-negative bacteria (such as *Escherichia coli* and *Klebsiella pneumoniae*) of intestinal origin, but infections caused by gram-positive bacteria (such as *Staphylococcus aureus* and Enterococci) are on the rise, particularly in hospitalized patients.^12^ Multidrug-resistant bacterial infections in cirrhosis are currently a growing problem worldwide; therefore, alternative methods for the prevention and treatment of bacterial infections in cirrhosis are urgently needed.^13^^,^^14^

An important reason for the increased susceptibility to bacterial infections in patients with cirrhosis is the progressive development of immune dysfunction.^15^ Innate immune dysfunction, including impaired neutrophil functionality, is a predominant part of cirrhosis-associated immune dysfunction and, therefore, plays a major role in the development of bacterial infections in patients with cirrhosis.^16^ The number of circulating neutrophils is frequently altered in cirrhosis. Neutropenia is often described in cirrhosis,^17^ whereas neutrophilia has been reported for acute-on-chronic liver failure (ACLF).^18^^,^^19^ The neutrophil-to-lymphocyte ratio is associated with liver-related mortality in patients with different stages of cirrhosis,^17^^,^^20^ as well as in patients with ACLF.^19^^,^^21^ Furthermore, an increased neutrophil-to-lymphocyte ratio in patients with cirrhosis positively correlates with the number of circulating low-density neutrophils, which are considered pro-inflammatory.^20^ The role of the neutrophil-to-lymphocyte ratio in cirrhosis has been extensively described elsewhere.^22^ However, the number of neutrophils alone is not sufficient to understand the complex functional defects that are present in cirrhosis.

There is growing evidence of various defects in neutrophil function occurring during the course of cirrhosis; additionally, these defects have been associated with poor prognosis and mortality. Recent reviews by Irvine *et al.* 2019,^12^ Bernsmeier *et al.* 2020,^16^ and Albillos *et al.* 2022,^23^ which address the problem of cirrhosis-associated immune dysfunction, provide only a succinct overview of changes in neutrophil function and their causes in cirrhosis, as they review the functional changes of other immune cells as well. Recent neutrophil-focused reviews by Xu *et al.* 2014,^24^ Cho *et al.* 2020^25^ and Liu *et al.* 2021^26^ also provide only a relatively short overview of neutrophil dysfunction in cirrhosis, as they aim to provide an overview of neutrophil dysfunction in various liver diseases not limited to cirrhosis.

We therefore provide a detailed overview of various changes in neutrophil function specifically in cirrhosis, address the problem of inter-study discrepancies and point out differences in the methodology used to measure neutrophil function. Furthermore, we discuss the current theories explaining the nature of cirrhosis-associated neutrophil dysfunction, their pros and cons, and potential therapeutic targets and directions for future studies. We think it is time to critically review the knowledge gathered on the topic, identify and discuss current gaps, problems and conflicting findings, and thereby create a basis for future investigations.

## Features of neutrophil dysfunction in cirrhosis

Neutrophils are the largest population of leucocytes and are among the first cells of the innate immune system to fight against intruding pathogens. Neutrophils express a range of surface G protein-coupled receptors, which enable them to sense chemoattractants (inflammatory mediators or pathogen-related molecules) and, consequently, migrate to the site of the intruding pathogen (mostly bacteria and fungi). This process of directed migration is called “chemotaxis”. As soon as neutrophils reach the site of infection, they use one of their defence mechanisms to protect the host organism from the infectious agent. They can internalise the pathogen via a process called “phagocytosis” and kill it inside the phagolysosome using the microbiocidal contents of their intracellular granules (*e.g*. various proteases) and reactive oxygen species (ROS). These substances can also be released into the extracellular space by neutrophils to enable extracellular bacterial killing, but this can also cause tissue damage. Neutrophils produce different anti- and pro-inflammatory cytokines, which help regulate inflammatory as well as other physiological and pathophysiological processes. Furthermore, neutrophils have recently been described as being able to extrude their DNA, which is coated with histones and cytoplasmic and granular proteins (*e.g.* neutrophil elastase, myeloperoxidase, *etc*.), *i.e*. the so-called “neutrophil extracellular traps” (NETs), in order to catch, immobilize and kill infectious agents. Neutrophils are cells with a relatively short lifespan, which is extended in the case of infection. Neutrophils undergo apoptosis and then are phagocytosed by macrophages and dendritic cells to promote the resolution of inflammation (reviewed in [27], [28], [29]).

**Fig. 1:**
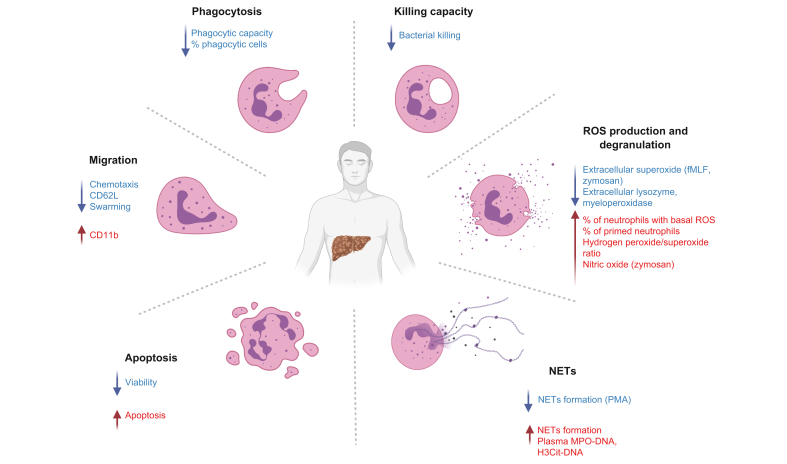
Summary of neutrophil dysfunction features in cirrhosis. Findings consistent throughout the literature are shown. Created with Biorender.com.

All the aforementioned functions allow neutrophils to fight bacterial infections. In patients with cirrhosis, neutrophils exhibit altered functionality, including changes in phagocytic ability, bacterial killing, chemotaxis, degranulation, reactive oxygen species production and NET formation (Fig. 1, Table S1). This results in their inability to mount an adequate antibacterial response and protect the patient from infection.

### Chemotaxis

Neutrophil chemotaxis is reduced in patients with cirrhosis, either because of cellular defects or the impaired chemoattractant activity of cirrhotic serum.[30], [31], [32], [33], [34], [35] Neutrophils from patients with alcohol-associated cirrhosis show reduced migration towards healthy serum compared to healthy donor neutrophils. Serum from patients with cirrhosis had less chemoattractant activity compared to healthy serum in the same study. No correlation with bacterial infections has been found;^30^ however, another study reported lower neutrophil migration ability in patients with cirrhosis with previous bacterial infections compared to those without previous infection.^34^ The decrease in serum chemoattractant activity might be explained by alterations in chemoattractant production found in patients with cirrhosis.^36^ Interestingly, another study shows that serum chemotactic inhibitory activity (which shows how effectively cirrhotic serum inhibits the chemoattractant activity of healthy serum mixed with zymosan A) is higher in alcohol-associated cirrhosis compared to non-alcoholic cirrhosis.^32^ Alcohol intake *per se* is a likely contributor to neutrophil dysfunction in alcohol-related cirrhosis as it has previously been shown to reduce neutrophil chemotaxis in rats,^37^ transiently reduce neutrophil ROS production and phagocytosis in healthy volunteers,^38^ and cause dysregulation of neutrophil function upon *in vitro* alcohol exposure.^39^ Other studies also report the presence of serum chemotactic inhibitory activity in patients with cirrhosis.^33^^,^^35^ Cirrhotic neutrophils show decreased transendothelial migration in response to N-formyl-met-leu-phe (fMLF), increased adhesion to endothelial cells (HMEC-1) and an altered expression of adhesion receptors, and higher CD11b and lower CD62L expression, which also indicates increased degranulation^40^ and neutrophil ageing,^41^ compared to healthy controls.^31^ Decreased neutrophil migration towards interleukin (IL)-8 has been shown in cirrhosis, probably due to the decreased expression of CXC motif chemokine receptor 2 (which senses IL-8).^42^ Also, decreased migration and adhesion of neutrophils isolated from patients with cirrhosis in response to leukotriene B4 has been reported.^36^ Interestingly, the impairment in migration in the presence of fMLF is more pronounced in patients with acute decompensation and ACLF than in patients with compensated cirrhosis or healthy volunteers, which is less evident or absent (though with a tendency towards a decrease) when CXC motif ligand (CXCL)8 or CXCL1 is used as a chemoattractant. Furthermore, neutrophil migration patterns correlate with the incidence of adverse outcomes in this cohort of patients.^43^ Both CXCL8 (IL-8) and CXCL1 have been shown to be highly elevated in the serum of patients with cirrhosis, which is even more pronounced in patients with ACLF.[44], [45], [46] This might initially preserve chemotaxis due to ligand excess or may also cause the homologous desensitisation of their receptors on neutrophils due to the prolonged excess of ligands, their downregulation and the subsequent decrease in neutrophil migration. The higher grade of impairment of neutrophil migration towards fMLF might also be explained by the different mechanisms involved, *e.g*. another FPR1 ligand that is highly abundant in cirrhosis, such as chenodeoxycholic acid (CDCA), might compete with fMLF for binding to the receptor and, as a result, inhibit neutrophil chemotaxis.^47^ When casein is used as a chemoattractant, neutrophil chemotaxis is not affected in patients with cirrhosis. This indicates that defects in chemotaxis in cirrhosis are limited to certain pathways and depend on the chemoattractant used. The same study shows that the random migration of neutrophils is not affected in cirrhosis.^48^

### Swarming

Only one study so far implies that swarming (coordinated neutrophil communication and recruitment) of neutrophils in order to control *Candida albicans* hyphae growth is impaired in patients with cirrhosis compared to healthy controls.^49^

### Phagocytosis

Multiple studies have reported on the impaired phagocytic function of neutrophils from patients with cirrhosis.^34^^,^[50], [51], [52], [53], [54], [55], [56], [57], [58] Defective phagocytosis of *S. aureus* has been detected in isolated neutrophils in alcohol-associated cirrhosis^50^^,^^56^ and primary biliary cholangitis (PBC),^56^ as has defective phagocytosis of *E. coli* but to a lesser extent.^50^ The decrease in neutrophil phagocytosis of *E. coli* is more pronounced in patients with cirrhosis who had previous bacterial infections.^34^ Neutrophil phagocytic capacity (number of internalised bacteria per cell) of *E. coli* measured in whole blood or isolated neutrophils of patients with cirrhosis is impaired^51^^,^^52^^,^^57^^,^^59^^,^^60^ or unchanged.^61^^,^^62^ The percentage of phagocytic neutrophils is decreased in the blood of patients with cirrhosis^53^^,^^55^^,^^60^^,^^62^^,^^63^ as well as in those with ACLF;^63^ the degree of this dysfunction increases with increasing severity of cirrhosis and is higher in active drinkers compared to abstinent patients, but is not different between the aetiologies of cirrhosis (alcohol, HCV, autoimmune and other).^53^ No defect in the phagocytosis of *C. albicans* was observed in patients with cirrhosis.^48^ In ACLF, impaired phagocytosis of latex beads is reported and associated with 90-day survival.^58^ It remains to be clarified whether these subtle observed differences in phagocytosis are dependent on the method used or if they represent true differences in pathogen phagocytosis.

### Killing capacity

Bacterial killing is reduced in neutrophils from patients with cirrhosis, which means that even those active neutrophils which manage to engulf bacteria cannot efficiently kill them.^50^^,^^64^ In particular, intracellular killing of *S. aureus* and *E. coli*, which are common causes of bacterial infections in cirrhosis, is impaired in alcohol-associated cirrhosis.^50^ In contrast, another study could not find any differences in neutrophils’ capacity for intracellular killing of *S. aureus* in alcohol-associated cirrhosis and PBC despite decreased total bacterial killing, which the authors put down to the decreased percentage of phagocytic neutrophils.^56^ In another study, neutrophils from patients with cirrhosis showed impaired killing capacity not only of bacteria but also of fungi, such as *C. albicans*;^49^ however, another study found it unchanged.^48^

### ROS production

Impaired neutrophil ROS production[51], [52], [53], [54], [55]^,^^58^^,^^59^^,^[65], [66], [67], [68] and degranulation^50^^,^^64^ have been described in cirrhosis and might contribute to impaired bacterial killing.^50^ The alteration of ROS production in cirrhosis is characterised by an elevated percentage of neutrophils with basal ROS production (so-called “resting burst”)^51^^,^^55^^,^^58^^,^^60^^,^^62^^,^^65^ and elevated,^59^^,^^65^ unchanged^61^^,^^62^ or reduced^63^^,^^67^ intracellular basal ROS production (intracellular ROS levels). An increased percentage of neutrophils with basal ROS production has also been reported in ACLF.^53^^,^^58^^,^^63^ Extracellular basal superoxide production is unchanged^50^ or elevated in patients with cirrhosis.^48^

An increased percentage of neutrophils from patients with cirrhosis respond to a low physiological stimulus like fMLF,^51^^,^^52^^,^^55^^,^^62^ which suggests that these neutrophils have previously been primed by persistent low-grade stimulation with priming agents like lipopolysaccharide (LPS) or tumour necrosis factor-α (TNF-α). However, other studies show it to be unchanged.^60^ The intracellular ROS pool of those neutrophils that respond to fMLF stimulation is slightly increased,^65^ decreased^67^ or unchanged^62^ in patients with cirrhosis. Extracellular superoxide release in response to fMLF[66], [67], [68] and TNF-α^66^ is decreased in patients with cirrhosis. The decrease in superoxide release in response to fMLF is significantly more pronounced in patients with ACLF than in patients with advanced cirrhosis.^18^ Notably, priming neutrophils from patients with cirrhosis with TNF-α does not increase their response to fMLF as is usually the case with healthy donor neutrophils.^66^

The number of neutrophils producing ROS in response to a potent stimulus (*E. coli*) is unchanged^51^^,^^53^^,^^55^^,^^60^^,^^61^^,^^63^^,^^65^ or decreased^62^ in patients with cirrhosis, whereas their intracellular ROS level is increased,^65^ decreased^63^ or unchanged.^62^ In ACLF, ROS production in response to *E. coli* has been shown to be unchanged compared to that in healthy controls.^63^ Extracellular superoxide production in response to zymosan (structural component of yeast cell wall) is reduced in patients with cirrhosis.^48^^,^^50^^,^^66^^,^^69^ Extracellular hydrogen peroxide levels produced by neutrophils in response to zymosan are not different or higher in patients with cirrhosis compared to healthy controls, which is reflected in the increased hydrogen peroxide/superoxide molar ratio.^50^ Interestingly, superoxide and hydrogen peroxide have different effects on cell apoptosis and necrosis: superoxide inhibits apoptosis, whereas hydrogen peroxide promotes cell apoptosis via intracellular acidification and even necrosis when present at very high concentrations.^70^^,^^71^ Nitric oxide production in response to opsonised zymosan is also increased in neutrophils from patients with cirrhosis.^69^

Changes in ROS production are different in patients with cirrhosis with active infection: basal ROS production and ROS production in response to fMLF have been shown to be unaltered, as have the percentage of neutrophils which produce ROS in response to *E. coli* stimulation; however, the intracellular ROS pool generated in response to *E. coli* is decreased, which might support a concept that cirrhotic neutrophils are exhausted via prior low-grade stimulation and cannot mount an augmented ROS response when they have to fight a real infection.^65^

The aforementioned changes have been shown in studies on cirrhosis of different aetiologies; however, most were performed with neutrophils from patients with alcohol- or HCV-associated cirrhosis. Several studies report no dependence of ROS production on the aetiology of cirrhosis.^53^^,^^66^ Contrasting findings have been reported on the correlation of changes in ROS production with cirrhosis severity: some studies report no correlation,^51^^,^^53^ while some studies have found a correlation with disease severity.^65^^,^^66^

### Degranulation

The intracellular enzyme contents (lysozyme, myeloperoxidase [MPO]) and their release from neutrophil granules upon stimulation with zymosan have been shown to be reduced in neutrophils from patients with cirrhosis; however, the authors of this study claim that the reduced release is not dependent on the reduced enzyme levels inside the granules.^50^ A decreased number of neutrophils producing MPO and reduced intracellular MPO levels have been observed in neutrophils isolated from patients with compensated cirrhosis, but not from those with ACLF.^63^ Another study reported that intracellular MPO content is not altered in neutrophils from patients with cirrhosis but that its extracellular release in response to fMLF is decreased.^64^ MPO activity has been shown to be either decreased^66^ or unchanged^64^ in neutrophils from patients with cirrhosis. In patients with alcohol-associated cirrhosis, increased mobilisation of MPO to the cell surface of the primary neutrophil granules has been observed.^72^

### NET formation

NET formation is a recently discovered mechanism of neutrophil defence.^73^ To date, only scarce data are available regarding the role of NET formation in cirrhosis. One research group has shown a significant decrease in NET formation in response to phorbol-12-myristate-13-acetate (PMA) in patients with cirrhosis complicated by spontaneous bacterial peritonitis compared to healthy controls.^74^^,^^75^ Another group reported later that plasma of patients with decompensated cirrhosis can induce NET formation in neutrophils isolated from healthy controls.^57^ This finding is also supported by other studies that reported elevated NET markers, such as H3Cit-DNA^76^ and MPO-DNA,^76^^,^^77^ in the plasma of patients with cirrhosis and ACLF, correlating with cirrhosis severity in these patients.^76^ NET formation in response to *E. coli*, fMLF and PMA, as well as spontaneous NET formation, is elevated in patients with compensated cirrhosis and ACLF.^63^

### Apoptosis and viability

Current knowledge on neutrophil apoptosis in cirrhosis is not very broad. An increased rate of apoptosis and decreased viability of neutrophils 24 h after isolation from the whole blood of neutropenic patients with viral cirrhosis has been described.^78^ Increased apoptosis of neutrophils has also been reported in patients with decompensated cirrhosis.^79^

### Summary

Deficiencies in chemotaxis and phagocytosis, as well as decreased neutrophil viability and increased apoptosis, have been reported fairly consistently in patients with cirrhosis across studies, irrespective of differences in cohort selection, or methods of neutrophil isolation and functional assessment. Although the majority of patients had alcohol-related cirrhosis, some studies show comparable results for other aetiologies of cirrhosis, such as in study cohorts of primarily viral aetiology^31^^,^^34^^,^^60^^,^^63^ or patients with PBC.^56^ Although most studies have been performed in patients with decompensated cirrhosis, functional impairment has also been shown in patients with compensated cirrhosis.^62^^,^^63^ The findings regarding chemotaxis and phagocytosis are, however, specific to the stimulus used. For example, chemotaxis towards CXCL8, CXCL1 or casein, and phagocytosis of *C. albicans* are not impaired in cirrhosis, which might indicate that functional defects in cirrhosis are limited to certain pathways. Whether this indicates a true pathophysiological difference making patients with advanced chronic liver diseases more prone to bacterial than to fungal infections or whether this is a methodological bias needs to be further explored. In contrast, the changes in ROS production, degranulation, bacterial killing and NET formation are variable among studies. The most controversial is the nature of alterations in neutrophil ROS production in cirrhosis. Although most studies have reported an elevated number of neutrophils with basal ROS production^51^^,^^55^^,^^60^^,^^65^ and ROS production in response to physiological stimuli with low potency like fMLF,^51^^,^^52^^,^^55^ the results on ROS production are highly varied. Most studies have been performed in patients with an alcohol-related aetiology of cirrhosis and decompensated cirrhosis; however, these results are comparable to those obtained for other aetiologies and for compensated cirrhosis.^60^^,^^63^ Furthermore, some studies report no dependence of ROS production on the aetiology of cirrhosis.^53^^,^^66^ The variety of methods and experimental conditions used to study ROS production in patients with cirrhosis, often measuring different types of ROS, is the most likely explanation for these differences. The majority of studies measure neutrophil ROS production in cirrhosis with either flow cytometry (*e.g.* based on the conversion of dihydrorhodamine 123 to rhodamine 123, or similar),^58^^,^^59^^,^^63^^,^^65^ cytochrome c reduction^18^^,^[66], [67], [68] or luminol-based^67^ assays. Flow cytometry-based assays measure only intracellular ROS production, detecting a mixture of different ROS but with almost no sensitivity for superoxide (*e.g.* in the case of dihydrorhodamine).^80^ The cytochrome c reduction assay is able mainly to detect extracellular ROS and, in contrast, detects only superoxide.^81^ Luminol-based assays can be used to measure either intracellular or total ROS production (mixture of intracellular and extracellular ROS), depending on the presence or absence of horseradish peroxidase, and to detect the mixture of different ROS.^81^^,^^82^ Furthermore, the neutrophil preparation technique, whether whole blood or isolated neutrophils are used, and the type of isolation procedure may contribute to the observed differences. When the literature is separated based on methodology used for ROS production assessment, the results become more consistent (Fig. 1). To date, there is no evidence to support the notion that one method is superior in cirrhosis; however, standardisation would be necessary to harmonise the findings and enable translation of neutrophil diagnostics to clinical practice. However, even after standardisation some discrepancies will remain. Despite the methodology used to study neutrophil function, other parameters, such as patient characteristics, cirrhosis aetiology and severity, presence of previous or active bacterial infections, active alcohol drinking, neutrophil isolation technique (given the *ex vivo* fragility of neutrophils), and the combination of all these factors can contribute to the discrepancies in results between studies (Table S1).

Interestingly, recent studies have revealed the importance of neutrophil heterogeneity in different diseases. Neutrophils are no longer perceived as one cell type, but rather as several “types” of cells with particular functions and roles in health and disease. For instance, distant molecular signatures have been described for eight neutrophil subpopulations^83^ and different functional profiles have been described for five neutrophil subgroups.^84^ The role of neutrophil heterogeneity in cirrhosis and its interplay with neutrophil dysfunction are yet to be determined.

Taken together, circulating neutrophils in cirrhosis are altered numerically but, more importantly, exhibit multiple functional deficiencies, starting from the inability to migrate to the infection site and subsequent inability to internalise and kill pathogens. The loss of neutrophil antimicrobial function, particularly changes in ROS production and phagocytosis in cirrhosis, contributes to the development of bacterial infections, organ failure and mortality.^34^^,^^51^^,^^53^

This underscores the importance of identifying the mechanisms underlying neutrophil dysfunction in cirrhosis, which will allow for the development of targeted therapies for the prevention and treatment of bacterial infections in cirrhosis.

## Causes of neutrophil dysfunction in cirrhosis

First, the course of cirrhosis *per se* contributes to altered neutrophil functionality. Portal hypertension is associated with upregulation of the neutrophil chemoattractant CXCL1 in primary liver sinusoidal endothelial cells from mice and with NET formation.^85^ Alterations in Toll-like receptor (TLR)4 expression and signalling not only contribute to inflammation and endothelial dysfunction but also neutrophil dysfunction in patients with cirrhosis.^86^ Thrombocytopenia is also commonly described in cirrhosis and its crosstalk with neutrophil function has been described, *e.g.* platelet transfusions in patients with thrombocytopenia result in further increases in CD11b expression on neutrophils.^87^ Despite this, one of the important questions is whether the neutrophil dysfunction is primarily a cellular problem or if it is caused by extracellular factors. There are multiple studies that attempt to support one or other of these concepts.

### Proposed intrinsic defects

Some studies propose that cellular defects of neutrophils are responsible for their dysfunction in cirrhosis. In one study, sera from patients with decreased neutrophil locomotion does not cause a similar defect in chemotaxis in healthy control neutrophils.^30^ However, in the same study, sera from patients with cirrhosis exhibited reduced chemoattractant activity for healthy donor neutrophils compared to healthy donor serum, which could indicate that changes in serum content also potentially contribute to the decreased chemotaxis of patients’ neutrophils.^30^ Reduced intracellular glutathione levels – a detoxifier of hydrogen peroxide – are reported in neutrophils from patients with cirrhosis, which could explain the prevalence of hydrogen peroxide over superoxide. Lower levels of glutathione have been associated with higher levels of hydrogen peroxide production and lower hydrogen peroxide/superoxide ratio, degranulation and intracellular killing of *E. coli*. In this study, cirrhotic serum does not cause any changes in phagocytic function or intracellular bacterial killing of healthy donor neutrophils, suggesting underlying cellular defects, including intracellular glutathione deficiency.^50^ Dysfunctional neutrophils from patients with cirrhosis have decreased phospholipase C (which is involved in superoxide production) activity; however, the reason for this is unknown.^66^ Furthermore, changes in neutrophil glycolytic metabolism and other transcriptional profile alterations, including induction of granule genes and downregulation of cell migration and cell cycle genes have been reported in patients with decompensated cirrhosis with ACLF,^18^ and are likely to be associated with the impaired anti-pathogenic function of neutrophils.^18^^,^^63^^,^^88^

### Proposed serum defects

**Fig. 2:**
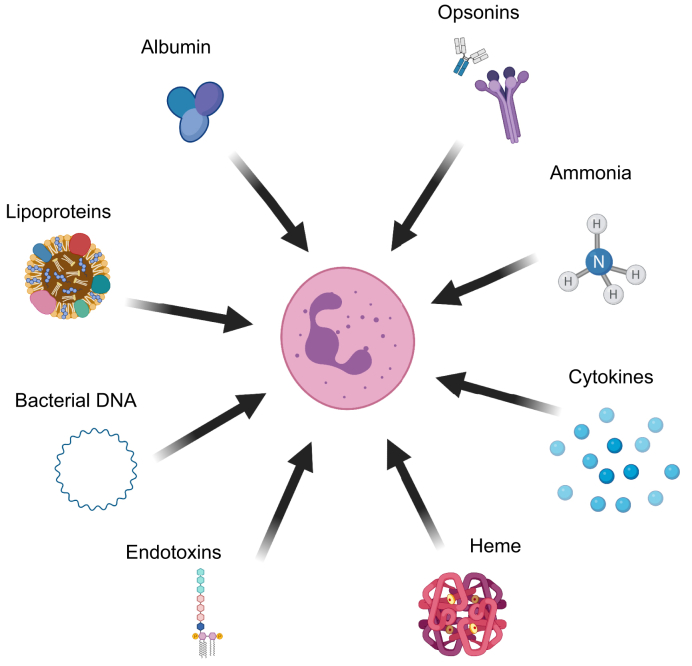
Overview on proposed serum factors that might directly or indirectly affect neutrophil function in cirrhosis. Created with Biorender.com.

The majority of studies investigating neutrophil dysfunction have proposed that changes in serum content in patients with cirrhosis initiate the development of neutrophil dysfunction (Fig. 2, Table 1). Almost all of the functional deficiencies outlined have been shown to be transferrable with patient sera. Neutrophils from patients with cirrhosis have a defect in migration towards zymosan only in the presence of autologous plasma and not healthy control plasma, and these defects vary between the aetiologies of cirrhosis (defects are present in alcohol-associated and cryptogenic cirrhosis, but not PBC).^33^ Healthy donor neutrophils have been shown to have decreased capacity to kill *C. albicans* following incubation with cirrhotic serum.^49^ Decreased phagocytic capacity for *E. coli,* but unchanged (though a tendency toward an increase) basal ROS production (percentage of neutrophils), has been shown in healthy donor neutrophils after incubation with cirrhotic plasma, dependent on cirrhosis severity but independent of cirrhosis aetiology.^61^ In another study, incubation with cirrhotic plasma promoted an increase in the number of healthy donor neutrophils with basal ROS production and a decrease in phagocytosis. Interestingly, neutrophil dysfunction in cirrhosis seems to be reversible with a restoration of function observed following incubation with healthy donor plasma.^51^ Neutrophil phagocytosis and intracellular killing of *S. aureus* are not affected in cirrhotic neutrophils incubated with AB serum despite being dysfunctional in the presence of autologous serum.^56^ Patients’ plasma also influences healthy donor neutrophil degranulation, decreasing MPO release in response to fMLF.^64^ Our research group recently showed that serum components more than 30 kD in size are responsible for the changes in neutrophil phagocytic function.^54^

**Table 1:** **Overview of serum factors thought to contribute to neutrophil dysfunction in cirrhosis**.

Serum factors	Evidence for the role in neutrophil dysfunction development	Evidence against the role in neutrophil dysfunction development
Endotoxins	• Increased basal ROS production^51^ • Decreased phagocytosis^51^ • Beneficial effects of endotoxin removal from plasma^51^	• No correlation with defect in ROS production and phagocytosis^55^^,^^61^
Bacterial DNA	• Correlation with higher levels of TNF-α, IFN-γ, IL-12 and nitric oxide^95^	• No association with severity and clinical parameters^93^ • No association with ROS production and phagocytic function^61^
Albumin	• Decreased albumin binding capacity is associated with cirrhosis severity^100^ and mortality^101^ • Decreased basal ROS production and improved phagocytosis *in vitro*^90^ • Decreased neutrophil superoxide production^105^	• No association with *C. albicans* killing capacity^49^ • No association with phagocytosis and intracellular killing capacity^50^
Ammonia	• Impaired phagocytosis of *E. coli,* increased basal ROS production^107^ • Correlation with decreased phagocytic activity^53^	
Lipoproteins	• Correlation with disease severity, increased levels of TNF-α, IL-8, IL-6^108^ • Higher levels of IgG autoantibodies against oxidised low-density lipoproteins correlated with higher integrated intracellular ROS production level in response to *E. coli*^65^	
Cytokines	• Correlation with increased numbers of neutrophils with basal ROS production and ROS producing neutrophils in response to *E. coli*.^53^ • Inhibited neutrophil phagocytosis and bactericidal activity^112^ • Improved neutrophil chemotaxis towards IL-8^42^^,^^116^ • Calprotectin is predictive for survival and infections^120^^,^^121^ • Accelerate neutrophil clearance during inflammation and impair CXCL1 production^113^ • Increase neutrophil regress from the bone marrow and their recruitment to the tissues^114^ • Trigger degranulation and NETs formation^115^^,^^118^ • Delays neutrophil apoptosis in presence of PBMCs^117^	• Calprotectin levels similar between cirrhotic patients and healthy volunteers^120^ • IL-6 does not influence neutrophil apoptosis, priming or adhesion molecule expression^116^
Iron metabolism parameters	• Delayed apoptosis of neutrophils^125^ • Activated neutrophil chemotaxis, ROS production and IL-8 expression^128^ • Impaired phagocytosis and migration of neutrophils^127^^,^^129^^,^^130^ • Reduced ROS production^131^^,^^134^ • Correlation with intracellular ROS production in response to fMLF, opsonized zymosan, PMA.^133^ • Decreased NET formation.^134^	• No effect on neutrophil ROS production, degranulation of azurophilic granules, phagocytosis and bacterial killing^134^
Serum opsonins	• Association with chemotactic inhibitory activity^35^ • Decreased opsonisation of *E. coli*^137^	• No association with neutrophil chemotaxis, phagocytosis or intracellular killing capacity defects^30^^,^^50^ • No correlation with the level of serum chemotactic inhibitory activity^32^ • No correlation with neutrophil locomotion defect^33^

CXCL1, CXC motif ligand 1; fMLF*,* N-Formyl-met-leu-phe; IFN, interferon; IL-, interleukin-; NET, neutrophil extracellular trap; PBMCs, peripheral blood mononuclear cells; PMA, phorbol-12-myristate-13-acetate; ROS, reactive oxygen species; TNF-α, tumour necrosis factor-α.

Increased gut permeability in cirrhosis results in various bacteria and bacteria-derived molecules, like endotoxins, getting from the gut lumen to the systemic circulation.^89^ These substances in the serum of patients with cirrhosis are thought to have an influence on neutrophil function via persistent low-grade stimulation.

#### Endotoxins

Endotoxins cause an increase in the percentage of neutrophils with basal ROS production in *in vitro* experiments with healthy donor neutrophils and a decrease in phagocytic capacity in experiments with cirrhotic neutrophils. Plasma endotoxin removal strategies prevent the deleterious effects of patients’ plasma on healthy donor neutrophils.^51^ However, despite *in vitro* effects of LPS on neutrophil function, the levels of bacterial endotoxin in the plasma of patients with cirrhosis do not correlate with the defects in phagocytic function^55^^,^^61^ and basal ROS production.^61^ Endotoxin receptors TLR2 and TLR4 are upregulated in healthy donor neutrophils after incubation with cirrhotic plasma^61^^,^^90^ and in neutrophils from patients with cirrhosis.^52^ TLR2 is upregulated only after incubation with plasma from patients with alcohol-associated cirrhosis, while TLR4 is upregulated in neutrophils treated with plasma from patients with both alcohol-associated and viral cirrhosis.^61^ Inhibition of TLR2 and TLR4 decreases the number of neutrophils with basal ROS production caused by incubation with cirrhotic serum, but further impairs phagocytic capacity.^90^ LPS-binding protein (LBP) levels are elevated in patients with cirrhosis compared to healthy controls, being associated with lower intracellular basal ROS production^65^ and with the development of severe bacterial infections.^91^ LBP enhances the effects of LPS on immune cells.^92^

#### Bacterial DNA

Bacterial DNA itself may also be responsible for changes in neutrophil function in cirrhosis. Bacterial DNA is present in the serum of some patients without active infection,[93], [94], [95] but is not associated with the Child-Pugh score nor clinical characteristics of these patients.^93^ The presence of bacterial DNA in the serum of patients with cirrhosis is associated with higher levels of cytokines such as TNF-α, IFN-γ, IL-12 and nitric oxide.^95^ Interestingly, higher cytokine levels in the sera of patients with bacterial DNA are independent of their LPS or LBP serum levels.^96^ In one study, the bacterial DNA sensing receptor in neutrophils, TLR9, has been shown to be more highly expressed in neutrophils from patients with cirrhosis than healthy controls.^52^ However, in other studies, TLR9 has not been found to be upregulated in patients with cirrhosis^61^^,^^90^ and circulating bacterial DNA measured in the plasma of patients with cirrhosis could neither be associated with the changes in neutrophil phagocytic and basal ROS production, nor with patient mortality.^61^

#### Albumin

Albumin is synthesised in the liver and, therefore, its concentration is reduced in cirrhosis.^97^ Furthermore, the structure of albumin is also altered in cirrhosis, mainly via oxidation, plasma levels of oxidized albumin increase further in ACLF.^98^^,^^99^ Albumin binding capacity is decreased in patients with decompensated cirrhosis, which negatively correlates with the MELD (model for end-stage liver disease) score^100^ and increased mortality.^101^ Interestingly, neutrophil to albumin ratio is linked to mortality in patients with decompensated cirrhosis.^102^^,^^103^ Given the ability of albumin to bind bacterial products, ROS, and nitric oxide,^104^ the changes in its abundance and structure might contribute to neutrophil dysfunction. The addition of albumin to incubation media has been shown to decrease the number of neutrophils with high basal ROS production and reverse phagocytic capacity defects caused by plasma from patients with cirrhosis.^90^ Carbamylated albumin dose-dependently inhibits superoxide production by neutrophils activated by type I collagen.^105^ However, one study shows that patients with cirrhosis with dysfunctional neutrophil phagocytosis and intracellular killing capacity have comparable serum albumin levels to patients with cirrhosis without neutrophil functional defects, indicating that it is not the quantity but the functionality of albumin that is important in cirrhosis-associated neutrophil dysfunction.^50^ Furthermore, the *C. albicans* killing capacity of cirrhotic neutrophils does not correlate with serum albumin levels.^49^

#### Ammonia

Ammonia levels in the serum of patients with cirrhosis predict organ failure and mortality.^106^ Neutrophils from rats subjected to ammonia supplementation and healthy donor neutrophils incubated with ammonia exhibit impaired phagocytosis of *E. coli* and an increased number of neutrophils with basal ROS production, due to the ability of ammonia to cause cell swelling.^107^ Decreased neutrophil phagocytic activity correlates with increased plasma ammonia levels in patients with cirrhosis.^53^

#### Lipoproteins

Patients with cirrhosis have lower levels of high-density lipoprotein (HDL) cholesterol and apolipoprotein A1, which further decrease upon decompensation, correlating with increased levels of TNF-α, IL-8, IL-6 and severe bacterial infection, and predicting patient mortality.^108^ The important function of HDL, similar to that of albumin, is to neutralize LPS;^109^ therefore, its low abundance in cirrhosis could be a reason for higher LPS serum concentrations and low-grade inflammation. Furthermore, HDL composition and function is altered in cirrhosis.^110^ Higher levels of IgG autoantibodies against oxidised low-density lipoproteins are correlated with higher integrated intracellular ROS production in response to *E. coli* in neutrophils from patients with cirrhosis.^65^

#### Cytokines

Elevated pro- and anti-inflammatory cytokines, such as TNF-α, IL-6, IL-1β, IL8 and IL10, have been reported in many but not all studies.^49^^,^^52^^,^^53^^,^^55^^,^^61^ Increasing numbers of neutrophils with basal ROS production correlate with increased TNF-α, IL-6, IL-8 and IL-10, whereas increasing numbers of neutrophils producing ROS in response to *E. coli* correlate with increased IL-1β, IL-8, IL-1 and IL-17 levels.^53^ Interestingly, patients with ACLF have even higher levels of TNF-α, IL-6, IL-8, IL-1β, IL-12, IL-1RA, IL-10, granulocyte colony stimulating factor (G-CSF) and granulocyte macrophage colony stimulating factor (GM-CSF) in blood compared to patients with decompensated cirrhosis.^19^^,^^44^^,^^99^ TNF-α is a known priming agent.^111^ IL-10 inhibits neutrophil phagocytosis and bactericidal activity.^112^ IL-6 has been shown, on the one hand, to accelerate neutrophil clearance during inflammation and to impair CXCL1 production (which is a chemoattractant for neutrophils) via the IL-6/gp130/STAT3 pathway;^113^ on the other hand, IL-6 has been shown to increase neutrophil regress from the bone marrow and recruitment to target tissues.^114^ IL-8 is a known neutrophil chemoattractant and has been shown to trigger degranulation and NET formation.^115^ IL-33 treatment of neutrophils from patients with cirrhosis improves their chemotaxis towards IL-8^42^ and IL-6 has been reported to increase neutrophil migration towards IL-8; however, it does not influence neutrophil apoptosis, priming or adhesion molecule expression.^116^ IL-1β delays neutrophil apoptosis in the presence of peripheral blood mononuclear cells^117^ and induces NET formation, which can be abrogated by IL-1RA.^118^ IL-17-activated pericytes produce chemokines, which stimulate neutrophil production of pro-inflammatory molecules, prolong neutrophil survival and increase neutrophil phagocytic capacity.^119^ Serum calprotectin is an important biomarker of neutrophil activation. Serum levels of calprotectin in patients with compensated and decompensated alcohol-induced cirrhosis are similar to those from healthy volunteers and are predictive of survival and recurrent infections, independent of the severity of cirrhosis.^120^ Another study has shown increased calprotectin levels in patients with stable cirrhosis and acute decompensation of cirrhosis of different aetiologies, which correlates with the severity of the disease, ACLF and infection, and is associated with poor survival in acute decompensation but not in ACLF.^121^

#### Iron metabolism parameters

Disturbances in iron metabolism are known in patients with cirrhosis.^122^ Iron metabolism parameters influence neutrophil function. Heme is a part of haemoglobin and excessive free heme is released to the circulation in the case of haemolysis,^123^ which is common for patients with cirrhosis.^60^ Hemin, which is different from heme as it contains ferric and not ferrous ion, is also increased in haemolysis.^124^ Heme delays apoptosis of neutrophils^125^ and induces migration of and ROS production by neutrophils.^126^ In contrast, another study shows impaired phagocytosis and migration of neutrophils in response to heme, which can be explained by completely different experimental design of chemotaxis experiments, with heme used not as a chemoattractant for human neutrophils *in vitro*, but as a treatment for mice in *in vivo* experiments.^127^ Hemin activates neutrophil chemotaxis, ROS production and IL-8 expression.^128^ The ferritin-containing fraction of serum significantly decreases neutrophil phagocytosis.^129^ High serum ferritin levels have been linked to impaired neutrophil phagocytosis and chemotaxis.^130^ Some authors have linked ferritin to reduced ROS formation.^131^ Higher ferritin levels in serum are associated with higher risk of bacterial infections and lower ferritin levels are associated with disease progression in patients with cirrhosis.^132^ Intracellular ROS production in response to fMLF, opsonised zymosan, or PMA is increased and correlates positively with plasma transferrin saturation but not with ferritin level in patients with hereditary hemochromatosis (liver disease severity is unclear from the paper).^133^ Furthermore, this patient cohort also shows increased neutrophil phagocytic capacity and decreased L-selectin/CD62L surface expression compared to healthy controls.^133^ Mouse models of hereditary haemochromatosis show decreased NET formation and ROS production in response to PMA, but no defect in *E. coli* phagocytosis or mobilisation of azurophilic granules.^134^ Healthy donor neutrophils pre-treated with ferrous ions or holo-transferrin decrease NET formation, but holo-transferrin does not affect neutrophil ROS production, degranulation of azurophilic granules, phagocytosis nor bacterial killing.^134^ A high-iron diet in mice results in decreased NET formation and ROS production by neutrophils in response to PMA.^134^

#### Serum opsonins

Opsonisation of bacteria helps neutrophils recognise pathogens and promotes phagocytosis and killing. The main opsonins are immunoglobulins and components of the complement system.^135^ Defects in serum opsonisation have been described in patients with cirrhosis due to deficiency of opsonisation factors.^136^ IgA, IgG and IgM have been shown to be increased in sera from patients with cirrhosis. The opsonic effects of patients’ sera on *E. coli* were found to be decreased compared to controls in this study.^137^ However, IgG, IgM and IgA levels in serum from patients with and without defects in neutrophil chemotaxis, phagocytosis or intracellular killing capacity, have not been reported to differ in some studies.^30^^,^^50^ Some studies have reported no correlation of higher IgA with the level of serum chemotactic inhibitory activity^32^ or defects in neutrophil locomotion,^33^ while another study found a correlation of increased levels of IgA and IgG with chemotactic inhibitory activity in patients with cirrhosis, and demonstrated that IgA removal restores normal chemotactic activity.^35^ Patients with cirrhosis were shown to have normal levels of C3 and C4 in one study,^56^ but decreased levels in another study.^137^ No correlation of defects in neutrophil locomotion with serum levels of C3 and C5 has been shown.^33^

#### Antibiotics

A large number of patients with cirrhosis are prescribed antibiotics; therefore, serum concentrations of antibiotics could also affect neutrophil function in these patients. Apart from their antibacterial effects, some antibiotics are also known to exhibit immunomodulatory properties. For example, ceftaroline induces CD11b and decreases CD62L expression, which tends to increase neutrophil survival in response to *S. aureus-*derived lipoteichoic acid. Vancomycin, as well as dalbavancin, teicoplanin, sulfametrole/trimethoprim and ceftazidime/avibactam have been shown to decrease CXCL8 release in neutrophils.^138^^,^^139^ Dalbavancin and teicoplanin inhibit neutrophil ROS production. Dalbavancin also inhibits neutrophil bactericidal activity. Ceftazidime/avibactam inhibit neutrophil burst in response to fMLF/cytochalasin B.^139^ Azithromycin and chloramphenicol decrease NET formation *in vitro*.^140^ Further effects of different antibiotic types on neutrophil functions are reviewed in [141], [142], [143].

#### Other factors

EMR2 (EGF-like molecule containing mucin-like hormone receptor 2) expression is increased in patients with cirrhosis, dependent on severity and the presence of bacterial infections, and is a predictor of mortality. However, ligation of EMR2 has failed to improve the phagocytic capacity of cirrhotic neutrophils despite increasing intracellular ROS production in response to *E. coli*.^59^

## Potential players in neutrophil function regulation in cirrhosis

The contribution of serum factors discussed above to cirrhosis-associated neutrophil dysfunction is rather controversial given the contrasting findings regarding their effects on neutrophils as well as their association with bacterial infections and mortality in cirrhosis. Further investigations of causes for neutrophil deficiency in cirrhosis are necessary. Particular attention should be given to serum components larger than 30 kDa.^54^

Bile acids are among these components. In systemic circulation, they are bound mainly to albumin and lipoproteins, and are therefore found in the serum fraction larger than 30 kD.[144], [145], [146] Furthermore, serum bile acids are highly elevated in liver diseases, including cirrhosis,[147], [148], [149], [150], [151], [152], [153] which makes them a potential player in the regulation of neutrophil function in cirrhosis.

Bile acids are broadly known for their functions in the gastrointestinal tract, *e.g.* cholesterol elimination and lipid emulsification. However, the bile acid receptors farnesoid X receptor[154], [155], [156] and Takeda G protein-coupled receptor 5^157^^,^^158^ have been shown to play a role in many metabolic processes and the immune response.^159^^,^^160^ Approximately 95% of bile acids are reabsorbed in the intestine by the apical sodium-dependent bile acid transporter or through passive diffusion, and around 0.5 mg of bile acids enter the systemic circulation each day.^161^

Several studies describe neutrophil function in rat or mouse cholestatic models, associating cholestasis with either increased^162^ or decreased^163^ ROS production, decreased bacterial killing,^163^ neutrophil adhesion^164^ and increased migration,^162^ unchanged^163^ or increased^162^ phagocytosis, unchanged degranulation,^163^ and increased Mac-1 expression and L-selectin shedding.^165^ Bile, unconjugated lithocholic acid, CDCA, deoxycholic acid and cholic acid (at very high concentrations) potentiate ROS release in primed rat neutrophils. Only unconjugated lithocholic acid also caused superoxide production in rat neutrophils that had not been pre-activated.^166^ The priming effect of lithocholic acid on rat neutrophils activated with PMA, fMLF or calcium ionophore has been shown, as has its inhibitory activity against beta-glucuronidase release from fMLF-activated neutrophils.^167^ However, the composition and functions of bile acids in rats are significantly different to those in humans; therefore, it is difficult to draw conclusions about bile acids’ effects on human neutrophils based on the results from rat and mouse studies.[168], [169], [170]

**Fig. 3:**
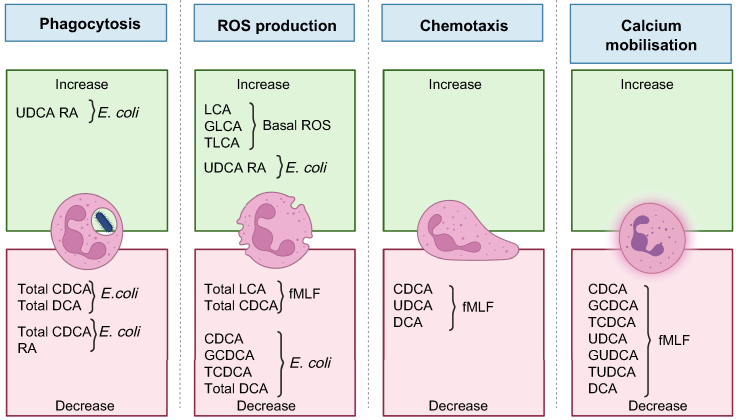
Current knowledge on how bile acids affect human neutrophil function. CDCA*,* chenodeoxycholic acid; DCA*,* deoxycholic acid; fMLF*,* N-Formyl-met-leu-phe; GCDCA*,* glycochenodeoxycholic acid; GLCA*,* glycolithocholic acid; GUDCA*,* glycoursodeoxycholic acid; LCA*,* lithocholica acid; RA*,* relative abundance in serum; ROS*,* reactive oxygen species; TCDCA*,* taurochenodeoxycholic acid; TLCA*,* taurolithocholica acid; total DCA*,* sum of deoxycholic, taurodeoxycholic and glycodeoxycholic acids; total LCA*,* sum of LCA, TLCA and GLCA; total CDCA*,* sum of CDCA, TCDCA and GCDCA; TUDCA*,* tauroursodeoxycholic acid; UDCA*,* ursodeoxycholic acid. Created with Biorender.com.

There are only a few studies describing the effects of bile acids on human neutrophils. CDCA and ursodeoxycholic acid (UDCA) serum levels have been associated with neutrophil phagocytosis and ROS production in patients with cirrhosis.^62^ Sera from patients with obstructive jaundice have been shown to induce ROS production in healthy donor neutrophils.^171^ Individual bile acids have been studied only in regard to chemotaxis, intracellular calcium mobilisation, phagocytosis and ROS production in human neutrophils. Unconjugated CDCA and UDCA have been shown to reversibly inhibit chemotaxis of human neutrophils in response to fMLF, but these effects have not been shown for lithocholic acid and cholic acid.^172^ In other studies, unconjugated CDCA, UDCA^47^ and deoxycholic acid (DCA)^173^ inhibit chemotaxis towards fMLF in human neutrophils, but DCA does not inhibit chemotaxis towards C5a or IL-8.^173^ Unconjugated and conjugated forms of CDCA, UDCA (at high concentrations)^47^ and unconjugated DCA (reversibly)^173^ have been shown to inhibit calcium flux in healthy donor neutrophils in response to fMLF, but not in response to C5a or IL-8. Bile acids differentially affect ROS production with total lithocholic acid triggering neutrophil ROS production in the absence of other stimuli. Total CDCA and lithocholic acid inhibit ROS production in response to fMLF while total CDCA and DCA inhibit ROS production and phagocytosis in response to *E. coli*^62^ (Fig. 3). Hence, the effects of bile acids on human neutrophils are not yet well described. This underscores the necessity to further investigate the effects of bile acids on neutrophils as potential contributors to cirrhosis-associated immune dysfunction.

As mentioned, bile acids are transported in the systemic circulation with the help of proteins and lipoproteins, mainly albumin.^144^^,^^145^ Albumin plays a role in neutrophil dysfunction, including defects in phagocytosis and basal ROS production,^90^ and is associated with bacterial infections in cirrhosis.^174^ Albumin has several identified bile acid-specific binding sites.^175^^,^^176^ The previously described albumin dysfunction in cirrhosis^177^ might change its affinity for bile acids. Furthermore, in conditions of albumin deficiency, bile acids change their transporter preferences to lipoproteins, which in turn might facilitate their interaction with cells and tissues, including neutrophils,^146^ and potentially cause their higher intracellular accumulation. Therefore, serum concentrations of bile acids, which are measured in cirrhosis, might underestimate the concentrations to which neutrophils are actually exposed, making bile acids that are present at relatively low serum concentrations, such as lithocholic acid and UDCA, more pathophysiologically relevant. As some lipoproteins are deficient in sera of patients with cirrhosis,^110^ the pathophysiological importance of other potential bile acid protein transporters should be considered. Therefore, serum factors responsible for cirrhosis-associated immune dysfunction should be further studied together rather than separately, as they might be interdependent.

Apart from albumin, other serum proteins could contribute directly or indirectly to neutrophil dysfunction, *e.g.* via transporting bile acids. For instance, haptoglobin is one of the most downregulated proteins in sera of patients with cirrhosis compared to healthy controls and is associated with neutrophil function.^54^ Another study shows the whole range of serum proteins which are significantly associated with neutrophil function changes in patients with cirrhosis.^57^

Furthermore, increased ANCA (anti-neutrophil cytoplasmic antibodies) levels have been observed in cirrhosis and were associated with disease severity and risk of infections.^178^ Moreover, ANCA induce NET release,^179^^,^^180^ suggesting their possible contribution to the development of defects in neutrophil function in cirrhosis.

## Proposed and potential therapeutic strategies

The search for ways to prevent and treat the development of cirrhosis-associated immune dysfunction, including neutrophil dysfunction, is ongoing. The reversibility of neutrophil dysfunc-tion reported in several studies^49^^,^^51^ suggests that it could be possible to develop therapeutic strategies aimed at restoration of neutrophil function.

### Therapeutic strategies targeting neutrophil dysfunction in cirrhosis supported by clinical studies

Previous studies have shown the beneficial effects of albumin administration on overall survival and bacterial infection rates in patients with cirrhosis;^181^^,^^174^ however, its effects on neutrophil function have not been investigated within clinical studies. *In vitro* experiments have hinted at the possibility of restoring neutrophil function with albumin treatment.^90^ There is also indirect clinical evidence of possible albumin-related effects on neutrophil functionality, as some studies reported decreased plasma cytokine levels,^174^^,^^182^^,^^183^ which have been shown to correlate with changes in neutrophil ROS production,^53^ following albumin treatment. Albumin appears to be a promising immunomodulating strategy in cirrhosis, though more clinical evidence is required.^184^

*Ex vivo* studies assessing the removal of endotoxin from the sera of patients with cirrhosis support the hypothesis that this can be a potential therapeutic approach to combat neutrophil dysfunction in cirrhosis.^51^ We have identified only one study to date that explored the effects of endotoxin removal from the plasma of patients with cirrhosis; however, they have not yet reported whether they observed any effects on neutrophil function.^185^

Further strategies suggested in cirrhosis include treatment with G-CSF or GM-CSF. It has been shown in *ex vivo* studies, for instance, that treatment with either G-CSF or GM-CSF can restore neutrophil’s ability to inhibit *C. albicans* growth^49^ and that G-CSF can enhance neutrophil transendothelial migration.^31^ Despite conflicting results regarding the effects of G-CSF on patients’ survival and liver function, clinical evidence of the beneficial effects of G-CSF on neutrophil function in acute liver failure exist, indicating that treatment with G-CSF can increase neutrophil phagocytosis, killing and ROS production.^186^ Several studies have shown that G-CSF therapy decreases the occurrence of infections in patients with cirrhosis, indicating immunomodulatory effects.[187], [188], [189] Interestingly, beta-blockers have recently shown potential to improve neutrophil phagocytic capacity in cirrhosis.^190^

Given the existing interactions between gut microbial composition and neutrophil function,^191^ probiotic interventions have been examined for their indirect modulating effects on neutrophil function. However, the results of clinical trials are conflicting, indicating either effects of probiotic supplementation only on the phagocytic capacity of neutrophils,^52^ or on neutrophil ROS production,^55^ or no effect.^192^ The origin of conflicting findings might be different sample sizes and different probiotics used for supplementation. A detailed overview of clinical studies is presented in Table 2.

**Table 2:** **Clinical studies that evaluated different therapeutic strategies in cirrhosis with potential effects on neutrophil function**.

Study	Intervention	Participants	Primary endpoint	Neutrophil-related effects	Overall effects on disease course	Adverse events
**Direct modulation**						

Caraceni P *et al.*, 2018^181^ NCT01288794	Human albumin (intravenously 40 g twice weekly for 2 weeks, and then 40 g weekly) + standard medical treatment for up to 18 months *vs.* standard medical treatment	431 patients with cirrhosis and uncomplicated ascites	18-month mortality	Not tested	18-month survival is significantly higher in patients with albumin supplementation	No adverse events attributed to albumin treatment
Fernández J *et al.*, 2020^174^ NCT02034279	Antibiotics plus albumin or antibiotics alone; 1.5 g/kg body weight at Day 1 and 1 g/kg body weight at Day 3 up to a maximum of 150 g and 100 g, respectively, in patients with body weight >100 kg, and a minimum of 90 g and 60 g, respectively, in patients with body weight <60 kg. Then days 3 and 7, and weekly until infection resolution	118 patients with advanced cirrhosis and non-SBP infections	In-hospital mortality	Decreased plasma IL-6 in patients with albumin supplementation	Less nosocomial infections in patients with albumin supplementation	No adverse events attributed to albumin treatment
Fernández J *et al.*, 2019^183^ NCT00968695 NCT03451292	Long-term: 12 weeks treatment with low doses (1 g/kg body weight every 2 weeks) and high doses (1.5 g/kg every week) of albumin; Short-term: 1-week treatment with antibiotics alone or the combination of albumin and antibiotics (1.5 g/kg on day 1 and 1 g/kg on day 3)	18 patients without bacterial infections (the Pilot-PRECIOSA study) with long-term albumin treatment; 78 patients with bacterial infections (INFECIR-2 study) with short-term albumin treatment		Reduced IL-6, G-CSF, IL-1 RA, vascular endothelial growth factor upon high-dose albumin treatment	Increased serum albumin level, improved circulation stability and left ventricular function	
Guevara M *et al.*, 2012^212^ NCT 00124228	Antibiotics and albumin (1.5 g/kg at diagnosis and 1 g/kg at day 3) or antibiotics alone	110 patients with cirrhosis and infections other than SBP	Survival at 3 months	Not tested	Improved renal and circulatory function, survival at 3 months did not change, but albumin is predictive for survival	
Sola E *et al.*, 2018^213^ NCT00839358	One-year treatment with midodrine and albumin (40 g/15 days) or placebos	196 patients with cirrhosis and ascites awaiting liver transplantation	Incidence of any complication (renal failure, hyponatremia, infections, hepatic encephalopathy or gastrointestinal bleeding)	No significant changes in plasma levels of IL-1β, IL-6 and TNF-α	No difference in the probability of developing complications of cirrhosis or one-year mortality	No adverse events attributed to albumin treatment
China L *et al.*, 2021^214^	Albumin treatment for 2 weeks or until discharge. Median total infusion of albumin of 200 g	777 patients with cirrhosis	New infection, kidney dysfunction, or death between days 3 and 15 after the initiation of treatment	Not tested	No significant difference in primary endpoint	More severe adverse events in albumin treated group
Chen *et al.*, 2009^182^	Antibiotics and albumin infusion (20% 50 cc every day for 3 days) or antibiotics alone	30 patients with cirrhosis with SBP, 24 patients with cirrhosis with sterile ascites		Reduced plasma levels of IL-6 and TNF-α, ascitic fluid levels of IL-6, TNF-α, endotoxin, unchanged levels of nitric oxide products		No adverse events attributed to albumin treatment
Agarwal *et al.*, 2021^185^ NCT03065699	DIALIVE (removes endotoxin from plasma) is administered for a median of 3 sessions (range 1–5) in first 3-days (range 1–6) for a median of 8 h (7–12) each day or standard of care	32 patients with ACLF and alcohol-associated cirrhosis	Safety	Not reported	Increases proportion of patients resolving ACLF and reduces time to resolution, significantly lower MELD score	
Rolando *et al.*, 2000^186^	G-CSF to four groups (each *n* = 6) of ALF patients; a daily infusion at 25, 50, 100 or 150 μg/m^2^ for 2 weeks	24 patients with ALF receiving G-CSF and 8 ALF patients which did not receive G-CSF	Neutrophil phagocytosis and killing of *Staphylo-coccus aureus* and superoxide production at 24 and 96 h after G-CSF administration	50, 100 or 150 μg/m^2^ G-CSF treatment resulted in significantly increased phagocytosis and killing at 96 h. 50 and 150 μg/m^2^ of G-CSF resulted in increased superoxide production at 96 h	Not reported.	Two patients receiving 150 μg/m^2^ G-CSF treatment had adverse effects possibly related to G-CSF: leucocytosis and increase in gamma glutamyl transpeptidase, aspartate aminotransferase, alkaline phosphatase on last day of G-CSF administration
Sehgal *et al.*, 2022^215^	250 μg of GM-CSF intravenously for about 6 h daily for 5 days	164 decompensated patients with cirrhosis with or without sepsis and 15 healthy controls		Neutrophil count is decreased after 1 day of GM-CSF therapy	Improved survival	
Venkitaraman *et al.*, 2022^187^ NCT03911037	G-CSF 5 μg/kg subcutaneously, 12 hourly for 5 consecutive days, a total of 4 cycles, once every 3 months	70 patients with decompensated cirrhosis	12-month overall survival	Not studied, but decreased rate of infections observed upon treatment	Survival not improved	Treatment-related adverse events in 23 patients, mostly backache
Kedarisetty *et al.*, 2015^216^ NCT01384565	Subcutaneous G-CSF (5 μg/kg/d) for 5 days and then every third day (12 total doses) and subcutaneous darbopoietin α (40 mcg/wk) for 4 weeks	55 patients with decompensated cirrhosis	Survival at 12 months	Not studied	Higher survival in patients upon treatment	No adverse events attributed to G-CSF treatment
Prajapati *et al.*, 2017^217^ NCT02642003	GCSF 300 μg twice daily for 5 days	126 patients with decompensated cirrhosis with GCSF plus standard medical therapy and 127 patients with decompensated cirrhosis and only standard medical therapy	Number of participants alive at 6 months	Not studied	Higher cumulative survival in patients with GCSF treatment	No adverse events attributed to G-CSF treatment
Newsome *et al.*, 2018^218^ 2009-010335-41	Subcutaneous injections of G-CSF (lenograstim; Chugai Pharmaceuticals, London, UK) at 15 μg/kg bodyweight daily for 5 consecutive days	26 cirrhotic patients with G-CSF, 28 cirrhotic patients with G-CSF plus stem-cell infusion, 27 cirrhotic patients with standard care	Change in MELD score at 90 days from baseline and the trend of treatment activity established by incorporating MELD score measured at baseline and days 30, 60 and 90	Not studied	No improvement in MELD score	No adverse events attributed to G-CSF treatment
De *et al.*, 2021^188^ NCT03415698	5 days of G-CSF (5 μg/kg subcutaneously every 12 h) every 3 months, with standard medical therapy, in 4 cycles or standard medical therapy alone	100 patients with decompensated cirrhosis (50 patients on G-CSF plus standard medical therapy, 50 patients on standard medical therapy)	Survival at 12 months	Not studied, but recorded fewer infections in the group treated with G-CSF	Higher survival at 12 months	Adverse effects of G-CSF in 37 patients, with the most common being fatigue (46%) and back pain (46%). Patients treated with G-CSF had leucocytosis on day 6 of each treatment cycle
Verma *et al.*, 2018^189^ NCT02451033	G-CSF (5 μg/kg subcutaneously every 12 h for 5 days, then every 3 months for 3 days until 12 months; four cycles)	65 patients with decompensated cirrhosis. 23 patients on SMT plus G-CSF plus growth hormone, 21 patients on SMT plus G-CSF, 21 patients on SMT alone	Transplant-free survival at 12 months	Not studied, but less infections after G-CSF treatment	Higher survival at 12 months	The majority of adverse effects related to G-CSF including back pain, fatigue, bone pains and fever
Spahr *et al.*, 2008^219^	5 days of G-CSF (10 μg/kg/day)	24 patients with alcohol-associated cirrhosis and alcoholic steatohepatitis; 13 patients receiving G-CSF plus SMT, 11 patients receiving only SMT	CD34 stem cell mobilisation, liver cell proliferation and liver function	Not studied. Treatment did not affect the level of circulating neutrophils, TNF-α, sTNF-R1 and IL-6 not affected; HGF level increased upon the treatment course	No improvement in liver function	Three patients complained of transient mild lower back pain reversible when G-CSF treatment ended
Gaia *et al.*, 2013^220^	3-day G-CSF 5 lg/kg every 12 h at 3-month intervals for four courses	15 patients with advanced cirrhosis	Bone marrow-derived cells mobilisation	Not studied	No effect on survival, improved Child–Pugh score	No severe adverse events, complaints about bone pain in four patients

**Indirect modulation**						

Macnaughtan *et al.*, 2020^192^	65 mL bottle of Probiotic *Lactobacillus cas*ei Shirota (6.5 × 10^9^ colony forming units (CFU)/bottle 3 times daily for 6 months	92 patients with cirrhosis; half of the patients received probiotic, half received placebo	Incidence of significant infection and neutrophil function	Rates of infection and neutrophil function (ROS production and phagocytosis) did not change upon treatment; plasma monocyte chemotactic protein-1 and IL-1β (in alcohol-associated cirrhosis), IL-17a and macrophage inflammatory protein-1β (in non-alcoholic cirrhosis) decreased	No significant effects	No serious adverse effects related to probiotic intervention
Horvath *et al.*, 2016^55^	Once-a-day a dose of a multispecies probiotic (*Bifidobacterium bifidum* W23, *Bifidobacterium lactis* W52, *Lactobacillus acidophilus* W37, *Lactobacillus brevis* W63, *Lactobacillus casei* W56, *Lactobacillus salivarius* W24, *Lactococcus lactis* W19 and *Lactococcus lactis* W58) 6 g, 2.5 × 10^9^ CFU/g for 6 months	92 patients with cirrhosis, 45 patients treated with probiotic	Change in phagocytic capacity of neutrophils between baseline and 6 months	Increased neutrophil basal ROS production, no effect on neutrophil phagocytosis	More patients in probiotic group improved their Child–Pugh score although only non-significant improvement in MELD score	Adverse events possibly related to the probiotic (in 41% of patients treated with probiotic): flatulence, gastric pain, diarrhoea and nausea
Stadlbauer *et al.*, 2008^52^	*Lactobacillus casei* Shirota (6.5 × 10^9^) 3 times daily for 4 weeks	20 patients with alcohol-associated cirrhosis, 12 patients with cirrhosis received probiotic treatment	Neutrophil ROS production, phagocytosis, toll-like-receptor expression, plasma cytokines and *ex vivo* endotoxin-stimulated cytokine production	Probiotics restored neutrophil phagocytic capacity, no effect on neutrophil ROS production	No changes	No adverse events

CFU, colony-forming unit; G-CSF, granulocyte colony stimulating factor; IL-, interleukin-; MELD, model for end-stage liver disease; ROS, reactive oxygen species; SMT, standard medical therapy; TNF-α, tumour necrosis factor-α.

### Potential therapeutic strategies of neutrophil dysfunction in cirrhosis

Other potential therapeutic strategies include modulating bile acid composition via oral intake of bile acids, *e.g*. UDCA or cholic acid, or modulating gut microbial composition (as bile acids are metabolites of gut microbiota and the gut microbiome shapes serum bile acid composition^193^) via probiotic supplementation.

A range of bile acids including UDCA and obeticholic acid have already been approved for use in clinical settings, representing a potential treatment option for neutrophil dysfunction in cirrhosis given the altered serum bile acid composition in patients with cirrhosis and *in vitro* evidence of bile acid effects on neutrophil function.^62^ However, to date, there have been no clinical studies on the effect of bile acid supplementation on neutrophil dysfunction either in cirrhosis or in any other diseases or healthy volunteers.

Furthermore, the normalisation of circulating bile acid composition can be achieved indirectly by modulating the composition of the gut microbiome. Different bacteria have been shown to be associated with bile acid metabolism. For example, Akkermansia abundance is affected by bile acids,^194^ genus *Prevotella* is associated with plasma bile acid composition^195^ and genus *Streptococcus* is involved in primary bile acid metabolism.^196^^,^^197^ UDCA is a result of 7α/β-isomerisation of CDCA by gut microbiota,^193^ *e.g.* by *Clostridium absonum*,^198^ *Clostridium baratii*^199^ and strains from genera *Eubacterium* and *Ruminococcus*.^200^ Therefore, a desired decrease in toxic CDCA, which is highly elevated in sera of patients with cirrhosis, can potentially be reached via the modulation of microbial composition, *e.g.* with probiotic supplements, which aims at restoration of the balance in abundance of bacteria with 7α- and 7β-hydroxysteroid dehydrogenase activity and, thus, for epimerisation of CDCA to UDCA.^201^ Besides, this might be achieved by modulating the abundance of bacteria with bile salt hydrolase activity, which is responsible for bile acid deconjugation and was previously suggested to play a role in epimerisation.^199^ These bacteria include *Bacteroides ovatus*,^202^ *Clostridium perfringens*,^203^ *Enterococcus faecalis*^204^ and different strains of *Bifidobacterium*^205^ and *Lactobacillus*.^206^^,^^207^ Administering a combination of bacterial strains with bile salt hydrolase activity and 7α-/7β-hydroxysteroid dehydrogenase activity in order to increase UDCA production has already been proposed.^208^

Another potential therapeutic strategy to treat neutrophil dysfunction in cirrhosis may be the inhibition of PD1 (programmed cell death 1) and TIM3 (T-cell immunoglobulin and mucin domain-containing protein 3) receptors, which mediate immunosuppression. *Ex vivo* studies have shown that the presence of antibodies blocking PD1 and TIM3 increases neutrophil antimicrobial activities, such as phagocytic capacity and ROS production, in response to *E. coli*.^209^ Furthermore, strategies involving liver assist devices can potentially improve neutrophil function in cirrhosis by removing endotoxin, oxidised albumin^210^ and cytokines^211^ from the circulation.

## Conclusion

Neutrophil dysfunction is a recognised feature of cirrhosis that predisposes patients with cirrhosis to bacterial infections and increases their mortality rate. Despite extensive studies in recent decades, significant knowledge gaps and problems remain to be solved, which are summarised in Table 3. There is a need for standardisation of the approaches used to assess different neutrophil functions. There is also a need to develop neutrophil function screening panels that are useful for clinical studies and clinical practice as most of the currently available methodologies to study neutrophil function are extremely time- and labour-consuming and performer-sensitive due to the short lifespan and fragility of neutrophils *ex vivo*. In particular, more knowledge on recently described neutrophil behaviour, like NET formation or swarming, as well as on the molecular mechanisms involved in the development of neutrophil dysfunction, are needed. Furthermore, there is still a vague understanding of the best strategy to prevent and treat neutrophil dysfunction in cirrhosis, mostly because of the lack of clinical studies with neutrophil function as a primary or secondary outcome. So far, albumin supplementation and G-CSF have the most clinical evidence; however, although clinical benefits have been noted, a clear causal relationship between these therapies and improvement of neutrophil function needs to be established. Last but not least, it will be important to study neutrophil dysfunction in patients with cirrhosis as a multifactorial problem. Studies that investigate the potential causative factors of neutrophil dysfunction not individually but interdependently, *e.g.* the interdependent role of serum proteins, lipoproteins and bile acids, are needed. This will improve our understanding of the mechanisms behind defects in neutrophil function and guide efforts to improve the prevention and treatment of neutrophil dysfunction, decrease the development and consequences of bacterial infections and, ultimately, improve quality of life and survival in cirrhosis.

**Table 3:** **Current gaps and future research areas**.

Issue	Description of the problem	Solution and future research
Standardisation	Methods to study neutrophil function vary, which leads sometimes to a “wrong” perception of conflicting findings	Developing standardised neutrophil function panel, standardisation in the description of methods and results in original and review papers
Methodology	Lack of clinically useful neutrophil function tests	Developing standardised neutrophil function panel applicable in clinical settings
Diagnosis	No widely accepted biomarker of neutrophil dysfunction	Development and validation of biomarker of neutrophil dysfunction
Clinical studies	Lack of clinical studies investigating therapies of neutrophil dysfunction in cirrhosis with neutrophil function as primary or secondary outcome	Including neutrophil function as an outcome in clinical studies (albumin, G-CSF supplementation and other potential therapeutic approaches)
Knowledge gaps	Some of the neutrophil functions are not well described in cirrhotic patients (*e.g.* NET formation, swarming), causative factors and molecular mechanisms of neutrophil dysfunction are still not fully deciphered	Further studies of neutrophil functions, causative factors and molecular mechanisms involved in neutrophil dysfunction development in cirrhosis
Multifactorial	Studying causative factors, mechanisms and treatment approaches individually might not provide all answers, as neutrophil dysfunction in cirrhosis is a multifactorial problem	Perform studies of potential causative factors and mechanisms of neutrophil dysfunction in cirrhosis in complex experimental study setups rather than each factor individually

NET, neutrophil extracellular trap.

## Financial support

V.S. received funding from the Austrian Science Fund (KLI 741). The project was in part conducted at the Center for Biomarker Research in Medicine (CBmed), a COMET K1 centre funded by the 10.13039/501100004955Austrian Research Promotion Agency (Project 3.23), I.B.'s work was in part funded through the Doctoral College “Molecular Fundamentals of Inflammation” (W1241).

## Authors’ contributions

Conceptualization, V.S.; writing—original draft preparation, I.B. and V.S; writing—review and editing, V.S. and I.B.; visualization, I.B.; supervision, V.S.; funding acquisition, V.S. All authors have read and agreed to the published version of the manuscript.

## Conflicts of interest

The authors declare no conflict of interest.

Please refer to the accompanying ICMJE disclosure forms for further details.
