# Supplementary material for: Circulating neutrophil anti-pathogen dysfunction in cirrhosis
Source: JHEP Rep. 2023 Aug 1;5(11):100871. doi: 10.1016/j.jhepr.2023.100871 (PMC10562928; doi:10.1016/j.jhepr.2023.100871)
Supplement: Multimedia component 1 [file mmc1.pdf]

Circulating neutrophil anti-pathogen dysfunction in cirrhosis

Irina Balazs, Vanessa Stadlbauer

Table of contents

Table S1..2

Supplementary references...15

Table S1. Overview of neutrophil functional defects in patients with cirrhosis including patients 'demographics, liver cirrhosis severity and etiology, neutrophil isolation technique and methodology used to study neutrophil function.

Study	Patient demographics	Severity and etiology of cirrhosis	Bacterial infections	Neutrophil isolation	Method	Result
**Chemotaxis**						
Rajkovic *et al.,* 1984 [1]	48 patients alcohol associated liver disease – 45 patients with cirrhosis, 12 patients with acute alcoholic hepatitis, 3 patients with fatty change of liver (27-66 years old; 27 males, 21 females); 21 healthy controls	alcohol	7 patients with bacterial infections	Ficoll-Triosil gradient centrifugation	“Leading front” technique	Reduced migration towards healthy serum as chemoattractant compared to healthy donor neutrophils. No correlation with bacterial infections.
Fiuza *et al.,* 2000 [2]	22 cirrhotic patients with ascites (15 male, 7 female; age 55.8+/-10.4); 6 healthy volunteers	Child-Pugh B- 10 patients, Child-Pugh C – 12 patients; etiology: viral (50%), alcohol (22%), both alcohol and viral (18%), unknown (9%)	12 patients with 1 or more episodes of bacterial infection in the 6 months before inclusion in the study	Not applicable	Skin window technique	Patients with cirrhosis have a decreased neutrophil migration into skin windows filled with gram-negative or gram-positive bacteria and it correlates with the liver dysfunction severity and presence of previous infections.
Onishi *et al.,* 1989 [3]	17 patients with alcohol associated liver disease including 9 patients with liver cirrhosis (1 female, 8 male; age 38-62 years old); 16 patients with non-alcoholic liver cirrhosis (8 female, 8 male; age 31-75 years old); 18 healthy volunteers	Alcohol and non-alcoholic	-	Dextran sedimentation	Modification of a Boyden technique using chemotactic chambers	Serum chemotactic inhibitory activity higher in alcoholic liver disease compared to healthy controls and higher in alcohol associated cirrhosis compared to non-alcoholic cirrhosis
Van Epps *et al.,* 1975 [4]	42 patients with alcohol associated liver disease (37 male, 5 female, age 23-68 years old) including 38 patients with cirrhosis, 20 patients with alcoholic hepatitis, 4 patients with fatty change.	Alcohol	9 patients with active infection	Plasma gel sedimentation of erythrocytes	Boyden technic	Chemotactic inhibitory activity is in 21 of 42 patients and does not correlate with the type of liver disease and degree of liver function, but is associated with infections
Campbell *et al.,* 1981 [5]	44 patients with alcohol associated liver disease (22 male, 22 female; age 17-81), including 31 patients with cirrhosis	Alcohol (13 patients), chronic active hepatitis (10 patients), primary biliary cirrhosis (8 patients), cryptogenic cirrhosis (13 patients)	-	Dextran sedimentation	“Leading front” technique	Neutrophil migration reduced in patients with alcohol related liver disease in presence of autologous plasma, but not healthy donor plasma; depressed migration, but to a lesser extent, in patients with cryptogenic cirrhosis, but not in patients with chronic active hepatitis or

						primary biliary cirrhosis
Fiuza *et al.*, 2002 [6]	14 cirrhotic patients (56.42 ± 11.62 years; 9 male, 5 female); 14 healthy controls; no patient consuming alcohol at the time of the study	Child-Pugh classes B (6 patients) and C (8 patients); hepatitis C (6 patients), alcohol (3 patients), hepatitis B (3 patients), Budd-Chiari syndrome (1 patient), hepatitis C+alcohol (1 patient)	No infections within the preceding week	Dextran sedimentation and gradient centrifugation	Transwell inserts with 3um pores seeded with HMEC-1 cells monolayers; neutrophils migrated through the pores; neutrophils counted with hemacytometer .	Decreased transendothelial migration towards fMLF of neutrophils from cirrhotic patients
Artru *et al.*, 2020 [7]	24 patients with cirrhosis (19 male, 5 female; age 52.7 – 66.9)	Child-Pugh score 7-11; alcohol	-	MACSxpress Neutrophil isolation human kit	Transwell inserts with 3um pores, migrated neutrophils counted with flow cytometry	Decreased neutrophil migration towards IL-8 in cirrhotic patients
Claria *et al.*, 1998 [8]	12 patients with cirrhosis and ascites; 13 healthy donors	-	Patients without evidence of bacterial infections	Ficoll-Hypaque gradient centrifugation, Boyum method	Transwell inserts with 3um pores, migrated neutrophils are counted by lysing cells with 0.025 mol/L NaOH with 0.1% SDS and then measuring the fluorescence	Decreased migration of neutrophils isolated from patients with cirrhosis in response to leukotriene B4
Langer *et al.*, 2023 [9]	11 patients with compensated cirrhosis (mean age 53.18; 6 male, 5 female), 84 patients with acute decompensation (AD) of cirrhosis without acute-on-chronic liver failure (ACLF) (mean age 54.87, 46 male, 38 female), 30 patients with ACLF (mean age 56.7; 20 male, 10 female); 24 healthy controls	Compensated (Child-Pugh score mean 5.27), AD (Child-Pugh score mean 8.54), ACLF (Child-Pugh score mean 9.37); etiologies: viral, non-alcoholic steatohepatitis (NASH), alcohol, cholestatic, others	AD – 26 patients with infections, ACLF – 19 patients with infections	MACSxpress® Whole Blood Neutrophil Isolation Kit	96-well flat-bottom plates in X-Vivo 10 Medium (Lonza) is used; neutrophil migration is counted by imaging with a Leica DMI6000 B (Leica Microsystems)	Decreased migration towards fMLF (proportion of migrating neutrophils) in patients with AD of cirrhosis and ACLF, correlated with severity of cirrhosis
Laffi *et al.*, 1993 [10]	10 cirrhotic patients (5 female, 5 male; age 42-65); 10 healthy controls	Child-Pugh B (4 patients), Child-Pugh C (6 patients); hepatitis C (8 patients), hepatitis B (1 patient), hepatitis B and C (1 patient)	No infections	Ficoll-Hypaque gradient	Modification of a Boyden technique using chemotactic chambers	No neutrophil chemotaxis defect in response to casein in cirrhosis; no differences in random migration of neutrophils from cirrhotic patients

Knooihuizen *et al.,* 2021[11]	21 patients with cirrhosis, 23 healthy controls	Alcohol (57%), non-alcoholic fatty liver disease (29%), hepatitis C virus (14%), other (19%); 6 patients with AD, 15 patients had ACLF	4 patients with spontaneous bacterial peritonitis	EasySep Direct Human Neutrophil Isolation Kit	Time-lapse imaging of swarming performed with Nikon Ti-E microscope	Swarming of cirrhotic patients' neutrophils in order to control Candida albicans hyphae growth is impaired compared to healthy controls
**Phagocytosis**						
De Fernandez *et al.,* 1987 [12]	57 patients with liver disease; no significant alcohol consumption within 10 days before study	19 males and 3 females with alcohol associated liver disease (mean age 54) including 14 patients with cirrhosis; 2 males and 19 females with primary biliary cirrhosis (mean age 60.4); 5 male and 9 female patients with chronic active hepatitis (mean age 65) of mainly autoimmune etiology;	All patients clinically free from infections at the time of study	Dextran sedimentation	Neutrophils are incubated with *S.aureus* and phagocytosis is calculated as original number of *S.aureus* minus viable extracellular *S.aureus* after incubation divided by the original number of *S.aureus* and multiplied by 100	Defective phagocytosis in alcohol associated liver disease and primary biliary cirrhosis, but not chronic active hepatitis
Rajkovic *et al.,* 1986 [13]	40 patients with alcohol associated liver disease (39-66 years), all had cirrhosis, including 18 patients with superimposed acute alcoholic hepatitis; 20 healthy controls	alcohol	No active bacterial infection at inclusion	Dextran sedimentation and Ficoll-Hypaque gradient centrifugation	Neutrophils are incubated with bacteria in 96 well plate with added [^{3}H]uridine; phagocytosis index is calculated based on the radioactivity measurements	Defective phagocytosis of *S.aureus* and *E.coli* in neutrophils from patients with alcohol associated cirrhosis, no association with disease severity
Fiuza *et al.,* 2000 [2]	22 cirrhotic patients with ascites (15 male, 7 female; age 55.8+/-10.4) and 6 healthy volunteers	Child-Pugh B - 10 patients, Child-Pugh C – 12 patients; etiology: viral (50%), alcohol (22%), both alcohol and viral (18%), unknown (9%)	12 patients with 1 or more episodes of bacterial infection in the 6 months before inclusion in the study	Not applicable	Skin window technique, phagocytosis assessed with flow cytometry	Phagocytosis of *E.coli* in neutrophils from cirrhotic patients is impaired compared to healthy controls; the decrease in phagocytosis of *E.coli* is more pronounced in patients with previous bacterial infections and correlates with the severity of liver dysfunction
Mookerjee *et al.,* 2007 [14]	63 patients with AD of alcohol associated cirrhosis; 20 healthy controls	Alcohol cirrhosis and superimposed inflammatory alcoholic hepatitis	No clinical or microbiological evidence of infection	Not applicable	The Phagotest kit (Orpegen Pharma) with whole blood	Neutrophil phagocytic capacity of *E. coli* is impaired in cirrhosis (cirrhosis +alcoholic hepatitis)
Stadlbauer *et al.,* 2008 [15]	20 patients with alcohol associated cirrhosis (18-75 years; 15 male, 5 female); 13 healthy controls	Alcohol	No clinical or microbiological evidence of infection	Not applicable	The Phagotest kit (Orpegen Pharma) with whole blood	Reduced neutrophil phagocytic capacity of *E.coli* in cirrhotic patients
Huang *et al.,* 2016 [16]	48 healthy volunteers and 100 cirrhotic patients (77 male, 23	26 patients with Child-Pugh A, 26 patients with Child-Pugh B, 48 patients in Child-Pugh C; Etiologies:	-	Not applicable	The Phagotest kit (Orpegen Pharma) with whole blood	Reduced neutrophil phagocytic capacity of *E.coli* in cirrhotic patients of Child-Pugh C

	female; mean age: 55 ± 13 years)	alcohol, hepatitis B and hepatitis C				
Sehgal *et al.,* 2022 [17]	70 patients with decompensated cirrhosis (63 male, 7 female; age 22-60); 15 healthy controls	Decompensated cirrhosis; alcohol predominant etiology (70%)	40 patients with sepsis	Not applicable	The Phagotest kit (Celonic) with whole blood	Reduced neutrophil phagocytic capacity of *E.coli* in cirrhotic patients
Horvath *et al.,* 2016 [18]	80 patients with cirrhosis (mean age probiotics group 60, placebo group 56; 58 male, 22 female); 51 healthy controls; no alcohol abuse within 2 weeks before the study	Alcohol (44), hepatitis C (13), other (22); Child-Pugh A (61), Child-Pugh B+C (19)	No active infection at screening	Not applicable	The Phagotest kit (Glycotope) with whole blood	Percentage of phagocytic neutrophils is decreased in blood of cirrhotic patients
Taylor *et al.,* 2014 [19]	62 patients with cirrhosis (age 39-61; 17 female)	Stable cirrhosis (49), ACLF (13); etiologies: alcohol, hepatitis C, autoimmune liver disease, other	7 patients with active infection in ACLF group	Not applicable	The Phagotest kit (Orpegen Pharma) with whole blood	Percentage of phagocytic neutrophils is decreased in blood of cirrhotic patients; degree of this dysfunction is increasing with increasing severity of cirrhosis, but is not different between the etiologies of cirrhosis
Wu *et al.,* 2021 [20]	18 patients with hepatitis B virus related ACLF, 14 patients with compensated liver cirrhosis, 13 healthy controls	Hepatitis B virus ACLF or compensated liver cirrhosis	No active bacterial infection	Not applicable	The Phagotest kit (Glycotope) with whole blood	Decreased percentage of phagocytosing neutrophils (*E.coli*) in both compensated cirrhosis and ACLF, with the more pronounce defect in ACLF. Phagocytic activity is significantly decreased in ACLF compared to healthy controls.
Balazs *et al.,* 2022 [21]	109 cirrhotic patients (mean age alcohol 56, hepatitis C 60, other 55; alcohol 42 male, 12 female, hepatitis C 22 male, 10 female, other 13 male, 10 female); 21 healthy controls	Alcohol (54 patients), hepatitis C (32 patients), other (23 patients); Child-Pugh A (79 patients), Child-Pugh B+C (30 patients)	No active bacterial infection	Not applicable	The Phagotest kit (Celonic) with whole blood	Percentage of phagocytic neutrophils is decreased in blood of cirrhotic patients
Leber, Balazs *et al.,* 2021 [22]	85 chronic hepatitis C patients, including 56 patients with cirrhosis (mean age 58; 34 female, 51 male); 21 healthy controls	Hepatitis C; Child-Pugh A (42 patients), Child-Pugh B (12 patients), Child-Pugh C (2 patients)	No active bacterial infection	Not applicable	The Phagotest kit (Glycotope) with whole blood	Phagocytic capacity of *E.coli* is reduced and a percentage of non-phagocytic neutrophils is increased in cirrhotic patients

Laffi *et al.*, 1993 [10]	10 cirrhotic patients (5 female, 5 male; age 42-65); 10 healthy controls	Child-Pugh B (4 patients), Child-Pugh C (6 patients); hepatitis C (8 patients), hepatitis B (1 patient), hepatitis B and C (1 patient)	No infections	Ficoll-Hypaque gradient centrifugation, Böyum method	Light microscopy	No phagocytosis defect of *C. albicans* is shown in neutrophils of cirrhotic patients
Makkar *et al.*, 2020 [23]	40 patients with AD of cirrhosis (age 46.3; 35 male); 10 healthy controls	10 patients of each grade 0, 1, 2 and ACLF; alcohol (30 patients), hepatitis B+alcohol (2 patients), hepaitits C+alcohol (2 patients), autoimmune (4 patients), NASH (2 patients), others (1 patient)	No active infection	Not applicable	Cayman's Phagocytosis Assay Kit with whole blood	In ACLF impaired phagocytosis of latex beads has been reported and associated with 90-day survival
Tritto *et al.*, 2011 [24]	108 patients with liver cirrhosis (79 male, 29 female; mean age 57.5). For whole blood experiments: 8 patients (mean age 48; 6 male, 2 female) and 5 healthy controls	Stable cirrhosis (Child-Pugh A or B 58 patients, Child-Pugh C 50 patients); alcohol 41 patients, HBV/HCV 45 patients, other 22 patients. For whole blood experiments: Child-Pugh A 5 patients, Child-Pugh B – 3 patients; alcohol 8 patients	No bacterial infections at inclusion	Polymorphprep	The Phagotest kit (Orpegen Pharma) - healthy donor neutrophils incubated with patients' or healthy donor plasma or in whole blood	Decreased phagocytic capacity of normal neutrophils incubated with patient's plasma compared to control plasma. It did not correlate with cirrhosis etiology or 3-month mortality and morbidity, but correlated with disease severity. Phagocytic capacity of peripheral blood neutrophils from patients with compensated liver cirrhosis was not different compared to healthy controls.
Campbell *et al.*, 1981 [5]	44 patients with alcohol associated liver disease (22 male, 22 female; age 17-81), including 31 patients with cirrhosis	Alcohol (13 patients), chronic active hepatitis (10 patients), primary biliary cirrhosis (8 patients), cryptogenic cirrhosis (13 patients)	-	Dextran sedimentation	Neutrophils are incubated with *C.albicans* with added [^{3}H]uridine (which is incorporated only by viable extracellular organisms); phagocytosis index is calculated based on the radioactivity measurements	Cirrhotic neutrophil phagocytosis of *C.albicans* opsonized with autologous plasma was not different compared to phagocytosis of *C.albicans* opsonized with healthy donor plasma, except for patients with chronic active hepatitis
**Killing capacity**						
Rajkovic *et al.*, 1986 [13]	40 patients with alcohol associated liver disease (39-66	Alcohol	No active bacterial	Dextran sedimentation and Ficoll-	Neutrophils incubated with bacteria in 96	Intracellular killing of *S. aureus* and *E.*

	years), all had cirrhosis, including 18 patients with superimposed acute alcoholic hepatitis; 20 healthy controls		infection at inclusion	Hypaque gradient centrifugation	well plate with added [^{3}H]uridine; killing index is calculated based on the radioactivity measurements	*coli* is impaired in alcohol related cirrhosis
Boussif *et al.*, 2016 [25]	32 patients with alcohol associated cirrhosis (mean age 57.4; 6 female); Alcohol consumption stopped for at least 3 days	Alcohol; Child-Pugh B or C;	No bacterial infection within one week; 18 patients with previous bacterial infections	Dextran sedimentation and Ficoll-Hypaque gradient centrifugation	The number of viable bacteria after incubation with neutrophils is calculated	Impaired *E.coli* killing upon stimulation with fMLF in neutrophils from cirrhotic patients
De Fernandez *et al.,* 1987 [12]	57 patients with liver disease	19 males and 3 females with alcohol associated liver disease (mean age 54) including 14 patients with cirrhosis; 2 males and 19 females with PBC (mean age 60.4); 5 male and 9 female patients with chronic active hepatitis (mean age 65) of mainly autoimmune etiology; no significant alcohol consumption within 10 days before study	All patients clinically free from infections at the time of study	Dextran sedimentation	Intracellular killing capacity of *S.aureus* calculated as viable intracellular *S.aureus* count divided by the difference between total viable *S.aureus* after incubation with neutrophils and viable extracellular *S.aureus* after incubation with neutrophils	No differences in neutrophil intracellular killing capacity of *S. aureus* in alcohol associated cirrhosis and PBC, despite decreased total bacterial killing, which the authors explain by decreased percentage of phagocytic neutrophils
Knooihuizen *et al.,* 2021[11]	21 patients with cirrhosis, 23 healthy controls	Alcohol (57%), non-alcoholic fatty liver disease (29%), hepatitis C virus (14%), other (19%); 6 patients with AD, 15 patients had ACLF	4 patients had spontaneous bacterial peritonitis	EasySep Direct Human Neutrophil Isolation Kit	Neutrophils are incubated with *Candida albicans* in 96 well plates; percentage of remaining live pathogen is measured with PrestoBlue Cell Viability Reagent	Impaired killing capacity of *Candida albicans* by neutrophils from cirrhotic patients
Laffi *et al.,* 1993 [10]	10 cirrhotic patients (5 female, 5 male; age 42-65); 10 healthy controls	Child-Pugh B (4 patients), Child-Pugh C (6 patients); hepatitis C (8 patients), hepatitis B (1 patient), hepatitis B and C (1 patient)	No infections	Ficoll-Hypaque gradient	Killing index is determined as the ratio of killed *C.albicans* to total input number; dead *C.albicans* is identified by staining with methylene blue after incubation with neutrophils	No *C.albicans* killing capacity defect in neutrophils from cirrhotic patients
Campbell *et al.,* 1981 [5]	44 patients with alcohol associated liver disease (22 male, 22 female; age 17-81), including 31	Alcohol (13 patients), chronic active hepatitis (10 patients), primary biliary cirrhosis (8		Dextran sedimentation	Neutrophils are incubated with *C.albicans* with added	Cirrhotic neutrophil killing of *C.albicans* opsonized with autologous plasma was not different

	patients with cirrhosis	patients), cryptogenic cirrhosis (13 patients)			[3H]uridine (which is incorporated only by viable extracellular organisms); killing activity is calculated based on the radioactivity measurements	compared to killing of *C.albicans* opsonized with healthy donor plasma
**ROS production**						
Horvath *et al.*, 2016 [18]	80 patients with cirrhosis (mean age probiotics group 60, placebo group 56; 58 male, 22 female); 51 healthy controls; no alcohol abuse within 2 weeks before the study	Alcohol (44 patients), hepatitis C (13 patients), other (22 patients); Child-Pugh A (61 patients), Child-Pugh B+C (19 patients)	No active infection at screening	Not applicable	The Phagoburst kit (Glycotope) with whole blood	Elevated percentage of neutrophils with basal ROS production and in response to fMLF, but unchanged in response to *E.coli* in cirrhosis
Mookerjee *et al.*, 2007 [14]	63 patients with AD of alcohol associated cirrhosis; 20 healthy controls	Alcohol; cirrhosis and superimposed inflammatory alcoholic hepatitis	No clinical or microbiological evidence of infection	Not applicable	The Phagoburst kit (Orpegen Pharma) with whole blood	Elevated percentage of neutrophils with basal ROS production and in response to fMLF, but unchanged in response to *E.coli* in cirrhosis
Leber, Balazs *et al.*, 2021 [22]	85 chronic hepatitis C patients, including 56 patients with cirrhosis (mean age 58; 34 female, 51 male); 21 healthy controls	Hepatitis C; Child-Pugh A (42 patients), Child-Pugh B (12 patients), Child-Pugh C (2 patients)	No active bacterial infection	Not applicable	The Phagoburst kit (Glycotope) with whole blood	Elevated percentage of neutrophils with basal ROS production in cirrhosis, but unchanged ROS production in response to fMLF and *E.coli*
Bruns *et al.*, 2011 [26]	84 patients with liver cirrhosis (male 77%, age 37-82); no active alcohol consumers; 29 healthy controls	Alcohol associated cirrhosis (80%), 4 patients with viral cirrhosis, 6 patients with cryptogenic cirrhosis, 7 patients with other etiologies of cirrhosis; Child-Pugh A (7 patiens), Child-Pugh B (43 patients), Child-Pugh C (34 patients)	45 patients had signs of infection at the inclusion	Not applicable	The Phagoburst kit (Glycotope) with whole blood	Elevated percentage of neutrophils with basal ROS production, as well as basal intracellular ROS produced in cirrhosis; no difference between infected and not infected patients, but association with disease severity. No difference in percentage of neutrophils producing ROS in response to fMLF between cirrhosis and healthy, however, intracellular ROS upon fMLF stimulation is higher in cirrhosis – no correlation with infections. Percentage of neutrophils produced ROS in response to *E.coli* in

						cirrhosis is unchanged compared to healthy, the amount of intracellular ROS in response to *E.coli* is increased in cirrhosis without infection.
Makkar *et al.,* 2020 [23]	40 patients with AD of cirrhosis (age 46.3; 35 male); 10 healthy controls	10 patients of each grade 0, 1, 2 and ACLF; alcohol (30 patients), hepatitis B+alcohol (2), hepatitis C+alcohol (2 patients), autoimmune (4 patients), NASH (2), others (1 patient)	No active infection	Not applicable	Oxidation of dihydrorhodamine−123 to rhodamine measured by flow cytometry	Elevated percentage of basal ROS production in neutrophils from patients with AD and ACLF
Balazs *et al.,* 2022 [21]	109 cirrhotic patients (mean age alcohol 56, hepatitis C 60, other 55; alcohol 42 male, 12 female, hepatitis C 22 male, 10 female, other 13 male, 10 female); 21 healthy controls	Alcohol (54 patients), hepatitis C (32 patients), other (23 patients); Child-Pugh A (79 patients), Child-Pugh B+C (30 patients)	No active bacterial infection	Not applicable	The Phagoburst kit (Celonic) with whole blood	Elevated percentage of neutrophils with basal ROS production and ROS production in response to fMLF, but decreased ROS production in response to *E.coli* in cirrhosis. Intracellular ROS production is unchanged
Huang *et al.,* 2016 [16]	48 healthy volunteers and 100 cirrhotic patients (77 male, 23 female; mean age: 55 ± 13 years)	26 patients with Child-Pugh A, 26 patients with Child-Pugh B, 48 patients in Child-Pugh C; etiologies: alcohol, hepatitis B and hepatitis C	-	Not applicable	The Phagoburst kit (Orpegen Pharma) with whole blood	Significantly higher level of intracellular basal ROS production in neutrophils from cirrhotic patients of Child-Pugh C
Rolas *et al.,* 2013 [27]	17 patients with cirrhosis (age mean 56; 8 female, 9 male);	Child-Pugh B (4 patients) or C (13) patients; alcohol associated	No current bacterial infections	Ficoll-Hypaque gradient	Cytochrome C reduction assay with isolated neutrophils and chemiluminescence in whole blood in presence of luminol	Reduced intracellular basal ROS production and ROS production in response to fMLF; decreased extracellular superoxide release in response to fMLF
Rajkovic *et al.,* 1986 [13]	40 patients with alcohol associated liver disease (39-66 years), all had cirrhosis, including 18 patients with superimposed acute alcoholic hepatitis; 20 healthy controls	Alcohol	No active bacterial infection at inclusion	Dextran sedimentation and Ficoll-Hypaque gradient centrifugation	Cytochrome C reduction assay to measure superoxide, with addition of phenol red to measure peroxide	Extracellular basal superoxide production is unchanged, but reduced in response to zymosan in cirrhotic patients; extracellular hydrogen peroxide levels produced by neutrophils in response to zymosan are not different or higher in cirrhotic

						patients compared to healthy controls
Laffi *et al.*, 1993 [10]	10 cirrhotic patients (5 female, 5 male; age 42-65); 10 healthy controls	Child-Pugh B (4 patients), Child-Pugh C (6 patients); hepatitis C (8 patients), hepatitis B (1 patient), hepatitis B and C (1 patient)	No infections	Ficoll-Hypaque gradient centrifugation, Böyum method	Cytochrome C reduction assay	Extracellular basal superoxide production elevated in cirrhosis, extracellular superoxide production in response to zymosan reduced in cirrhosis
Stadlbauer *et al.*, 2008 [15]	20 patients with alcohol associated cirrhosis (18-75 years; 15 male, 5 female) including 8 cirrhotic patients as disease control; 13 healthy controls	Alcohol	No clinical or microbiological evidence of infection	Not applicable	The Phagoburst kit (Orpegen Pharma) with whole blood	Increased percentage of neutrophils producing ROS in response to fMLF; slightly increased basal ROS production in disease controls
Garfia *et al.*, 2004 [28]	98 patients with liver cirrhosis (66 male, 32 female; age 39-76); 46 healthy controls	Alcohol (45 patients), hepatitis C (17 patients), hepatitis B (7 patients), mixed alcohol and viral (24 patients), autoimmune (1 patient), cryptogenic (4 patients); Child-Pugh A (20 patients), Child-Pugh B (20 patients), Child-Pugh C (58 patients)	24 patients had previous episodes of spontaneous bacterial peritonitis; patients with recent history of bacterial infections excluded	Lymphoprep centrifugation	Cytochrome C reduction assay	Extracellular superoxide release in response to fMLF, TNF-alpha and zymosan decreased in patients with cirrhosis; no association with etiology of cirrhosis, but with severity of liver disease
Rolas *et al.*, 2018 [29]	44 patients with cirrhosis (mean age 59.1 years, 6 female); Alcohol consumption stopped for at least 1 week before the study	Child-Pugh B (14 patients) or C (30 patients); alcohol	16 patients with previous bacterial infections, but no current bacterial infections	Ficoll-Hypaque gradient	Cytochrome C reduction assay	Extracellular superoxide production decreased in response to fMLF in patients with cirrhosis
Weiss *et al.*, 2021 [30]	31 patients with cirrhosis (age 52-64; 23 male)	Advanced cirrhosis (7 patients), acute decompensation of cirrhosis (7 patients), acute-on-chronic liver failure (17 patients); alcohol (21 patients), non-alcohol steatohepatitis (6 patients), chronic hepatits C (3 patients), chronic hepatitis B (1 patient),	No bacterial infections within 1 week before inclusion	Ficoll-Hypaque gradient	Cytochrome C reduction assay	Decrease in superoxide release in response to fMLF significantly more pronounced in patients with ACLF compared to patients with advanced cirrhosis
Taylor *et al.*, 2014 [19]	62 patients with cirrhosis (age 39-61; 17 female)	Stable cirrhosis (49 patients), ACLF (13 patients); etiologies: alcohol, hepatitis C, autoimmune liver disease, other	7 patients with active infection in ACLF group	Not applicable	The Bursttest kit (Orpegen Pharma) with whole blood	Basal ROS production elevated in ACLF compared to healthy controls, but not in stable cirrhosis and not associated with the severity of disease; ROS production in

						response to *E.coli* unchanged in all cirrhotic patients compared to healthy controls and not associated with either severity or etiology of disease; no difference in ROS production in active and abstinent drinkers
Masini *et al.,* 1995 [31]	12 cirrhotic patients with ascites; 10 healthy controls	-	-	Lymphoprep gradient	Superoxide production assessed with cytochrome c reduction assay; nitric oxide production evaluated using neutrophil capacity to inhibit thrombin-induced platelet aggregation and increase cGMP content in thrombin induced platelets co-incubated with neutrophils	Reduced extracellular superoxide production in response to zymosan in patients with cirrhosis; increased nitric oxide production in response to opsonized zymosan in neutrophils from cirrhotic patients
Wu *et al.,* 2021 [20]	18 patients with hepatitis B virus related ACLF, 14 patients with compensated liver cirrhosis, 13 healthy controls	Hepatitis B virus ACLF or compensated liver cirrhosis	No active bacterial infection	Not applicable	The Phagoburst test (Glycotope)	Elevated number of neutrophils with basal ROS production in both compensated cirrhosis and ACLF, decreased intracellular basal ROS production in neutrophils from patients with compensated cirrhosis
Tritto *et al.,* 2011 [24]	108 patients with liver cirrhosis (79 male, 29 female; mean age 57.5). For whole blood experiments: 8 patients (mean age	Stable cirrhosis (Child-Pugh A or B 58 patients, Child-Pugh C 50 patients); alcohol 41 patients, HBV/HCV 45 patients, other 22 patients.	No bacterial infection at inclusion	Polymorphprep	The Phagoburst kit (Orpegen Pharma) - healthy donor neutrophils incubated with patients' or	Basal ROS production in healthy donor neutrophils incubated with patients' plasma was not

	48; 6 male, 2 female) and 5 healthy controls	For whole blood experiments: Child-Pugh A 5 patients, Child-Pugh B – 3 patients; alcohol 8 patients			healthy donor plasma or in whole blood	significantly different from neutrophils incubated with healthy controls' plasma. No difference in basal ROS production and ROS production in response to E.coli in neutrophils from cirrhotic patients compared to healthy controls.
**Degranulation**						
Rajkovic *et al.,* 1986 [13]	40 patients with alcohol associated liver disease (39-66 years), all had cirrhosis, including 18 patients with superimposed acute alcoholic hepatitis; 20 healthy controls	Alcohol	No active bacterial infection at inclusion	Dextran sedimentation and Ficoll-Hypaque gradient centrifugation	Incubation of neutrophils with Cytohalasin B and zymosan and then measurements of enzymes in supernatants	Enzyme intracellular contents (lysozyme, myeloperoxidase (MPO)) and their release from neutrophil granules upon stimulation with zymosan are reduced in neutrophils from cirrhotic patients; however, authors claim that the release reduction is not dependent on the reduction of the enzymes level inside the granules
Boussif *et al.,* 2016 [25]	32 patients with alcohol associated cirrhosis (mean age 57.4; 6 female); Alcohol consumption stopped for at least 3 days	Alcohol; Child-Pugh B or C;	No bacterial infection within one week; 18 patients with previous bacterial infections	Dextran sedimentation and Ficoll-Hypaque gradient centrifugation	Method is based on the horseradish peroxidase-catalysed oxidation of o-dianisidine by H2O2	Intracellular content of MPO is not altered in neutrophils from cirrhotic patients, but its extracellular release in response to fMLF is decreased; MPO activity unchanged
Garfia *et al.,* 2004 [28]	98 patients with liver cirrhosis (66 male, 32 female; age 39-76); 46 healthy controls	Alcohol (45 patients), hepatitis C (17 patients), hepatitis B (7 patients), mixed alcohol and viral (24 patients), autoimmune (1 patient), cryptogenic (4 patients); Child-Pugh A (20 patients), Child-Pugh B (20 patients), Child-Pugh C (58 patients)	24 patients with previous episodes of spontaneous bacterial peritonitis; patients with recent history of bacterial infections excluded from the study	Lymphoprep centrifugation	Absorbance measured in cell supernatant mixed with tetrametilbenzidine, hydrogen peroxide and sodium phosphate buffer	Decreased MPO activity in cirrhosis

Tranah *et al.*, 2017 [32]	29 patients with alcohol associated cirrhosis (age 45-59, 19 male, 10 female); 15 actively drinking patients, 14 patients abstinent; 12 healthy controls	Alcohol; Child-Pugh score 7-12	No evidence of infection at inclusion	Not applicable	Whole blood incubated with either *E.coli* or fMLF, then stained with fluorescent antibodies (CD66b, CD63, CD11b, MPO, CD16), analysed by flow cytometry. For intracellular granular phenotype a permeabilizatio n step is added	In patients with alcohol associated cirrhosis increased mobilization to the cell surface of the primary neutrophil granules is observed. Active alcohol consumption decreases the hyper responsiveness of cirrhotic neutrophils.
Wu *et al.*, 2021 [20]	18 patients with hepatitis B virus related ACLF, 14 patients with compensated liver cirrhosis, 13 healthy controls	Hepatitis B virus ACLF or compensated liver cirrhosis	No active bacterial infection	Not applicable	Whole blood stained with anti-MPO-FITC and analysed with flow cytometry	Decreased number of neutrophils producing MPO and intracellular MPO in neutrophils from patients with compensated cirrhosis, but not with ACLF
**NETs formation**						
Agraz-Cibrian *et al.*, 2016 [33]	60 patients with cirrhosis; 20 healthy controls	Alcohol (42 patients), hepatitis C (6 patients), other (12 patients); compensated cirrhosis (20 patients), decompensate d cirrhosis with ascites (20 patients), decompensate d cirrhosis with spontaneous bacterial peritonitis (20 patients)	20 patients with spontaneous bacterial peritonitis	Ficoll Histopaque 1119/1077 density gradient	Immunofluoresc ence; fluorimetry (DNA/NETs)	Decrease in NETs formation in response to phorbol-12-myristat-13-acetat (PMA) in patients with liver cirrhosis complicated with spontaneous bacterial peritonitis compared to healthy controls
Agraz-Cibrian *et al.*, 2018 [34]	40 patients with liver cirrhosis (27 male, 13 female; age group with ascites 49.56, group with ascites and spontaneous bacterial peritonitis 55.6); 20 healthy controls	Alcohol (34 patients), hepatitis C (4 patients), other (2 patients); decompensate d cirrhosis with ascites (20 patients), decompensate d cirrhosis with ascites and spontaneous bacterial peritonitis (20 patients)	20 patients with spontaneous bacterial peritonitis	Ficoll Histopaque 1119/1077 density gradient	Immunofluoresc ence	Decrease in NETs formation in response to PMA in patients with liver cirrhosis complicated with spontaneous bacterial peritonitis compared to healthy controls
Sehgal *et al.*, 2022 [17]	70 patients with decompensated cirrhosis (63 male, 7 female; age 22-60); 15 healthy controls	Decompensate d cirrhosis; alcohol predominant etiology (70%)	40 patients with sepsis	Polymorphoprep	Immunofluoresc ence; fluorimetry	Plasma of patients with decompensated cirrhosis induces NETs formation in isolated neutrophils from healthy controls
Zenlander *et al.*, 2021 [35]	95 patients with cirrhosis (mean age 63.4; 62 male), 82	Alcohol (34 patients), NASH (19	-	Not applicable	ELISA (H3Cit-DNA, MPO-	Both H3Cit-DNA and MPO-DNA levels

	patients with hepatocellular carcinoma; 50 healthy controls	patients), viral hepatitis (20 patients), other (22 patients); severity in cirrhosis group: Child-Pugh A (47 patients), Child-Pugh B (34 patients), Child-Pugh C (14 patients)			DNA) with plasma	significantly elevated in patients with cirrhosis compared to healthy controls, indicating increased NETs formation in this group; associated with disease severity
Blasi *et al.,* 2019 [36]	52 patients with AD of cirrhosis (mean age 58, 29 male), 57 patients with ACLF (mean age 59, 40 male); 40 healthy controls	Alcohol (AD: 32, ACLF 33), viral (ACLF 11), NASH (AD 10, ACLF 6), biliary (AD 3, ACLF 2), other (AD 7, ACLF 5); Child-Pugh (AD 7-10, ACLF 8-12)	7 patients with AD and 26 patients with ACLF had infections	Not applicable	Quant-iT PicoGreen double strand DNA assay kit (concentration of cell-free DNA in plasma); ELISA (MPO-DNA in plasma)	Cell-free DNA and MPO-DNA complexes in plasma are elevated in both AD and ACLF compared to healthy controls
Wu *et al.,* 2021 [20]	18 patients with hepatitis B virus related ACLF, 14 patients with compensated liver cirrhosis, 13 healthy controls	Hepatitis B virus ACLF or compensated liver cirrhosis	No active bacterial infection	Not applicable	Whole blood stained with FITC-MPO antibodies and SYTOX red and analysed by flow cytometry or imaging flow cytometry	NETs formation in response to *E.coli*, fMLF and PMA as well as spontaneous NETs formation is elevated in patients with compensated cirrhosis and ACLF
**Apoptosis and viability**						
Kusaba *et al.,* 1998 [37]	10 cirrhotic patients (3 men and 7 women; 42-75 years old); 10 healthy controls	Hepatitis C (8 patients), hepatitis B (2 patients);	-	Percoll gradient	Viability is assessed with trypan blue; apoptosis is assessed with light microscopy and TUNEL assay	Increased apoptosis rate and decreased viability of neutrophils isolated from neutropenic patients with viral liver cirrhosis 24 hours after isolation from whole blood
Ramirez *et al.,* 2004 [38]	17 patients with cirrhosis and ascites (mean age 61.8; 10 male, 7 female); 13 patients with compensated chronic liver disease	Hepatitis C (10 patients), hepatitis B (1 patient), alcohol (5 patients), other (1 patient)	-	Ficoll-Hypaque gradient centrifugation, Boyum method	Viability is determined with MTT assay and trypan blue; apoptosis is assessed with light microscopy and TACs apoptotic DNA laddering kit	Decreased viability from cirrhotic patients; increased apoptosis of neutrophils in patients with decompensated cirrhosis

Supplementary references

1. Rajkovic, I. A.; Yousif-Kadaru, A. G.; Wyke, R. J.; Williams, R., Polymorphonuclear leucocyte locomotion and aggregation in patients with alcoholic liver disease. *Clin Exp Immunol* **1984,** 58, (3), 654-62.
2. Fiuza, C.; Salcedo, M.; Clemente, G.; Tellado, J. M., In vivo neutrophil dysfunction in cirrhotic patients with advanced liver disease. *J Infect Dis* **2000,** 182, (2), 526-33.
3. Onishi, S.; Saibara, T.; Maeda, T.; Yamamoto, Y.; Ito, K., Serum inhibition of complement derived leukocyte chemotaxis and levels of immunoglobulin A subclass in alcoholic liver disease. *Gastroenterol Jpn* **1989,** 24, (3), 284-9.
4. Vanepps, D. E.; Strickland, R. G.; Williams, R. C., Inhibitors of Leukocyte Chemotaxis in Alcoholic Liver-Disease. *American Journal of Medicine* **1975,** 59, (2), 200-207.
5. Campbell, A. C.; Dronfield, M. W.; Toghill, P. J.; Reeves, W. G., Neutrophil function in chronic liver disease. *Clin Exp Immunol* **1981,** 45, (1), 81-9.
6. Fiuza, C.; Salcedo, M.; Clemente, G.; Tellado, J. M., Granulocyte colony-stimulating factor improves deficient in vitro neutrophil transendothelial migration in patients with advanced liver disease. *Clinical and Diagnostic Laboratory Immunology* **2002,** 9, (2), 433-439.
7. Artru, F.; Bou Saleh, M.; Maggiotto, F.; Lassailly, G.; Ningarhari, M.; Demaret, J.; Ntandja-Wandji, L. C.; Pais de Barros, J. P.; Labreuche, J.; Drumez, E.; Helou, D. G.; Dharancy, S.; Gantier, E.; Perianin, A.; Chollet-Martin, S.; Bataller, R.; Mathurin, P.; Dubuquoy, L.; Louvet, A., IL-33/ST2 pathway regulates neutrophil migration and predicts outcome in patients with severe alcoholic hepatitis. *J Hepatol* **2020,** 72, (6), 1052-1061.
8. Claria, J.; Titos, E.; Jimenez, W.; Ros, J.; Gines, P.; Arroyo, V.; Rivera, F.; Rodes, J., Altered biosynthesis of leukotrienes and lipoxins and host defense disorders in patients with cirrhosis and ascites. *Gastroenterology* **1998,** 115, (1), 147-56.
9. Langer, M. M.; Sichelschmidt, S.; Bauschen, A.; Bornemann, L.; Guckenbiehl, S.; Gunzer, M.; Lange, C. M., Pathological neutrophil migration predicts adverse outcomes in hospitalized patients with liver cirrhosis. *Liver Int* **2023,** 43, (4), 896-905.
10. Laffi, G.; Carloni, V.; Baldi, E.; Rossi, M. E.; Azzari, C.; Gresele, P.; Marra, F.; Gentilini, P., Impaired superoxide anion, platelet-activating factor, and leukotriene B4 synthesis by neutrophils in cirrhosis. *Gastroenterology* **1993,** 105, (1), 170-7.
11. Knooihuizen, S. A. I.; Alexander, N. J.; Hopke, A.; Barros, N.; Viens, A.; Scherer, A.; Atallah, N. J.; Dagher, Z.; Irimia, D.; Chung, R. T.; Mansour, M. K., Loss of Coordinated Neutrophil Responses to the Human Fungal Pathogen, Candida albicans, in Patients With Cirrhosis. *Hepatol Commun* **2021,** 5, (3), 502-515.
12. De Fernandez, M. A.; Clark, A.; Triger, D. R., Neutrophil phagocytic and bactericidal function in primary biliary cirrhosis and other chronic liver diseases. *Clin Exp Immunol* **1987,** 67, (3), 655-61.
13. Rajkovic, I. A.; Williams, R., Abnormalities of neutrophil phagocytosis, intracellular killing and metabolic activity in alcoholic cirrhosis and hepatitis. *Hepatology* **1986,** 6, (2), 252-62.
14. Mookerjee, R. P.; Stadlbauer, V.; Lidder, S.; Wright, G. A.; Hodges, S. J.; Davies, N. A.; Jalan, R., Neutrophil dysfunction in alcoholic hepatitis superimposed on cirrhosis is reversible and predicts the outcome. *Hepatology* **2007,** 46, (3), 831-40.
15. Stadlbauer, V.; Mookerjee, R. P.; Hodges, S.; Wright, G. A.; Davies, N. A.; Jalan, R., Effect of probiotic treatment on deranged neutrophil function and cytokine responses in patients with compensated alcoholic cirrhosis. *J Hepatol* **2008,** 48, (6), 945-51.
16. Huang, C. H.; Jeng, W. J.; Ho, Y. P.; Teng, W.; Hsieh, Y. C.; Chen, W. T.; Chen, Y. C.; Lin, H. H.; Sheen, I. S.; Lin, C. Y., Increased EMR2 expression on neutrophils correlates with disease severity and predicts overall mortality in cirrhotic patients. *Sci Rep* **2016,** 6, 38250.
17. Sehgal, R.; Kaur, N.; Maiwall, R.; Ramakrishna, G.; Maras, J. S.; Trehanpati, N., Plasma Proteomic Analysis Identified Proteins Associated with Faulty Neutrophils Functionality in Decompensated Cirrhosis Patients with Sepsis. *Cells* **2022,** 11, (11).
18. Horvath, A.; Leber, B.; Schmerboeck, B.; Tawdrous, M.; Zettel, G.; Hartl, A.; Madl, T.; Stryeck, S.; Fuchs, D.; Lemesch, S.; Douschan, P.; Krones, E.; Spindelboeck, W.; Durchschein, F.; Rainer, F.; Zollner, G.; Stauber, R. E.; Fickert, P.; Stiegler, P.; Stadlbauer, V., Randomised clinical trial: the effects of a multispecies probiotic vs. placebo on innate immune function,

bacterial translocation and gut permeability in patients with cirrhosis. *Aliment Pharmacol Ther* **2016,** 44, (9), 926-935.
19. Taylor, N. J.; Vijay, G. K. M.; Abeles, R. D.; Auzinger, G.; Bernal, W.; Ma, Y.; Wendon, J. A.; Shawcross, D. L., The severity of circulating neutrophil dysfunction in patients with cirrhosis is associated with 90-day and 1-year mortality. *Alimentary Pharmacology & Therapeutics* **2014,** 40, (6), 705-715.
20. Wu, W.; Sun, S.; Wang, Y.; Zhao, R.; Ren, H.; Li, Z.; Zhao, H.; Zhang, Y.; Sheng, J.; Chen, Z.; Shi, Y., Circulating Neutrophil Dysfunction in HBV-Related Acute-on-Chronic Liver Failure. *Front Immunol* **2021,** 12, 620365.
21. Balazs, I.; Horvath, A.; Leber, B.; Feldbacher, N.; Sattler, W.; Rainer, F.; Fauler, G.; Vermeren, S.; Stadlbauer, V., Serum bile acids in liver cirrhosis promote neutrophil dysfunction. *Clin Transl Med* **2022,** 12, (2), e735.
22. Leber, B.; Balazs, I.; Horvath, A.; Posch, A.; Streit, A.; Spindelbock, W.; Feldbacher, N.; Stiegler, P.; Stauber, R. E.; Rechberger, G. N.; Kollroser, M.; Sattler, W.; Nusshold, C.; Stadlbauer, V., Direct acting antiviral therapy rescues neutrophil dysfunction and reduces hemolysis in hepatitis C infection. *Transl Res* **2021,** 232, 103-114.
23. Makkar, K.; Tomer, S.; Verma, N.; Rathi, S.; Arora, S. K.; Taneja, S.; Duseja, A.; Chawla, Y. K.; Dhiman, R. K., Neutrophil dysfunction predicts 90-day survival in patients with acute on chronic liver failure: A longitudinal case-control study. *JGH Open* **2020,** 4, (4), 595-602.
24. Tritto, G.; Bechlis, Z.; Stadlbauer, V.; Davies, N.; Frances, R.; Shah, N.; Mookerjee, R. P.; Such, J.; Jalan, R., Evidence of neutrophil functional defect despite inflammation in stable cirrhosis. *J Hepatol* **2011,** 55, (3), 574-581.
25. Boussif, A.; Rolas, L.; Weiss, E.; Bouriche, H.; Moreau, R.; Perianin, A., Impaired intracellular signaling, myeloperoxidase release and bactericidal activity of neutrophils from patients with alcoholic cirrhosis. *Journal of Hepatology* **2016,** 64, (5), 1041-1048.
26. Bruns, T.; Peter, J.; Hagel, S.; Herrmann, A.; Stallmach, A., The augmented neutrophil respiratory burst in response to Escherichia coli is reduced in liver cirrhosis during infection. *Clin Exp Immunol* **2011,** 164, (3), 346-56.
27. Rolas, L.; Makhezer, N.; Hadjoudj, S.; El-Benna, J.; Djerdjouri, B.; Elkrief, L.; Moreau, R.; Perianin, A., Inhibition of mammalian target of rapamycin aggravates the respiratory burst defect of neutrophils from decompensated patients with cirrhosis. *Hepatology* **2013,** 57, (3), 1163-71.
28. Garfia, C.; Garcia-Ruiz, I.; Solis-Herruzo, J. A., Deficient phospholipase C activity in blood polimorphonuclear neutrophils from patients with liver cirrhosis. *Journal of Hepatology* **2004,** 40, (5), 749-756.
29. Rolas, L.; Boussif, A.; Weiss, E.; Letteron, P.; Haddad, O.; El-Benna, J.; Rautou, P. E.; Moreau, R.; Perianin, A., NADPH oxidase depletion in neutrophils from patients with cirrhosis and restoration via toll-like receptor 7/8 activation. *Gut* **2018,** 67, (8), 1505-1516.
30. Weiss, E.; de la Grange, P.; Defaye, M.; Lozano, J. J.; Aguilar, F.; Hegde, P.; Jolly, A.; Moga, L.; Sukriti, S.; Agarwal, B.; Gurm, H.; Tanguy, M.; Poisson, J.; Claria, J.; Abback, P. S.; Perianin, A.; Mehta, G.; Jalan, R.; Francoz, C.; Rautou, P. E.; Lotersztajn, S.; Arroyo, V.; Durand, F.; Moreau, R., Characterization of Blood Immune Cells in Patients With Decompensated Cirrhosis Including ACLF. *Front Immunol* **2020,** 11, 619039.
31. Masini, E.; Mugnai, L.; Foschi, M.; Laffi, G.; Gentilini, P.; Mannaioni, P. F., Changes in the production of nitric oxide and superoxide by inflammatory cells in liver cirrhosis. *Int Arch Allergy Immunol* **1995,** 107, (1-3), 197-8.
32. Tranah, T. H.; Vijay, G. K. M.; Ryan, J. M.; Abeles, R. D.; Middleton, P. K.; Shawcross, D. L., Dysfunctional neutrophil effector organelle mobilization and microbicidal protein release in alcohol-related cirrhosis. *Am J Physiol Gastrointest Liver Physiol* **2017,** 313, (3), G203-G211.
33. Agraz-Cibrian, J. M.; Segura-Ortega, J. E.; Delgado-Rizo, V.; Fafutis-Morris, M., Alterations in neutrophil extracellular traps is associated with the degree of decompensation of liver cirrhosis. *J Infect Dev Ctries* **2016,** 10, (5), 512-7.
34. Agraz-Cibrian, J. M.; Delgado-Rizo, V.; Segura-Ortega, J. E.; Maldonado-Gomez, H. A.; Zambrano-Zaragoza, J. F.; Duran-Avelar, M. J.; Vibanco-Perez, N.; Fafutis-Morris, M., Impaired neutrophil extracellular traps and inflammatory responses in the peritoneal fluid of patients with liver cirrhosis. *Scand J Immunol* **2018,** 88, (5), e12714.

35. Zenlander, R.; Havervall, S.; Magnusson, M.; Engstrand, J.; Agren, A.; Thalin, C.; Stal, P., Neutrophil extracellular traps in patients with liver cirrhosis and hepatocellular carcinoma. *Sci Rep* **2021,** 11, (1), 18025.
36. Blasi, A.; Patel, V. C.; Adelmeijer, J.; Azarian, S.; Aziz, F.; Fernandez, J.; Bernal, W.; Lisman, T., Plasma levels of circulating DNA are associated with outcome, but not with activation of coagulation in decompensated cirrhosis and ACLF. *JHEP Rep* **2019,** 1, (3), 179-187.
37. Kusaba, N.; Kumashiro, R.; Ogata, H.; Sata, M.; Tanikawa, K., In vitro study of neutrophil apoptosis in liver cirrhosis. *Intern Med* **1998,** 37, (1), 11-7.
38. Ramirez, M. J.; Titos, E.; Claria, J.; Navasa, M.; Fernandez, J.; Rodes, J., Increased apoptosis dependent on caspase-3 activity in polymorphonuclear leukocytes from patients with cirrhosis and ascites. *J Hepatol* **2004,** 41, (1), 44-8.
