# Supplementary material for: Circulating neutrophil anti-pathogen dysfunction in cirrhosis
Source: JHEP Rep. 2023 Aug 1;5(11):100871. doi: 10.1016/j.jhepr.2023.100871 (PMC10562928; doi:10.1016/j.jhepr.2023.100871)
Supplement: Multimedia component 3 [file mmc3.pdf]

Circulating neutrophil anti-pathogen dysfunction in cirrhosis

Irina Balazs,[1,2] Vanessa Stadlbauer[1,2,*]

Summary

Neutrophils are the largest population of leucocytes and are among the first cells of the innate immune system to fight against intruding pathogens. In patients with cirrhosis, neutrophils exhibit altered functionality, including changes in phagocytic ability, bacterial killing, chemotaxis, degranulation, reactive oxygen species production and NET (neutrophil extracellular trap) formation. This results in their inability to mount an adequate antibacterial response and protect the individual from infection. Prognosis and survival in patients with cirrhosis are greatly influenced by the development of infectious complications. Multidrug-resistant bacterial infections in patients with cirrhosis are currently a growing problem worldwide; therefore, alternative methods for the prevention and treatment of bacterial infections in cirrhosis are urgently needed. The prevention and treatment of neutrophil dysfunction could be a potential way to protect patients from bacterial infections. However, the reasons for changes in neutrophil function in cirrhosis are still not completely understood, which limits the development of efficient therapeutic strategies. Both cellular and serum factors have been proposed to contribute to the functional impairment of neutrophils. Herein, we review the current knowledge on features and proposed causes of neutrophil dysfunction in cirrhosis, with a focus on current knowledge gaps and limitations, as well as opportunities for future investigations in this field.

© 2023 The Authors. Published by Elsevier B.V. on behalf of European Association for the Study of the Liver (EASL). This is an open access article under the CC BY license (http://creativecommons.org/licenses/by/4.0/).

Keywords: neutrophils; cirrhosis; phagocytosis; ROS; chemotaxis; NETs

Received 18 March 2023; received in revised form 16 July 2023; accepted 22 July 2023; available online 1 August 2023

Introduction

Cirrhosis is a life-threatening chronic and progressive liver disease that develops primarily as a consequence of chronic hepatitis B or hepatitis C virus infections, or alcohol-related or non-alcoholic steatohepatitis, with other rarer causes including alpha-1-antitrypsin deficiency, haemochromatosis, primary sclerosis cholangitis, Wilson's disease, etc.[1,2] Being the 11th leading cause of death in the world, cirrhosis accounted for 2.2% of total deaths in 2016[3] and 2.4% of total deaths in 2017,[4] with an annually increasing mortality rate.[4,5] Compensated patients with cirrhosis have an ~5-fold increased mortality risk and decompensated patients a 10-fold increased mortality risk compared to the general population.[6] The prognosis and survival of patients with cirrhosis are greatly influenced by the development of bacterial infections, such as spontaneous bacterial peritonitis, urinary tract infections, and pneumonia, among others.[7–9]

Around one-third of hospitalized patients with cirrhosis suffer from bacterial infections, which leads to increased hospitalization time,[7] de-listing from the transplantation waiting list,[10] acute kidney injury[11] and, hence, a 4-fold increased mortality rate compared to non-infected patients with cirrhosis.[8] Of those with cirrhosis who develop bacterial infections, 30% die within 1 month and more than 60% die within 1 year after the infection.[8]

Infections in patients with cirrhosis are mostly caused by gram-negative bacteria (such as *Escherichia coli* and *Klebsiella pneumoniae*) of intestinal origin, but infections caused by gram-positive bacteria (such as *Staphylococcus aureus* and Enterococci) are on the rise, particularly in hospitalized patients.[12] Multidrug-resistant bacterial infections in cirrhosis are currently a growing problem worldwide; therefore, alternative methods for the prevention and treatment of bacterial infections in cirrhosis are urgently needed.[13,14]

An important reason for the increased susceptibility to bacterial infections in patients with cirrhosis is the progressive development of immune dysfunction.[15] Innate immune dysfunction, including impaired neutrophil functionality, is a predominant part of cirrhosis-associated immune dysfunction and, therefore, plays a major role in the development of bacterial infections in patients with cirrhosis.[16] The number of circulating neutrophils is frequently altered in cirrhosis. Neutropenia is often described in cirrhosis,[17] whereas neutrophilia has been reported for acute-on-chronic liver failure (ACLF).[18,19] The neutrophil-to-lymphocyte ratio is associated with liver-related mortality in patients with different stages of cirrhosis,[17,20] as well as in patients with ACLF.[19,21] Furthermore, an increased neutrophil-to-lymphocyte ratio in patients with cirrhosis positively correlates with the number of

[1]Department of Internal Medicine, Division of Gastroenterology and Hepatology, Medical University of Graz, Graz, Austria; [2]Center for Biomarker Research in Medicine (CBmed), Graz, Austria

* Corresponding author. Address: Department of Internal Medicine, Division of Gastroenterology and Hepatology, Medical University of Graz, Graz, Austria; Tel.: +43/316/385 82282.
E-mail address: vanessa.stadlbauer@medunigraz.at (V. Stadlbauer).

ELSEVIER

EASL The Home of Hepatology

circulating low-density neutrophils, which are considered pro-inflammatory.[20] The role of the neutrophil-to-lymphocyte ratio in cirrhosis has been extensively described elsewhere.[22] However, the number of neutrophils alone is not sufficient to understand the complex functional defects that are present in cirrhosis.

There is growing evidence of various defects in neutrophil function occurring during the course of cirrhosis; additionally, these defects have been associated with poor prognosis and mortality. Recent reviews by Irvine *et al.* 2019,[12] Bernsmeier *et al.* 2020,[16] and Albillos *et al.* 2022,[23] which address the problem of cirrhosis-associated immune dysfunction, provide only a succinct overview of changes in neutrophil function and their causes in cirrhosis, as they review the functional changes of other immune cells as well. Recent neutrophil-focused reviews by Xu *et al.* 2014,[24] Cho *et al.* 2020[25] and Liu *et al.* 2021[26] also provide only a relatively short overview of neutrophil dysfunction in cirrhosis, as they aim to provide an overview of neutrophil dysfunction in various liver diseases not limited to cirrhosis.

We therefore provide a detailed overview of various changes in neutrophil function specifically in cirrhosis, address the problem of inter-study discrepancies and point out differences in the methodology used to measure neutrophil function. Furthermore, we discuss the current theories explaining the nature of cirrhosis-associated neutrophil dysfunction, their pros and cons, and potential therapeutic targets and directions for future studies. We think it is time to critically review the knowledge gathered on the topic, identify and discuss current gaps, problems and conflicting findings, and thereby create a basis for future investigations.

Features of neutrophil dysfunction in cirrhosis

Neutrophils are the largest population of leucocytes and are among the first cells of the innate immune system to fight against intruding pathogens. Neutrophils express a range of surface G protein-coupled receptors, which enable them to sense chemoattractants (inflammatory mediators or pathogen-related molecules) and, consequently, migrate to the site of the intruding pathogen (mostly bacteria and fungi). This process of directed migration is called "chemotaxis". As soon as neutrophils reach the site of infection, they use one of their defence mechanisms to protect the host organism from the infectious agent. They can internalise the pathogen via a process called "phagocytosis" and kill it inside the phagolysosome using the microbiocidal contents of their intracellular granules (*e.g.* various proteases) and reactive oxygen species (ROS). These substances can also be released into the extracellular space by neutrophils to enable extracellular bacterial killing, but this can also cause tissue damage. Neutrophils produce different anti- and pro-inflammatory cytokines, which help regulate inflammatory as well as other physiological and pathophysiological processes. Furthermore, neutrophils have recently been described as being able to extrude their DNA, which is coated with histones and cytoplasmic and granular proteins (*e.g.* neutrophil elastase, myeloperoxidase, *etc.*), *i.e.* the so-called "neutrophil extracellular traps" (NETs), in order to catch, immobilize and kill infectious agents. Neutrophils are cells with a relatively short lifespan, which is extended in the case of infection. Neutrophils undergo apoptosis and then are phagocytosed by macrophages and dendritic cells to promote the resolution of inflammation (reviewed in 27–29).

All the aforementioned functions allow neutrophils to fight bacterial infections. In patients with cirrhosis, neutrophils exhibit altered functionality, including changes in phagocytic ability, bacterial killing, chemotaxis, degranulation, reactive oxygen species production and NET formation (Fig. 1, Table S1). This results in their inability to mount an adequate antibacterial response and protect the patient from infection.

Key points

- Neutrophil dysfunction in cirrhosis is associated with the development of bacterial infections and higher mortality.
- The functions of neutrophils that are impaired in cirrhosis include phagocytosis, ROS production, killing capacity, neutrophil extracellular trap formation and chemotaxis.
- Further features of neutrophil dysfunction in cirrhosis are still to be described and standardisation of methodology is needed.
- Intrinsic and serum defects have been proposed to cause neutrophil dysfunction, although neither can fully describe the extent of neutrophil dysfunction and findings are contradictory.
- Due to the lack of clinical studies with neutrophil function as a primary or secondary outcome, there is only a vague understanding of the best strategy to prevent and treat neutrophil dysfunction in cirrhosis.
- Further research is needed to develop a neutrophil function screening panel and biomarkers for clinical practice, and to further describe neutrophil dysfunction, its causes and consequences, and approaches for its prevention and treatment.

Chemotaxis

Neutrophil chemotaxis is reduced in patients with cirrhosis, either because of cellular defects or the impaired chemoattractant activity of cirrhotic serum.[30–35] Neutrophils from patients with alcohol-associated cirrhosis show reduced migration towards healthy serum compared to healthy donor neutrophils. Serum from patients with cirrhosis had less chemoattractant activity compared to healthy serum in the same study. No correlation with bacterial infections has been found;[30] however, another study reported lower neutrophil migration ability in patients with cirrhosis with previous bacterial infections compared to those without previous infection.[34] The decrease in serum chemoattractant activity might be explained by alterations in chemoattractant production found in patients with cirrhosis.[36] Interestingly, another study shows that serum chemotactic inhibitory activity (which shows how effectively cirrhotic serum inhibits the chemoattractant activity of healthy serum mixed with zymosan A) is higher in alcohol-associated cirrhosis compared to non-alcoholic cirrhosis.[32] Alcohol intake *per se* is a likely contributor to neutrophil dysfunction in alcohol-related cirrhosis as it has previously been shown to reduce neutrophil chemotaxis in rats,[37] transiently reduce neutrophil ROS production and phagocytosis in healthy volunteers,[38] and cause dysregulation of neutrophil function upon *in vitro* alcohol exposure.[39] Other studies also report the presence of serum chemotactic inhibitory activity in patients with cirrhosis.[33,35] Cirrhotic neutrophils show decreased transendothelial migration in response to N-formyl-met-leu-phe (fMLF), increased adhesion to endothelial cells (HMEC-1) and an altered expression of adhesion receptors, and higher CD11b and lower CD62L expression, which also indicates increased degranulation[40] and neutrophil ageing,[41] compared to healthy controls.[31] Decreased neutrophil migration towards interleukin (IL)-8 has been shown in cirrhosis, probably due to the decreased expression of CXC motif chemokine receptor 2 (which senses IL-8).[42] Also, decreased migration and adhesion of neutrophils isolated from patients with cirrhosis in response to leukotriene B4 has been

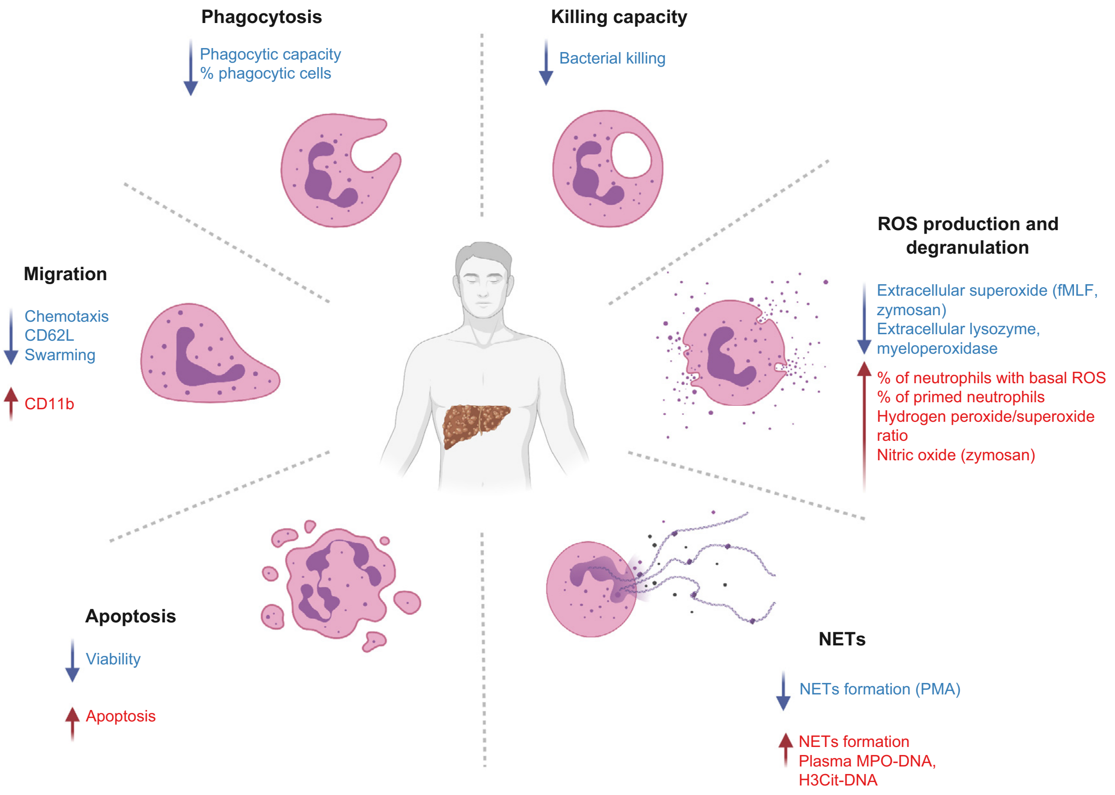

Fig. 1. Summary of neutrophil dysfunction features in cirrhosis. Findings consistent throughout the literature are shown. Created with Biorender.com.

reported.[36] Interestingly, the impairment in migration in the presence of fMLF is more pronounced in patients with acute decompensation and ACLF than in patients with compensated cirrhosis or healthy volunteers, which is less evident or absent (though with a tendency towards a decrease) when CXC motif ligand (CXCL)8 or CXCL1 is used as a chemoattractant. Furthermore, neutrophil migration patterns correlate with the incidence of adverse outcomes in this cohort of patients.[43] Both CXCL8 (IL-8) and CXCL1 have been shown to be highly elevated in the serum of patients with cirrhosis, which is even more pronounced in patients with ACLF.[44–46] This might initially preserve chemotaxis due to ligand excess or may also cause the homologous desensitisation of their receptors on neutrophils due to the prolonged excess of ligands, their downregulation and the subsequent decrease in neutrophil migration. The higher grade of impairment of neutrophil migration towards fMLF might also be explained by the different mechanisms involved, *e.g.* another FPR1 ligand that is highly abundant in cirrhosis, such as chenodeoxycholic acid (CDCA), might compete with fMLF for binding to the receptor and, as a result, inhibit neutrophil chemotaxis.[47] When casein is used as a chemoattractant, neutrophil chemotaxis is not affected in patients with cirrhosis. This indicates that defects in chemotaxis in cirrhosis are limited to certain pathways and depend on the chemoattractant used. The same study shows that the random migration of neutrophils is not affected in cirrhosis.[48]

Swarming

Only one study so far implies that swarming (coordinated neutrophil communication and recruitment) of neutrophils in order to control *Candida albicans* hyphae growth is impaired in patients with cirrhosis compared to healthy controls.[49]

Phagocytosis

Multiple studies have reported on the impaired phagocytic function of neutrophils from patients with cirrhosis.[34,50–58] Defective phagocytosis of *S. aureus* has been detected in isolated neutrophils in alcohol-associated cirrhosis[50,56] and primary biliary cholangitis (PBC),[56] as has defective phagocytosis of *E. coli* but to a lesser extent.[50] The decrease in neutrophil phagocytosis of *E. coli* is more pronounced in patients with cirrhosis who had previous bacterial infections.[34] Neutrophil phagocytic capacity (number of internalised bacteria per cell) of *E. coli* measured in whole blood or isolated neutrophils of patients with cirrhosis is impaired[51,52,57,59,60] or unchanged.[61,62] The percentage of phagocytic neutrophils is decreased in the blood of patients with cirrhosis[53,55,60,62,63] as well as in those with ACLF;[63] the degree of this dysfunction increases with increasing severity of cirrhosis and is higher in active drinkers compared to abstinent patients, but is not different between the aetiologies of cirrhosis (alcohol, HCV, autoimmune and other).[53] No defect in the phagocytosis of *C. albicans* was observed in patients with cirrhosis.[48] In ACLF, impaired phagocytosis of latex beads is reported and associated with 90-day survival.[58] It remains to be clarified whether these subtle observed differences in phagocytosis are dependent on the method used or if they represent true differences in pathogen phagocytosis.

Killing capacity

Bacterial killing is reduced in neutrophils from patients with cirrhosis, which means that even those active neutrophils which manage to engulf bacteria cannot efficiently kill them.[50,64] In particular, intracellular killing of *S. aureus* and *E. coli*, which are common causes of bacterial infections in cirrhosis, is impaired in

alcohol-associated cirrhosis.[50] In contrast, another study could not find any differences in neutrophils' capacity for intracellular killing of *S. aureus* in alcohol-associated cirrhosis and PBC despite decreased total bacterial killing, which the authors put down to the decreased percentage of phagocytic neutrophils.[56] In another study, neutrophils from patients with cirrhosis showed impaired killing capacity not only of bacteria but also of fungi, such as *C. albicans*;[49] however, another study found it unchanged.[48]

ROS production

Impaired neutrophil ROS production[51–55,58,59,65–68] and degranulation[50,64] have been described in cirrhosis and might contribute to impaired bacterial killing.[50] The alteration of ROS production in cirrhosis is characterised by an elevated percentage of neutrophils with basal ROS production (so-called "resting burst")[51,55,58,60,62,65] and elevated,[59,65] unchanged[61,62] or reduced[63,67] intracellular basal ROS production (intracellular ROS levels). An increased percentage of neutrophils with basal ROS production has also been reported in ACLF.[53,58,63] Extracellular basal superoxide production is unchanged[50] or elevated in patients with cirrhosis.[48]

An increased percentage of neutrophils from patients with cirrhosis respond to a low physiological stimulus like fMLF,[51,52,55,62] which suggests that these neutrophils have previously been primed by persistent low-grade stimulation with priming agents like lipopolysaccharide (LPS) or tumour necrosis factor-α (TNF-α). However, other studies show it to be unchanged.[60] The intracellular ROS pool of those neutrophils that respond to fMLF stimulation is slightly increased,[65] decreased[67] or unchanged[62] in patients with cirrhosis. Extracellular superoxide release in response to fMLF[66–68] and TNF-α[66] is decreased in patients with cirrhosis. The decrease in superoxide release in response to fMLF is significantly more pronounced in patients with ACLF than in patients with advanced cirrhosis.[18] Notably, priming neutrophils from patients with cirrhosis with TNF-α does not increase their response to fMLF as is usually the case with healthy donor neutrophils.[66]

The number of neutrophils producing ROS in response to a potent stimulus (*E. coli*) is unchanged[51,53,55,60,61,63,65] or decreased[62] in patients with cirrhosis, whereas their intracellular ROS level is increased,[65] decreased[63] or unchanged.[62] In ACLF, ROS production in response to *E. coli* has been shown to be unchanged compared to that in healthy controls.[63] Extracellular superoxide production in response to zymosan (structural component of yeast cell wall) is reduced in patients with cirrhosis.[48,50,66,69] Extracellular hydrogen peroxide levels produced by neutrophils in response to zymosan are not different or higher in patients with cirrhosis compared to healthy controls, which is reflected in the increased hydrogen peroxide/superoxide molar ratio.[50] Interestingly, superoxide and hydrogen peroxide have different effects on cell apoptosis and necrosis: superoxide inhibits apoptosis, whereas hydrogen peroxide promotes cell apoptosis via intracellular acidification and even necrosis when present at very high concentrations.[70,71] Nitric oxide production in response to opsonised zymosan is also increased in neutrophils from patients with cirrhosis.[69]

Changes in ROS production are different in patients with cirrhosis with active infection: basal ROS production and ROS production in response to fMLF have been shown to be unaltered, as have the percentage of neutrophils which produce ROS in response to *E. coli* stimulation; however, the intracellular ROS pool generated in response to *E. coli* is decreased, which might support a concept that cirrhotic neutrophils are exhausted via prior low-grade stimulation and cannot mount an augmented ROS response when they have to fight a real infection.[65]

The aforementioned changes have been shown in studies on cirrhosis of different aetiologies; however, most were performed with neutrophils from patients with alcohol- or HCV-associated cirrhosis. Several studies report no dependence of ROS production on the aetiology of cirrhosis.[53,66] Contrasting findings have been reported on the correlation of changes in ROS production with cirrhosis severity: some studies report no correlation,[51,53] while some studies have found a correlation with disease severity.[65,66]

Degranulation

The intracellular enzyme contents (lysozyme, myeloperoxidase [MPO]) and their release from neutrophil granules upon stimulation with zymosan have been shown to be reduced in neutrophils from patients with cirrhosis; however, the authors of this study claim that the reduced release is not dependent on the reduced enzyme levels inside the granules.[50] A decreased number of neutrophils producing MPO and reduced intracellular MPO levels have been observed in neutrophils isolated from patients with compensated cirrhosis, but not from those with ACLF.[63] Another study reported that intracellular MPO content is not altered in neutrophils from patients with cirrhosis but that its extracellular release in response to fMLF is decreased.[64] MPO activity has been shown to be either decreased[66] or unchanged[64] in neutrophils from patients with cirrhosis. In patients with alcohol-associated cirrhosis, increased mobilisation of MPO to the cell surface of the primary neutrophil granules has been observed.[72]

NET formation

NET formation is a recently discovered mechanism of neutrophil defence.[73] To date, only scarce data are available regarding the role of NET formation in cirrhosis. One research group has shown a significant decrease in NET formation in response to phorbol-12-myristate-13-acetate (PMA) in patients with cirrhosis complicated by spontaneous bacterial peritonitis compared to healthy controls.[74,75] Another group reported later that plasma of patients with decompensated cirrhosis can induce NET formation in neutrophils isolated from healthy controls.[57] This finding is also supported by other studies that reported elevated NET markers, such as H3Cit-DNA[76] and MPO-DNA,[76,77] in the plasma of patients with cirrhosis and ACLF, correlating with cirrhosis severity in these patients.[76] NET formation in response to *E. coli*, fMLF and PMA, as well as spontaneous NET formation, is elevated in patients with compensated cirrhosis and ACLF.[63]

Apoptosis and viability

Current knowledge on neutrophil apoptosis in cirrhosis is not very broad. An increased rate of apoptosis and decreased viability of neutrophils 24 h after isolation from the whole blood of neutropenic patients with viral cirrhosis has been described.[78] Increased apoptosis of neutrophils has also been reported in patients with decompensated cirrhosis.[79]

Summary

Deficiencies in chemotaxis and phagocytosis, as well as decreased neutrophil viability and increased apoptosis, have been reported fairly consistently in patients with cirrhosis across studies, irrespective of differences in cohort selection, or

methods of neutrophil isolation and functional assessment. Although the majority of patients had alcohol-related cirrhosis, some studies show comparable results for other aetiologies of cirrhosis, such as in study cohorts of primarily viral aetiology[31,34,60,63] or patients with PBC.[56] Although most studies have been performed in patients with decompensated cirrhosis, functional impairment has also been shown in patients with compensated cirrhosis.[62,63] The findings regarding chemotaxis and phagocytosis are, however, specific to the stimulus used. For example, chemotaxis towards CXCL8, CXCL1 or casein, and phagocytosis of *C. albicans* are not impaired in cirrhosis, which might indicate that functional defects in cirrhosis are limited to certain pathways. Whether this indicates a true pathophysiological difference making patients with advanced chronic liver diseases more prone to bacterial than to fungal infections or whether this is a methodological bias needs to be further explored. In contrast, the changes in ROS production, degranulation, bacterial killing and NET formation are variable among studies. The most controversial is the nature of alterations in neutrophil ROS production in cirrhosis. Although most studies have reported an elevated number of neutrophils with basal ROS production[51,55,60,65] and ROS production in response to physiological stimuli with low potency like fMLF,[51,52,55] the results on ROS production are highly varied. Most studies have been performed in patients with an alcohol-related aetiology of cirrhosis and decompensated cirrhosis; however, these results are comparable to those obtained for other aetiologies and for compensated cirrhosis.[60,63] Furthermore, some studies report no dependence of ROS production on the aetiology of cirrhosis.[53,66] The variety of methods and experimental conditions used to study ROS production in patients with cirrhosis, often measuring different types of ROS, is the most likely explanation for these differences. The majority of studies measure neutrophil ROS production in cirrhosis with either flow cytometry (*e.g.* based on the conversion of dihydrorhodamine 123 to rhodamine 123, or similar),[58,59,63,65] cytochrome c reduction[18,66–68] or luminol-based[67] assays. Flow cytometry-based assays measure only intracellular ROS production, detecting a mixture of different ROS but with almost no sensitivity for superoxide (*e.g.* in the case of dihydrorhodamine).[80] The cytochrome c reduction assay is able mainly to detect extracellular ROS and, in contrast, detects only superoxide.[81] Luminol-based assays can be used to measure either intracellular or total ROS production (mixture of intracellular and extracellular ROS), depending on the presence or absence of horseradish peroxidase, and to detect the mixture of different ROS.[81,82] Furthermore, the neutrophil preparation technique, whether whole blood or isolated neutrophils are used, and the type of isolation procedure may contribute to the observed differences. When the literature is separated based on methodology used for ROS production assessment, the results become more consistent (Fig. 1). To date, there is no evidence to support the notion that one method is superior in cirrhosis; however, standardisation would be necessary to harmonise the findings and enable translation of neutrophil diagnostics to clinical practice. However, even after standardisation some discrepancies will remain. Despite the methodology used to study neutrophil function, other parameters, such as patient characteristics, cirrhosis aetiology and severity, presence of previous or active bacterial infections, active alcohol drinking, neutrophil isolation technique (given the *ex vivo* fragility of neutrophils), and the combination of all these factors can contribute to the discrepancies in results between studies (Table S1).

Interestingly, recent studies have revealed the importance of neutrophil heterogeneity in different diseases. Neutrophils are no longer perceived as one cell type, but rather as several "types" of cells with particular functions and roles in health and disease. For instance, distant molecular signatures have been described for eight neutrophil subpopulations[83] and different functional profiles have been described for five neutrophil subgroups.[84] The role of neutrophil heterogeneity in cirrhosis and its interplay with neutrophil dysfunction are yet to be determined.

Taken together, circulating neutrophils in cirrhosis are altered numerically but, more importantly, exhibit multiple functional deficiencies, starting from the inability to migrate to the infection site and subsequent inability to internalise and kill pathogens. The loss of neutrophil antimicrobial function, particularly changes in ROS production and phagocytosis in cirrhosis, contributes to the development of bacterial infections, organ failure and mortality.[34,51,53]

This underscores the importance of identifying the mechanisms underlying neutrophil dysfunction in cirrhosis, which will allow for the development of targeted therapies for the prevention and treatment of bacterial infections in cirrhosis.

Causes of neutrophil dysfunction in cirrhosis

First, the course of cirrhosis *per se* contributes to altered neutrophil functionality. Portal hypertension is associated with upregulation of the neutrophil chemoattractant CXCL1 in primary liver sinusoidal endothelial cells from mice and with NET formation.[85] Alterations in Toll-like receptor (TLR)4 expression and signalling not only contribute to inflammation and endothelial dysfunction but also neutrophil dysfunction in patients with cirrhosis.[86] Thrombocytopenia is also commonly described in cirrhosis and its crosstalk with neutrophil function has been described, *e.g.* platelet transfusions in patients with thrombocytopenia result in further increases in CD11b expression on neutrophils.[87] Despite this, one of the important questions is whether the neutrophil dysfunction is primarily a cellular problem or if it is caused by extracellular factors. There are multiple studies that attempt to support one or other of these concepts.

Proposed intrinsic defects

Some studies propose that cellular defects of neutrophils are responsible for their dysfunction in cirrhosis. In one study, sera from patients with decreased neutrophil locomotion does not cause a similar defect in chemotaxis in healthy control neutrophils.[30] However, in the same study, sera from patients with cirrhosis exhibited reduced chemoattractant activity for healthy donor neutrophils compared to healthy donor serum, which could indicate that changes in serum content also potentially contribute to the decreased chemotaxis of patients' neutrophils.[30] Reduced intracellular glutathione levels – a detoxifier of hydrogen peroxide – are reported in neutrophils from patients with cirrhosis, which could explain the prevalence of hydrogen peroxide over superoxide. Lower levels of glutathione have been associated with higher levels of hydrogen peroxide production and lower hydrogen peroxide/superoxide ratio, degranulation and intracellular killing of *E. coli*. In this study, cirrhotic serum does not cause any changes in phagocytic function or intracellular bacterial killing of healthy donor neutrophils, suggesting underlying cellular defects, including intracellular glutathione deficiency.[50] Dysfunctional neutrophils from patients with

cirrhosis have decreased phospholipase C (which is involved in superoxide production) activity; however, the reason for this is unknown.[66] Furthermore, changes in neutrophil glycolytic metabolism and other transcriptional profile alterations, including induction of granule genes and downregulation of cell migration and cell cycle genes have been reported in patients with decompensated cirrhosis with ACLF,[18] and are likely to be associated with the impaired anti-pathogenic function of neutrophils.[18,63,88]

Proposed serum defects

The majority of studies investigating neutrophil dysfunction have proposed that changes in serum content in patients with cirrhosis initiate the development of neutrophil dysfunction (Fig. 2, Table 1). Almost all of the functional deficiencies outlined have been shown to be transferrable with patient sera. Neutrophils from patients with cirrhosis have a defect in migration towards zymosan only in the presence of autologous plasma and not healthy control plasma, and these defects vary between the aetiologies of cirrhosis (defects are present in alcohol-associated and cryptogenic cirrhosis, but not PBC).[33] Healthy donor neutrophils have been shown to have decreased capacity to kill *C. albicans* following incubation with cirrhotic serum.[49] Decreased phagocytic capacity for *E. coli,* but unchanged (though a tendency toward an increase) basal ROS production (percentage of neutrophils), has been shown in healthy donor neutrophils after incubation with cirrhotic plasma, dependent on cirrhosis severity but independent of cirrhosis aetiology.[61] In another study, incubation with cirrhotic plasma promoted an increase in the number of healthy donor neutrophils with basal ROS production and a decrease in phagocytosis. Interestingly, neutrophil dysfunction in cirrhosis seems to be reversible with a restoration of function observed following incubation with healthy donor plasma.[51] Neutrophil phagocytosis and intracellular killing of *S. aureus* are not affected in cirrhotic neutrophils

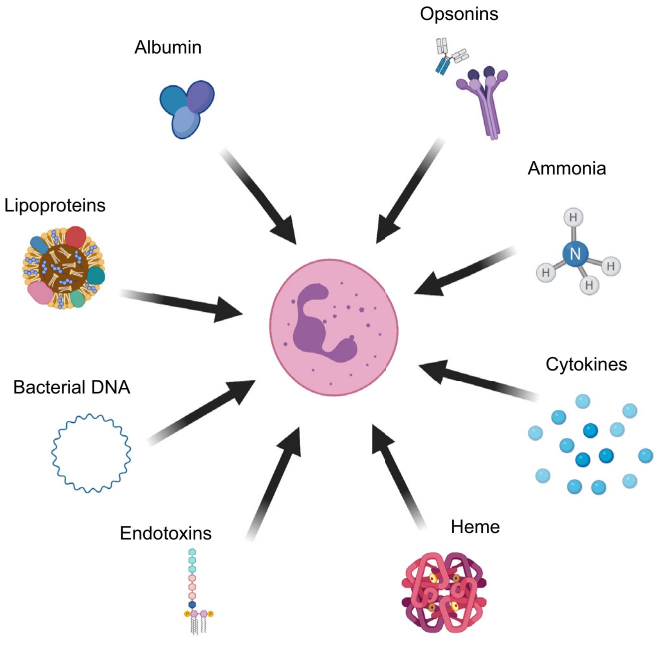

Fig. 2. Overview on proposed serum factors that might directly or indirectly affect neutrophil function in cirrhosis. Created with Biorender.com.

incubated with AB serum despite being dysfunctional in the presence of autologous serum.[56] Patients' plasma also influences healthy donor neutrophil degranulation, decreasing MPO release in response to fMLF.[64] Our research group recently showed that serum components more than 30 kD in size are responsible for the changes in neutrophil phagocytic function.[54]

Increased gut permeability in cirrhosis results in various bacteria and bacteria-derived molecules, like endotoxins, getting from the gut lumen to the systemic circulation.[89] These substances in the serum of patients with cirrhosis are thought to have an influence on neutrophil function via persistent low-grade stimulation.

Endotoxins

Endotoxins cause an increase in the percentage of neutrophils with basal ROS production in *in vitro* experiments with healthy donor neutrophils and a decrease in phagocytic capacity in experiments with cirrhotic neutrophils. Plasma endotoxin removal strategies prevent the deleterious effects of patients' plasma on healthy donor neutrophils.[51] However, despite *in vitro* effects of LPS on neutrophil function, the levels of bacterial endotoxin in the plasma of patients with cirrhosis do not correlate with the defects in phagocytic function[55,61] and basal ROS production.[61] Endotoxin receptors TLR2 and TLR4 are upregulated in healthy donor neutrophils after incubation with cirrhotic plasma[61,90] and in neutrophils from patients with cirrhosis.[52] TLR2 is upregulated only after incubation with plasma from patients with alcohol-associated cirrhosis, while TLR4 is upregulated in neutrophils treated with plasma from patients with both alcohol-associated and viral cirrhosis.[61] Inhibition of TLR2 and TLR4 decreases the number of neutrophils with basal ROS production caused by incubation with cirrhotic serum, but further impairs phagocytic capacity.[90] LPS-binding protein (LBP) levels are elevated in patients with cirrhosis compared to healthy controls, being associated with lower intracellular basal ROS production[65] and with the development of severe bacterial infections.[91] LBP enhances the effects of LPS on immune cells.[92]

Bacterial DNA

Bacterial DNA itself may also be responsible for changes in neutrophil function in cirrhosis. Bacterial DNA is present in the serum of some patients without active infection,[93–95] but is not associated with the Child-Pugh score nor clinical characteristics of these patients.[93] The presence of bacterial DNA in the serum of patients with cirrhosis is associated with higher levels of cytokines such as TNF-α, IFN-γ, IL-12 and nitric oxide.[95] Interestingly, higher cytokine levels in the sera of patients with bacterial DNA are independent of their LPS or LBP serum levels.[96] In one study, the bacterial DNA sensing receptor in neutrophils, TLR9, has been shown to be more highly expressed in neutrophils from patients with cirrhosis than healthy controls.[52] However, in other studies, TLR9 has not been found to be upregulated in patients with cirrhosis[61,90] and circulating bacterial DNA measured in the plasma of patients with cirrhosis could neither be associated with the changes in neutrophil phagocytic and basal ROS production, nor with patient mortality.[61]

Albumin

Albumin is synthesised in the liver and, therefore, its concentration is reduced in cirrhosis.[97] Furthermore, the structure of albumin is also altered in cirrhosis, mainly via oxidation, plasma levels of oxidized albumin increase further in ACLF.[98,99] Albumin

Table 1. Overview of serum factors thought to contribute to neutrophil dysfunction in cirrhosis.

Serum factors	Evidence for the role in neutrophil dysfunction development	Evidence against the role in neutrophil dysfunction development
Endotoxins	• Increased basal ROS production[51]<br>• Decreased phagocytosis[51]<br>• Beneficial effects of endotoxin removal from plasma[51]	• No correlation with defect in ROS production and phagocytosis[55,61]
Bacterial DNA	• Correlation with higher levels of TNF-α, IFN-γ, IL-12 and nitric oxide[95]	• No association with severity and clinical parameters[93]<br>• No association with ROS production and phagocytic function[61]
Albumin	• Decreased albumin binding capacity is associated with cirrhosis severity[100] and mortality[101]<br>• Decreased basal ROS production and improved phagocytosis *in vitro*[90]<br>• Decreased neutrophil superoxide production[105]	• No association with *C. albicans* killing capacity[49]<br>• No association with phagocytosis and intracellular killing capacity[50]
Ammonia	• Impaired phagocytosis of *E. coli,* increased basal ROS production[107]<br>• Correlation with decreased phagocytic activity[53]	
Lipoproteins	• Correlation with disease severity, increased levels of TNF-α, IL-8, IL-6[108]<br>• Higher levels of IgG autoantibodies against oxidised low-density lipoproteins correlated with higher integrated intracellular ROS production level in response to *E. coli*[65]	
Cytokines	• Correlation with increased numbers of neutrophils with basal ROS production and ROS producing neutrophils in response to *E. coli.*[53]<br>• Inhibited neutrophil phagocytosis and bactericidal activity[112]<br>• Improved neutrophil chemotaxis towards IL-8[42,116]<br>• Calprotectin is predictive for survival and infections[120,121]<br>• Accelerate neutrophil clearance during inflammation and impair CXCL1 production[113]<br>• Increase neutrophil regress from the bone marrow and their recruitment to the tissues[114]<br>• Trigger degranulation and NETs formation[115,118]<br>• Delays neutrophil apoptosis in presence of PBMCs[117]	• Calprotectin levels similar between cirrhotic patients and healthy volunteers[120]<br>• IL-6 does not influence neutrophil apoptosis, priming or adhesion molecule expression[116]
Iron metabolism parameters	• Delayed apoptosis of neutrophils[125]<br>• Activated neutrophil chemotaxis, ROS production and IL-8 expression[128]<br>• Impaired phagocytosis and migration of neutrophils[127,129,130]<br>• Reduced ROS production[131,134]<br>• Correlation with intracellular ROS production in response to fMLF, opsonized zymosan, PMA.[133]<br>• Decreased NET formation.[134]	• No effect on neutrophil ROS production, degranulation of azurophilic granules, phagocytosis and bacterial killing[134]
Serum opsonins	• Association with chemotactic inhibitory activity[35]<br>• Decreased opsonisation of *E. coli*[137]	• No association with neutrophil chemotaxis, phagocytosis or intracellular killing capacity defects[30,50]<br>• No correlation with the level of serum chemotactic inhibitory activity[32]<br>• No correlation with neutrophil locomotion defect[33]

CXCL1, CXC motif ligand 1; fMLF, N-Formyl-met-leu-phe; IFN, interferon; IL-, interleukin-; NET, neutrophil extracellular trap; PBMCs, peripheral blood mononuclear cells; PMA, phorbol-12-myristate-13-acetate; ROS, reactive oxygen species; TNF-α, tumour necrosis factor-α.

binding capacity is decreased in patients with decompensated cirrhosis, which negatively correlates with the MELD (model for end-stage liver disease) score[100] and increased mortality.[101] Interestingly, neutrophil to albumin ratio is linked to mortality in patients with decompensated cirrhosis.[102,103] Given the ability of albumin to bind bacterial products, ROS, and nitric oxide,[104] the changes in its abundance and structure might contribute to neutrophil dysfunction. The addition of albumin to incubation media has been shown to decrease the number of neutrophils with high basal ROS production and reverse phagocytic capacity defects caused by plasma from patients with cirrhosis.[90] Carbamylated albumin dose-dependently inhibits superoxide production by neutrophils activated by type I collagen.[105] However, one study shows that patients with cirrhosis with dysfunctional neutrophil phagocytosis and intracellular killing capacity have comparable serum albumin levels to patients with cirrhosis without neutrophil functional defects, indicating that it is not the quantity but the functionality of albumin that is important in

cirrhosis-associated neutrophil dysfunction.[50] Furthermore, the *C. albicans* killing capacity of cirrhotic neutrophils does not correlate with serum albumin levels.[49]

Ammonia

Ammonia levels in the serum of patients with cirrhosis predict organ failure and mortality.[106] Neutrophils from rats subjected to ammonia supplementation and healthy donor neutrophils incubated with ammonia exhibit impaired phagocytosis of *E. coli* and an increased number of neutrophils with basal ROS production, due to the ability of ammonia to cause cell swelling.[107] Decreased neutrophil phagocytic activity correlates with increased plasma ammonia levels in patients with cirrhosis.[53]

Lipoproteins

Patients with cirrhosis have lower levels of high-density lipoprotein (HDL) cholesterol and apolipoprotein A1, which further decrease upon decompensation, correlating with increased levels of TNF-α, IL-8, IL-6 and severe bacterial infection, and predicting patient mortality.[108] The important function of HDL, similar to that of albumin, is to neutralize LPS;[109] therefore, its low abundance in cirrhosis could be a reason for higher LPS serum concentrations and low-grade inflammation. Furthermore, HDL composition and function is altered in cirrhosis.[110] Higher levels of IgG autoantibodies against oxidised low-density lipoproteins are correlated with higher integrated intracellular ROS production in response to *E. coli* in neutrophils from patients with cirrhosis.[65]

Cytokines

Elevated pro- and anti-inflammatory cytokines, such as TNF-α, IL-6, IL-1β, IL8 and IL10, have been reported in many but not all studies.[49,52,53,55,61] Increasing numbers of neutrophils with basal ROS production correlate with increased TNF-α, IL-6, IL-8 and IL-10, whereas increasing numbers of neutrophils producing ROS in response to *E. coli* correlate with increased IL-1β, IL-8, IL-1 and IL-17 levels.[53] Interestingly, patients with ACLF have even higher levels of TNF-α, IL-6, IL-8, IL-1β, IL-12, IL-1RA, IL-10, granulocyte colony stimulating factor (G-CSF) and granulocyte macrophage colony stimulating factor (GM-CSF) in blood compared to patients with decompensated cirrhosis.[19,44,99] TNF-α is a known priming agent.[111] IL-10 inhibits neutrophil phagocytosis and bactericidal activity.[112] IL-6 has been shown, on the one hand, to accelerate neutrophil clearance during inflammation and to impair CXCL1 production (which is a chemoattractant for neutrophils) via the IL-6/gp130/STAT3 pathway;[113] on the other hand, IL-6 has been shown to increase neutrophil regress from the bone marrow and recruitment to target tissues.[114] IL-8 is a known neutrophil chemoattractant and has been shown to trigger degranulation and NET formation.[115] IL-33 treatment of neutrophils from patients with cirrhosis improves their chemotaxis towards IL-8[42] and IL-6 has been reported to increase neutrophil migration towards IL-8; however, it does not influence neutrophil apoptosis, priming or adhesion molecule expression.[116] IL-1β delays neutrophil apoptosis in the presence of peripheral blood mononuclear cells[117] and induces NET formation, which can be abrogated by IL-1RA.[118] IL-17-activated pericytes produce chemokines, which stimulate neutrophil production of pro-inflammatory molecules, prolong neutrophil survival and increase neutrophil phagocytic capacity.[119] Serum calprotectin is an important biomarker of neutrophil activation. Serum levels of calprotectin in patients with compensated and decompensated alcohol-induced cirrhosis are similar to those from healthy volunteers and are predictive of survival and recurrent infections, independent of the severity of cirrhosis.[120] Another study has shown increased calprotectin levels in patients with stable cirrhosis and acute decompensation of cirrhosis of different aetiologies, which correlates with the severity of the disease, ACLF and infection, and is associated with poor survival in acute decompensation but not in ACLF.[121]

Iron metabolism parameters

Disturbances in iron metabolism are known in patients with cirrhosis.[122] Iron metabolism parameters influence neutrophil function. Heme is a part of haemoglobin and excessive free heme is released to the circulation in the case of haemolysis,[123] which is common for patients with cirrhosis.[60] Hemin, which is different from heme as it contains ferric and not ferrous ion, is also increased in haemolysis.[124] Heme delays apoptosis of neutrophils[125] and induces migration of and ROS production by neutrophils.[126] In contrast, another study shows impaired phagocytosis and migration of neutrophils in response to heme, which can be explained by completely different experimental design of chemotaxis experiments, with heme used not as a chemoattractant for human neutrophils *in vitro*, but as a treatment for mice in *in vivo* experiments.[127] Hemin activates neutrophil chemotaxis, ROS production and IL-8 expression.[128] The ferritin-containing fraction of serum significantly decreases neutrophil phagocytosis.[129] High serum ferritin levels have been linked to impaired neutrophil phagocytosis and chemotaxis.[130] Some authors have linked ferritin to reduced ROS formation.[131] Higher ferritin levels in serum are associated with higher risk of bacterial infections and lower ferritin levels are associated with disease progression in patients with cirrhosis.[132] Intracellular ROS production in response to fMLF, opsonised zymosan, or PMA is increased and correlates positively with plasma transferrin saturation but not with ferritin level in patients with hereditary hemochromatosis (liver disease severity is unclear from the paper).[133] Furthermore, this patient cohort also shows increased neutrophil phagocytic capacity and decreased L-selectin/CD62L surface expression compared to healthy controls.[133] Mouse models of hereditary haemochromatosis show decreased NET formation and ROS production in response to PMA, but no defect in *E. coli* phagocytosis or mobilisation of azurophilic granules.[134] Healthy donor neutrophils pre-treated with ferrous ions or holo-transferrin decrease NET formation, but holo-transferrin does not affect neutrophil ROS production, degranulation of azurophilic granules, phagocytosis nor bacterial killing.[134] A high-iron diet in mice results in decreased NET formation and ROS production by neutrophils in response to PMA.[134]

Serum opsonins

Opsonisation of bacteria helps neutrophils recognise pathogens and promotes phagocytosis and killing. The main opsonins are immunoglobulins and components of the complement system.[135] Defects in serum opsonisation have been described in patients with cirrhosis due to deficiency of opsonisation factors.[136] IgA, IgG and IgM have been shown to be increased in sera from patients with cirrhosis. The opsonic effects of patients' sera on *E. coli* were found to be decreased compared to controls in this study.[137] However, IgG, IgM and IgA levels in serum from

patients with and without defects in neutrophil chemotaxis, phagocytosis or intracellular killing capacity, have not been reported to differ in some studies.[30,50] Some studies have reported no correlation of higher IgA with the level of serum chemotactic inhibitory activity[32] or defects in neutrophil locomotion,[33] while another study found a correlation of increased levels of IgA and IgG with chemotactic inhibitory activity in patients with cirrhosis, and demonstrated that IgA removal restores normal chemotactic activity.[35] Patients with cirrhosis were shown to have normal levels of C3 and C4 in one study,[56] but decreased levels in another study.[137] No correlation of defects in neutrophil locomotion with serum levels of C3 and C5 has been shown.[33]

Antibiotics

A large number of patients with cirrhosis are prescribed antibiotics; therefore, serum concentrations of antibiotics could also affect neutrophil function in these patients. Apart from their antibacterial effects, some antibiotics are also known to exhibit immunomodulatory properties. For example, ceftaroline induces CD11b and decreases CD62L expression, which tends to increase neutrophil survival in response to *S. aureus*-derived lipoteichoic acid. Vancomycin, as well as dalbavancin, teicoplanin, sulfametrole/trimethoprim and ceftazidime/avibactam have been shown to decrease CXCL8 release in neutrophils.[138,139] Dalbavancin and teicoplanin inhibit neutrophil ROS production. Dalbavancin also inhibits neutrophil bactericidal activity. Ceftazidime/avibactam inhibit neutrophil burst in response to fMLF/cytochalasin B.[139] Azithromycin and chloramphenicol decrease NET formation *in vitro*.[140] Further effects of different antibiotic types on neutrophil functions are reviewed in 141–143.

Other factors

EMR2 (EGF-like molecule containing mucin-like hormone receptor 2) expression is increased in patients with cirrhosis, dependent on severity and the presence of bacterial infections, and is a predictor of mortality. However, ligation of EMR2 has failed to improve the phagocytic capacity of cirrhotic neutrophils despite increasing intracellular ROS production in response to *E. coli*.[59]

Potential players in neutrophil function regulation in cirrhosis

The contribution of serum factors discussed above to cirrhosis-associated neutrophil dysfunction is rather controversial given the contrasting findings regarding their effects on neutrophils as well as their association with bacterial infections and mortality in cirrhosis. Further investigations of causes for neutrophil deficiency in cirrhosis are necessary. Particular attention should be given to serum components larger than 30 kDa.[54]

Bile acids are among these components. In systemic circulation, they are bound mainly to albumin and lipoproteins, and are therefore found in the serum fraction larger than 30 kD.[144–146] Furthermore, serum bile acids are highly elevated in liver diseases, including cirrhosis,[147–153] which makes them a potential player in the regulation of neutrophil function in cirrhosis.

Bile acids are broadly known for their functions in the gastrointestinal tract, *e.g.* cholesterol elimination and lipid emulsification. However, the bile acid receptors farnesoid X receptor[154–156] and Takeda G protein-coupled receptor 5[157,158] have been shown to play a role in many metabolic processes and the immune response.[159,160] Approximately 95% of bile acids are reabsorbed in the intestine by the apical sodium-dependent bile acid transporter or through passive diffusion, and around 0.5 mg of bile acids enter the systemic circulation each day.[161]

Several studies describe neutrophil function in rat or mouse cholestatic models, associating cholestasis with either increased[162] or decreased[163] ROS production, decreased bacterial killing,[163] neutrophil adhesion[164] and increased migration,[162] unchanged[163] or increased[162] phagocytosis, unchanged degranulation,[163] and increased Mac-1 expression and L-selectin shedding.[165] Bile, unconjugated lithocholic acid, CDCA, deoxycholic acid and cholic acid (at very high concentrations) potentiate ROS release in primed rat neutrophils. Only unconjugated lithocholic acid also caused superoxide production in rat neutrophils that had not been pre-activated.[166] The priming effect of lithocholic acid on rat neutrophils activated with PMA, fMLF or calcium ionophore has been shown, as has its inhibitory activity against beta-glucuronidase release from fMLF-activated neutrophils.[167] However, the composition and functions of bile acids in rats are significantly different to those in humans; therefore, it is difficult to draw conclusions about bile acids' effects on human neutrophils based on the results from rat and mouse studies.[168–170]

There are only a few studies describing the effects of bile acids on human neutrophils. CDCA and ursodeoxycholic acid (UDCA) serum levels have been associated with neutrophil phagocytosis and ROS production in patients with cirrhosis.[62] Sera from patients with obstructive jaundice have been shown to induce ROS production in healthy donor neutrophils.[171] Individual bile acids have been studied only in regard to chemotaxis, intracellular calcium mobilisation, phagocytosis and ROS production in human neutrophils. Unconjugated CDCA and UDCA have been shown to reversibly inhibit chemotaxis of human neutrophils in response to fMLF, but these effects have not been shown for lithocholic acid and cholic acid.[172] In other studies, unconjugated CDCA, UDCA[47] and deoxycholic acid (DCA)[173] inhibit chemotaxis towards fMLF in human neutrophils, but DCA does not inhibit chemotaxis towards C5a or IL-8.[173] Unconjugated and conjugated forms of CDCA, UDCA (at high concentrations)[47] and unconjugated DCA (reversibly)[173] have been shown to inhibit calcium flux in healthy donor neutrophils in response to fMLF, but not in response to C5a or IL-8. Bile acids differentially affect ROS production with total lithocholic acid triggering neutrophil ROS production in the absence of other stimuli. Total CDCA and lithocholic acid inhibit ROS production in response to fMLF while total CDCA and DCA inhibit ROS production and phagocytosis in response to *E. coli*[62] (Fig. 3). Hence, the effects of bile acids on human neutrophils are not yet well described. This underscores the necessity to further investigate the effects of bile acids on neutrophils as potential contributors to cirrhosis-associated immune dysfunction.

As mentioned, bile acids are transported in the systemic circulation with the help of proteins and lipoproteins, mainly albumin.[144,145] Albumin plays a role in neutrophil dysfunction, including defects in phagocytosis and basal ROS production,[90] and is associated with bacterial infections in cirrhosis.[174] Albumin has several identified bile acid-specific binding sites.[175,176] The previously described albumin dysfunction in cirrhosis[177] might change its affinity for bile acids. Furthermore, in conditions of albumin deficiency, bile acids change their transporter preferences to lipoproteins, which in turn might facilitate their interaction with cells and tissues, including neutrophils,[146] and

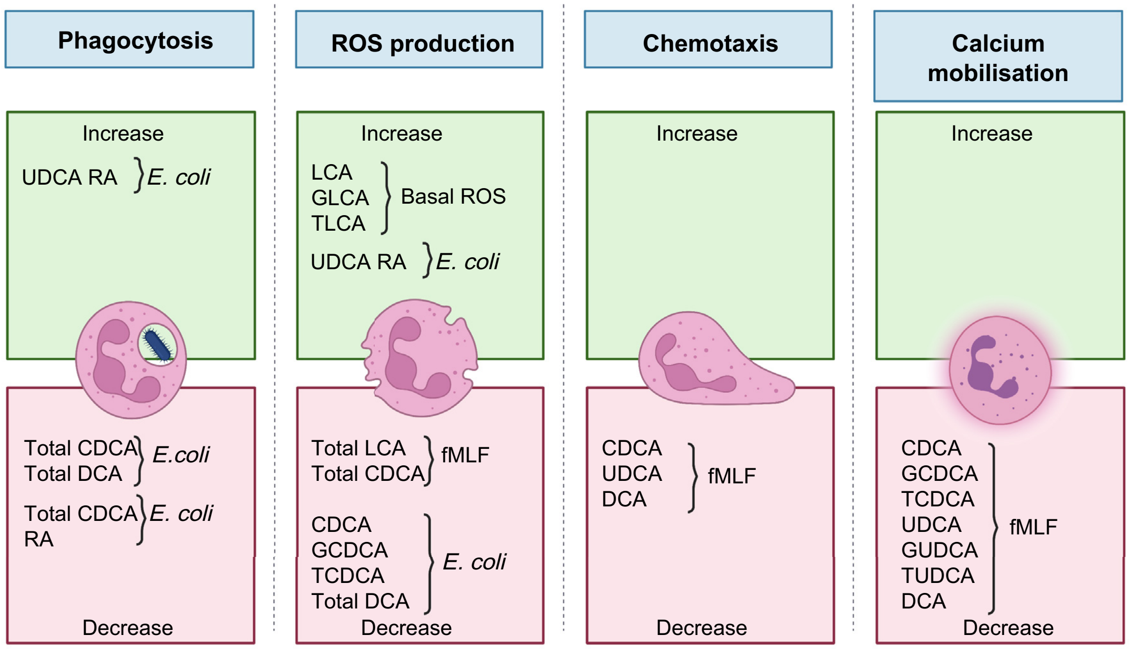

Fig. 3. Current knowledge on how bile acids affect human neutrophil function. CDCA, chenodeoxycholic acid; DCA, deoxycholic acid; fMLF, N-Formyl-met-leu-phe; GCDCA, glycochenodeoxycholic acid; GLCA, glycolithocholic acid; GUDCA, glycoursodeoxycholic acid; LCA, lithocholica acid; RA, relative abundance in serum; ROS, reactive oxygen species; TCDCA, taurochenodeoxycholic acid; TLCA, taurolithocholica acid; total DCA, sum of deoxycholic, taurodeoxycholic and glycodeoxycholic acids; total LCA, sum of LCA, TLCA and GLCA; total CDCA, sum of CDCA, TCDCA and GCDCA; TUDCA, tauroursodeoxycholic acid; UDCA, ursodeoxycholic acid. Created with Biorender.com.

potentially cause their higher intracellular accumulation. Therefore, serum concentrations of bile acids, which are measured in cirrhosis, might underestimate the concentrations to which neutrophils are actually exposed, making bile acids that are present at relatively low serum concentrations, such as lithocholic acid and UDCA, more pathophysiologically relevant. As some lipoproteins are deficient in sera of patients with cirrhosis,[110] the pathophysiological importance of other potential bile acid protein transporters should be considered. Therefore, serum factors responsible for cirrhosis-associated immune dysfunction should be further studied together rather than separately, as they might be interdependent.

Apart from albumin, other serum proteins could contribute directly or indirectly to neutrophil dysfunction, *e.g.* via transporting bile acids. For instance, haptoglobin is one of the most downregulated proteins in sera of patients with cirrhosis compared to healthy controls and is associated with neutrophil function.[54] Another study shows the whole range of serum proteins which are significantly associated with neutrophil function changes in patients with cirrhosis.[57]

Furthermore, increased ANCA (anti-neutrophil cytoplasmic antibodies) levels have been observed in cirrhosis and were associated with disease severity and risk of infections.[178] Moreover, ANCA induce NET release,[179,180] suggesting their possible contribution to the development of defects in neutrophil function in cirrhosis.

Proposed and potential therapeutic strategies

The search for ways to prevent and treat the development of cirrhosis-associated immune dysfunction, including neutrophil dysfunction, is ongoing. The reversibility of neutrophil dysfunction reported in several studies[49,51] suggests that it could be possible to develop therapeutic strategies aimed at restoration of neutrophil function.

Therapeutic strategies targeting neutrophil dysfunction in cirrhosis supported by clinical studies

Previous studies have shown the beneficial effects of albumin administration on overall survival and bacterial infection rates in patients with cirrhosis;[181,174] however, its effects on neutrophil function have not been investigated within clinical studies. *In vitro* experiments have hinted at the possibility of restoring neutrophil function with albumin treatment.[90] There is also indirect clinical evidence of possible albumin-related effects on neutrophil functionality, as some studies reported decreased plasma cytokine levels,[174,182,183] which have been shown to correlate with changes in neutrophil ROS production,[53] following albumin treatment. Albumin appears to be a promising immunomodulating strategy in cirrhosis, though more clinical evidence is required.[184]

Ex vivo studies assessing the removal of endotoxin from the sera of patients with cirrhosis support the hypothesis that this can be a potential therapeutic approach to combat neutrophil dysfunction in cirrhosis.[51] We have identified only one study to date that explored the effects of endotoxin removal from the plasma of patients with cirrhosis; however, they have not yet reported whether they observed any effects on neutrophil function.[185]

Further strategies suggested in cirrhosis include treatment with G-CSF or GM-CSF. It has been shown in *ex vivo* studies, for instance, that treatment with either G-CSF or GM-CSF can restore neutrophil's ability to inhibit *C. albicans* growth[49] and that G-CSF

Table 2. Clinical studies that evaluated different therapeutic strategies in cirrhosis with potential effects on neutrophil function.

Study	Intervention	Participants	Primary endpoint	Neutrophil-related effects	Overall effects on disease course	Adverse events
**Direct modulation**						
Caraceni P *et al.*, 2018[181] NCT01288794	Human albumin (intravenously 40 g twice weekly for 2 weeks, and then 40 g weekly) + standard medical treatment for up to 18 months *vs.* standard medical treatment	431 patients with cirrhosis and uncomplicated ascites	18-month mortality	Not tested	18-month survival is significantly higher in patients with albumin supplementation	No adverse events attributed to albumin treatment
Fernández J *et al.*, 2020[174] NCT02034279	Antibiotics plus albumin or antibiotics alone; 1.5 g/kg body weight at Day 1 and 1 g/kg body weight at Day 3 up to a maximum of 150 g and 100 g, respectively, in patients with body weight >100 kg, and a minimum of 90 g and 60 g, respectively, in patients with body weight <60 kg. Then days 3 and 7, and weekly until infection resolution	118 patients with advanced cirrhosis and non-SBP infections	In-hospital mortality	Decreased plasma IL-6 in patients with albumin supplementation	Less nosocomial in fections in patients with albumin supplementation	No adverse events attributed to albumin treatment
Fernández J *et al.*, 2019[183] NCT00968695 NCT03451292	Long-term: 12 weeks treatment with low doses (1 g/kg body weight every 2 weeks) and high doses (1.5 g/kg every week) of albumin; Short-term: 1-week treatment with antibiotics alone or the combination of albumin and antibiotics (1.5 g/kg on day 1 and 1 g/kg on day 3)	18 patients without bacterial infections (the Pilot-PRECIOSA study) with long-term albumin treatment; 78 patients with bacterial infections (INFECIR-2 study) with short-term albumin treatment		Reduced IL-6, G-CSF, IL-1 RA, vascular endothelial growth factor upon high-dose albumin treatment	Increased serum albumin level, improved circulation stability and left ventricular function	
Guevara M *et al.*, 2012[212] NCT 00124228	Antibiotics and albumin (1.5 g/kg at diagnosis and 1 g/kg at day 3) or antibiotics alone	110 patients with cirrhosis and infections other than SBP	Survival at 3 months	Not tested	Improved renal and circulatory function, survival at 3 months did not change, but albumin is predictive for survival	
Sola E *et al.*, 2018[213] NCT00839358	One-year treatment with midodrine and albumin (40 g/15 days) or placebos	196 patients with cirrhosis and ascites awaiting liver transplantation	Incidence of any complication (renal failure, hyponatremia, infections, hepatic encephalopathy or gastrointestinal bleeding)	No significant changes in plasma levels of IL-1β, IL-6 and TNF-α	No difference in the probability of devel oping complications of cirrhosis or one-year mortality	No adverse events attributed to albumin treatment
China L *et al.*, 2021[214]	Albumin treatment for 2 weeks or until discharge. Median total infusion of albumin of 200 g	777 patients with cirrhosis	New infection, kidney dysfunction, or death between days 3 and 15 after the initiation of treatment	Not tested	No significant difference in primary endpoint	More severe adverse events in albumin treated group
Chen *et al.*, 2009[182]	Antibiotics and albumin infusion (20% 50 cc every day for 3 days) or antibiotics alone	30 patients with cirrhosis with SBP, 24 patients with cirrhosis with sterile ascites		Reduced plasma levels of IL-6 and TNF-α, ascitic fluid levels of IL-6, TNF-α, endotoxin, unchanged levels of nitric oxide products		No adverse events attributed to albumin treatment
Agarwal *et al.*, 2021[185] NCT03065699	DIALIVE (removes endotoxin from plasma) is administered for a median of 3 sessions (range 1–5) in first 3-days (range 1–6) for a median of 8 h (7–12) each day or standard of care	32 patients with ACLF and alcohol-associated cirrhosis	Safety	Not reported	Increases proportion of patients resolving ACLF and reduces time to resolution, significantly lower MELD score	

(*continued on next page*)

Table 2 (*continued*)

Study	Intervention	Participants	Primary endpoint	Neutrophil-related effects	Overall effects on disease course	Adverse events
Rolando *et al.*, 2000[186]	G-CSF to four groups (each n = 6) of ALF patients; a daily infusion at 25, 50, 100 or 150 μg/m^2 for 2 weeks	24 patients with ALF receiving G-CSF and 8 ALF patients which did not receive G-CSF	Neutrophil phagocytosis and killing of *Staphylococcus aureus* and super oxide production at 24 and 96 h after G-CSF administration	50, 100 or 150 μg/m^2 G-CSF treatment resulted in significantly increased phagocytosis and killing at 96 h. 50 and 150 μg/m^2 of G-CSF resulted in increased superoxide production at 96 h	Not reported.	Two patients receiving 150 μg/m^2 G-CSF treatment had adverse effects possibly related to G-CSF: leucocytosis and increase in gamma glutamyl transpeptidase, aspartate aminotransferase, alkaline phosphatase on last day of G-CSF administration
Sehgal *et al.*, 2022[215]	250 μg of GM-CSF intravenously for about 6 h daily for 5 days	164 decompensated patients with cirrhosis with or without sepsis and 15 healthy controls		Neutrophil count is decreased after 1 day of GM-CSF therapy	Improved survival	
Venkitaraman *et al.*, 2022[187] NCT03911037	G-CSF 5 μg/kg subcutaneously, 12 hourly for 5 consecutive days, a total of 4 cycles, once every 3 months	70 patients with decom pensated cirrhosis	12-month overall survival	Not studied, but decreased rate of infections observed upon treatment	Survival not improved	Treatment-related adverse events in 23 patients, mostly backache
Kedarisetty *et al.*, 2015[216] NCT01384565	Subcutaneous G-CSF (5 μg/kg/d) for 5 days and then every third day (12 total doses) and subcutaneous darbopoietin α (40 mcg/wk) for 4 weeks	55 patients with decom pensated cirrhosis	Survival at 12 months	Not studied	Higher survival in patients upon treatment	No adverse events attributed to G-CSF treatment
Prajapati *et al.*, 2017[217] NCT02642003	GCSF 300 μg twice daily for 5 days	126 patients with decom pensated cirrhosis with GCSF plus standard medical therapy and 127 patients with decompensated cirrhosis and only standard medical therapy	Number of participants alive at 6 months	Not studied	Higher cumulative survival in patients with GCSF treatment	No adverse events attributed to G-CSF treatment
Newsome *et al.*, 2018[218] 2009-010335-41	Subcutaneous injections of G-CSF (lenograstim; Chugai Pharmaceuticals, London, UK) at 15 μg/kg bodyweight daily for 5 consecutive days	26 cirrhotic patients with G-CSF, 28 cirrhotic patients with G-CSF plus stem-cell infusion, 27 cirrhotic patients with standard care	Change in MELD score at 90 days from baseline and the trend of treatment activity established by incorporating MELD score measured at baseline and days 30, 60 and 90	Not studied	No improvement in MELD score	No adverse events attributed to G-CSF treatment
De *et al.*, 2021[188] NCT03415698	5 days of G-CSF (5 μg/kg subcutaneously every 12 h) every 3 months, with standard medical therapy, in 4 cycles or standard medical therapy alone	100 patients with decompensated cirrhosis (50 patients on G-CSF plus standard medical therapy, 50 patients on standard medical therapy)	Survival at 12 months	Not studied, but recorded fewer infections in the group treated with G-CSF	Higher survival at 12 months	Adverse effects of G-CSF in 37 patients, with the most common being fatigue (46%) and back pain (46%). Patients treated with G-CSF had leucocytosis on day 6 of each treatment cycle

(*continued on next page*)

Table 2 (*continued*)

Study	Intervention	Participants	Primary endpoint	Neutrophil-related effects	Overall effects on disease course	Adverse events
Verma *et al.*, 2018[189] NCT02451033	G-CSF (5 µg/kg subcutaneously every 12 h for 5 days, then every 3 months for 3 days until 12 months; four cycles)	65 patients with decompensated cirrhosis. 23 patients on SMT plus G-CSF plus growth hormone, 21 patients on SMT plus G-CSF, 21 patients on SMT alone	Transplant-free survival at 12 months	Not studied, but less infections after G-CSF treatment	Higher survival at 12 months	The majority of adverse effects related to G-CSF including back pain, fatigue, bone pains and fever
Spahr *et al.*, 2008[219]	5 days of G-CSF (10 µg/kg/day)	24 patients with alcohol-associated cirrhosis and alcoholic steatohepatitis; 13 patients receiving G-CSF plus SMT, 11 patients receiving only SMT	CD34 stem cell mobilisation, liver cell proliferation and liver function	Not studied. Treatment did not affect the level of circulating neutrophils, TNF-α, sTNF-R1 and IL-6 not affected; HGF level increased upon the treatment course	No improvement in liver function	Three patients complained of transient mild lower back pain reversible when G-CSF treatment ended
Gaia *et al.*, 2013[220]	3-day G-CSF 5 lg/kg every 12 h at 3-month intervals for four courses	15 patients with advanced cirrhosis	Bone marrow-derived cells mobilisation	Not studied	No effect on survival, improved Child–Pugh score	No severe adverse events, complaints about bone pain in four patients
**Indirect modulation**						
Macnaughtan *et al.*, 2020[192]	65 mL bottle of Probiotic *Lactobacillus casei* Shirota (6.5 × 10^9 colony forming units (CFU)/bottle 3 times daily for 6 months	92 patients with cirrhosis; half of the patients received probiotic, half received placebo	Incidence of significant infection and neutrophil function	Rates of infection and neutrophil function (ROS production and phagocytosis) did not change upon treatment; plasma monocyte chemo tactic protein-1 and IL-1β (in alcohol-associated cirrhosis), IL-17a and macrophage inflammatory protein-1β (in non-alcoholic cirrhosis) decreased	No significant effects	No serious adverse effects related to probiotic intervention
Horvath *et al.*, 2016[55]	Once-a-day a dose of a multispecies probiotic (*Bifidobacterium bifidum* W23, *Bifidobacterium lactis* W52, *Lactobacillus acidophilus* W37, *Lactobacillus brevis* W63, *Lactobacillus casei* W56, *Lactobacillus salivarius* W24, *Lactococcus lactis* W19 and *Lactococcus lactis* W58) 6 g, 2.5 × 10^9 CFU/g for 6 months	92 patients with cirrhosis, 45 patients treated with probiotic	Change in phagocytic capacity of neutrophils between baseline and 6 months	Increased neutrophil basal ROS production, no effect on neutrophil phagocytosis	More patients in probiotic group improved their Child–Pugh score although only non-significant improvement in MELD score	Adverse events possibly related to the probiotic (in 41% of patients treated with probiotic): flatulence, gastric pain, diarrhoea and nausea
Stadlbauer *et al.*, 2008[52]	*Lactobacillus casei* Shirota (6.5 × 10^9) 3 times daily for 4 weeks	20 patients with alcohol-associated cirrhosis, 12 patients with cirrhosis received probiotic treatment	Neutrophil ROS production, phagocytosis, toll-like-receptor expression, plasma cytokines and *ex vivo* endotoxin-stimulated cytokine production	Probiotics restored neutrophil phagocytic capacity, no effect on neutrophil ROS production	No changes	No adverse events

CFU, colony-forming unit; G-CSF, granulocyte colony stimulating factor; IL-, interleukin-; MELD, model for end-stage liver disease; ROS, reactive oxygen species; SMT, standard medical therapy; TNF-α, tumour necrosis factor-α.

Table 3. Current gaps and future research areas.

Issue	Description of the problem	Solution and future research
Standardisation	Methods to study neutrophil function vary, which leads sometimes to a "wrong" perception of conflicting findings	Developing standardised neutrophil function panel, standardisation in the description of methods and results in original and review papers
Methodology	Lack of clinically useful neutrophil function tests	Developing standardised neutrophil function panel applicable in clinical settings
Diagnosis	No widely accepted biomarker of neutrophil dysfunction	Development and validation of biomarker of neutrophil dysfunction
Clinical studies	Lack of clinical studies investigating therapies of neutrophil dysfunction in cirrhosis with neutrophil function as primary or secondary outcome	Including neutrophil function as an outcome in clinical studies (albumin, G-CSF supplementation and other potential therapeutic approaches)
Knowledge gaps	Some of the neutrophil functions are not well described in cirrhotic patients (*e.g.* NET formation, swarming), causative factors and molecular mechanisms of neutrophil dysfunction are still not fully deciphered	Further studies of neutrophil functions, causative factors and molecular mechanisms involved in neutrophil dysfunction development in cirrhosis
Multifactorial	Studying causative factors, mechanisms and treatment approaches individually might not provide all answers, as neutrophil dysfunction in cirrhosis is a multifactorial problem	Perform studies of potential causative factors and mechanisms of neutrophil dysfunction in cirrhosis in complex experimental study setups rather than each factor individually

NET, neutrophil extracellular trap.

can enhance neutrophil transendothelial migration.[31] Despite conflicting results regarding the effects of G-CSF on patients' survival and liver function, clinical evidence of the beneficial effects of G-CSF on neutrophil function in acute liver failure exist, indicating that treatment with G-CSF can increase neutrophil phagocytosis, killing and ROS production.[186] Several studies have shown that G-CSF therapy decreases the occurrence of infections in patients with cirrhosis, indicating immunomodulatory effects.[187–189] Interestingly, beta-blockers have recently shown potential to improve neutrophil phagocytic capacity in cirrhosis.[190]

Given the existing interactions between gut microbial composition and neutrophil function,[191] probiotic interventions have been examined for their indirect modulating effects on neutrophil function. However, the results of clinical trials are conflicting, indicating either effects of probiotic supplementation only on the phagocytic capacity of neutrophils,[52] or on neutrophil ROS production,[55] or no effect.[192] The origin of conflicting findings might be different sample sizes and different probiotics used for supplementation. A detailed overview of clinical studies is presented in Table 2.

Potential therapeutic strategies of neutrophil dysfunction in cirrhosis

Other potential therapeutic strategies include modulating bile acid composition via oral intake of bile acids, *e.g.* UDCA or cholic acid, or modulating gut microbial composition (as bile acids are metabolites of gut microbiota and the gut microbiome shapes serum bile acid composition[193]) via probiotic supplementation.

A range of bile acids including UDCA and obeticholic acid have already been approved for use in clinical settings, representing a potential treatment option for neutrophil dysfunction in cirrhosis given the altered serum bile acid composition in patients with cirrhosis and *in vitro* evidence of bile acid effects on neutrophil function.[62] However, to date, there have been no clinical studies on the effect of bile acid supplementation on neutrophil dysfunction either in cirrhosis or in any other diseases or healthy volunteers.

Furthermore, the normalisation of circulating bile acid composition can be achieved indirectly by modulating the composition of the gut microbiome. Different bacteria have been shown to be associated with bile acid metabolism. For example, Akkermansia abundance is affected by bile acids,[194] genus *Prevotella* is associated with plasma bile acid composition[195] and genus *Streptococcus* is involved in primary bile acid metabolism.[196,197] UDCA is a result of 7α/β-isomerisation of CDCA by gut microbiota,[193] *e.g.* by *Clostridium absonum*,[198] *Clostridium baratii*[199] and strains from genera *Eubacterium* and *Ruminococcus*.[200] Therefore, a desired decrease in toxic CDCA, which is highly elevated in sera of patients with cirrhosis, can potentially be reached via the modulation of microbial composition, *e.g.* with probiotic supplements, which aims at restoration of the balance in abundance of bacteria with 7α- and 7β-hydroxysteroid dehydrogenase activity and, thus, for epimerisation of CDCA to UDCA.[201] Besides, this might be achieved by modulating the abundance of bacteria with bile salt hydrolase activity, which is responsible for bile acid deconjugation and was previously suggested to play a role in epimerisation.[199] These bacteria include *Bacteroides ovatus*,[202] *Clostridium perfringens*,[203] *Enterococcus faecalis*[204] and different strains of *Bifidobacterium*[205] and *Lactobacillus*.[206,207] Administering a combination of bacterial strains with bile salt hydrolase activity and 7α-/7β-hydroxysteroid dehydrogenase activity in order to increase UDCA production has already been proposed.[208]

Another potential therapeutic strategy to treat neutrophil dysfunction in cirrhosis may be the inhibition of PD1 (programmed cell death 1) and TIM3 (T-cell immunoglobulin and mucin domain-containing protein 3) receptors, which mediate immunosuppression. *Ex vivo* studies have shown that the presence of antibodies blocking PD1 and TIM3 increases neutrophil antimicrobial activities, such as phagocytic capacity and ROS production, in response to *E. coli*.[209] Furthermore, strategies involving liver assist devices can potentially improve neutrophil function in cirrhosis by removing endotoxin, oxidised albumin[210] and cytokines[211] from the circulation.

Conclusion

Neutrophil dysfunction is a recognised feature of cirrhosis that predisposes patients with cirrhosis to bacterial infections and increases their mortality rate. Despite extensive studies in recent decades, significant knowledge gaps and problems remain to be solved, which are summarised in Table 3. There is a need for

standardisation of the approaches used to assess different neutrophil functions. There is also a need to develop neutrophil function screening panels that are useful for clinical studies and clinical practice as most of the currently available methodologies to study neutrophil function are extremely time- and labour-consuming and performer-sensitive due to the short lifespan and fragility of neutrophils *ex vivo*. In particular, more knowledge on recently described neutrophil behaviour, like NET formation or swarming, as well as on the molecular mechanisms involved in the development of neutrophil dysfunction, are needed. Furthermore, there is still a vague understanding of the best strategy to prevent and treat neutrophil dysfunction in cirrhosis, mostly because of the lack of clinical studies with neutrophil function as a primary or secondary outcome. So far, albumin supplementation and G-CSF have the most clinical evidence; however, although clinical benefits have been noted, a clear causal relationship between these therapies and improvement of neutrophil function needs to be established. Last but not least, it will be important to study neutrophil dysfunction in patients with cirrhosis as a multifactorial problem. Studies that investigate the potential causative factors of neutrophil dysfunction not individually but interdependently, *e.g.* the interdependent role of serum proteins, lipoproteins and bile acids, are needed. This will improve our understanding of the mechanisms behind defects in neutrophil function and guide efforts to improve the prevention and treatment of neutrophil dysfunction, decrease the development and consequences of bacterial infections and, ultimately, improve quality of life and survival in cirrhosis.

Abbreviations

ACLF, acute-on-chronic liver failure; CDCA, chenodeoxycholic acid; CXCL, CXC motif ligand; DCA, deoxycholic acid; fMLF, N-formyl-met-leu-phe; G-CSF, granulocyte colony stimulating factor; GM-CSF, granulocyte macrophage colony stimulating factor; HDL, high-density lipoprotein; IL, interleukin; LPS, lipopolysaccharide; MPO, myeloperoxidase; NETs, neutrophil extracellular traps; PBC, primary biliary cholangitis; PMA, phorbol-12-myristate-13-acetate; ROS, reactive oxygen species; TLR, Toll-like receptor; TNF-α, tumour necrosis factor alpha; UDCA, ursodeoxycholic acid.

Financial support

V.S. received funding from the Austrian Science Fund (KLI 741). The project was in part conducted at the Center for Biomarker Research in Medicine (CBmed), a COMET K1 centre funded by the Austrian Research Promotion Agency (Project 3.23), I.B.'s work was in part funded through the Doctoral College "Molecular Fundamentals of Inflammation" (W1241).

Conflicts of interest

The authors declare no conflict of interest.

Please refer to the accompanying ICMJE disclosure forms for further details.

Authors' contributions

Conceptualization, V.S.; writing—original draft preparation, I.B. and V.S; writing—review and editing, V.S. and I.B.; visualization, I.B.; supervision, V.S.; funding acquisition, V.S. All authors have read and agreed to the published version of the manuscript.

Supplementary data

Supplementary data to this article can be found online at https://doi.org/10.1016/j.jhepr.2023.100871.

References

Author names in bold designate shared co-first authorship

[1] Schuppan D, Afdhal NH. Cirrhosis. Lancet 2008;371(9615):838–851.
[2] Wiegand J, Berg T. The etiology, diagnosis and prevention of cirrhosis: part 1 of a series on cirrhosis. Dtsch Arztebl Int 2013;110(6):85–91.
[3] Global health estimates 2016: deaths by cause, age, sex, by country and by region, 2000-2016. Geneva: World Health Organisation; 2018.
[4] Collaborators GBDC. The global, regional, and national burden of cirrhosis by cause in 195 countries and territories, 1990-2017: a systematic analysis for the Global Burden of Disease Study 2017. Lancet Gastroenterol Hepatol 2020;5(3):245–266.
[5] Mokdad AA, Lopez AD, Shahraz S, Lozano R, Mokdad AH, Stanaway J, Murray CJ, Naghavi M. Cirrhosis mortality in 187 countries between 1980 and 2010: a systematic analysis. BMC Med 2014;12:145.
[6] Fleming KM, Aithal GP, Card TR, West J. All-cause mortality in people with cirrhosis compared with the general population: a population-based cohort study. Liver Int 2012;32(1):79–84.
[7] Borzio M, Salerno F, Piantoni L, Cazzaniga M, Angeli P, Bissoli F, Boccia S, Colloredo-Mels G, Corigliano P, Fornaciari G, Marenco G, Pistara R, Salvagnini M, Sangiovanni A. Bacterial infection in patients with advanced cirrhosis: a multicentre prospective study. Dig Liver Dis 2001;33(1):41–48.
[8] Arvaniti V, D'Amico G, Fede G, Manousou P, Tsochatzis E, Pleguezuelo M, Burroughs AK. Infections in patients with cirrhosis increase mortality four-fold and should be used in determining prognosis. Gastroenterology 2010;139(4):1246–1256. 1256 e1-e1256.
[9] Bajaj JS, O'Leary JG, Reddy KR, Wong F, Olson JC, Subramanian RM, Brown G, Noble NA, Thacker LR, Kamath PS, Nacseld. Second infections independently increase mortality in hospitalized patients with cirrhosis: the North American consortium for the study of end-stage liver disease (NACSELD) experience. Hepatology 2012;56(6):2328–2335.
[10] Reddy KR, O'Leary JG, Kamath PS, Fallon MB, Biggins SW, Wong F, Patton HM, Garcia-Tsao G, Subramanian RM, Thacker LR, Bajaj JS. North American consortium for the study of end-stage liver, D., high risk of delisting or death in liver transplant candidates following infections: results from the North American consortium for the study of end-stage liver disease. Liver Transpl 2015;21(7):881–888.
[11] Fasolato S, Angeli P, Dallagnese L, Maresio G, Zola E, Mazza E, Salinas F, Dona S, Fagiuoli S, Sticca A, Zanus G, Cillo U, Frasson I, Destro C, Gatta A. Renal failure and bacterial infections in patients with cirrhosis: epidemiology and clinical features. Hepatology 2007;45(1):223–229.
[12] Irvine KM, Ratnasekera I, Powell EE, Hume DA. Causes and consequences of innate immune dysfunction in cirrhosis. Front Immunol 2019;10:293.
[13] Piano S, Singh V, Caraceni P, Maiwall R, Alessandria C, Fernandez J, Soares EC, Kim DJ, Kim SE, Marino M, Vorobioff J, Barea RCR, Merli M, Elkrief L, Vargas V, Krag A, Singh SP, Lesmana LA, Toledo C, Marciano S, Verhelst X, Wong F, Intagliata N, Rabinowich L, Colombato L, Kim SG, Gerbes A, Durand F, Roblero JP, Bhamidimarri KR, Boyer TD, Maevskaya M, Fassio E, Kim HS, Hwang JS, Gines P, Gadano A, Sarin SK, Angeli P, International Club of Ascites Global Study G. Epidemiology and effects of bacterial infections in patients with cirrhosis worldwide. Gastroenterology 2019;156(5):1368–1380 e10.
[14] Fernandez J, Prado V, Trebicka J, Amoros A, Gustot T, Wiest R, Deulofeu C, Garcia E, Acevedo J, Fuhrmann V, Durand F, Sanchez C, Papp M, Caraceni P, Vargas V, Banares R, Piano S, Janicko M, Albillos A, Alessandria C, Soriano G, Welzel TM, Laleman W, Gerbes A, De Gottardi A, Merli M, Coenraad M, Saliba F, Pavesi M, Jalan R, Gines P, Angeli P, Arroyo V. European Foundation for the Study of Chronic Liver, F., Multidrug-resistant bacterial infections in patients with decompensated cirrhosis and with acute-on-chronic liver failure in Europe. J Hepatol 2019;70(3):398–411.
[15] Albillos A, Lario M, Alvarez-Mon M. Cirrhosis-associated immune dysfunction: distinctive features and clinical relevance. J Hepatol 2014;61(6):1385–1396.
[16] Bernsmeier C, van der Merwe S, Perianin A. Innate immune cells in cirrhosis. J Hepatol 2020;73(1):186–201.
[17] Biyik M, Ucar R, Solak Y, Gungor G, Polat I, Gaipov A, Cakir OO, Ataseven H, Demir A, Turk S, Polat H. Blood neutrophil-to-lymphocyte

ratio independently predicts survival in patients with cirrhosis. Eur J Gastroenterol Hepatol 2013;25(4):435–441.
[18] Weiss E, de la Grange P, Defaye M, Lozano JJ, Aguilar F, Hegde P, Jolly A, Moga L, Sukriti S, Agarwal B, Gurm H, Tanguy M, Poisson J, Claria J, Abback PS, Perianin A, Mehta G, Jalan R, Francoz C, Rautou PE, Lotersztajn S, Arroyo V, Durand F, Moreau R. Characterization of blood immune cells in patients with decompensated cirrhosis including ACLF. Front Immunol 2020;11:619039.
[19] Wu W, Yan H, Zhao H, Sun W, Yang Q, Sheng J, Shi Y. Characteristics of systemic inflammation in hepatitis B-precipitated ACLF: differentiate it from No-ACLF. Liver Int 2018;38(2):248–257.
[20] Kalra A, Wedd JP, Bambha KM, Gralla J, Golden-Mason L, Collins C, Rosen HR, Biggins SW. Neutrophil-to-lymphocyte ratio correlates with proinflammatory neutrophils and predicts death in low model for end-stage liver disease patients with cirrhosis. Liver Transpl 2017;23(2):155–165.
[21] Moreau N, Wittebole X, Fleury Y, Forget P, Laterre PF, Castanares-Zapatero D. Neutrophil-to-Lymphocyte ratio predicts death in acute-on-chronic liver failure patients admitted to the intensive care unit: a retrospective cohort study. Shock 2018;49(4):385–392.
[22] Peng Y, Li Y, He Y, Wei Q, Xie Q, Zhang L, Xia Y, Zhou X, Zhang L, Feng X, Chen K, Chen S, Chen W, Long Q, Chai J. The role of neutrophil to lymphocyte ratio for the assessment of liver fibrosis and cirrhosis: a systematic review. Expert Rev Gastroenterol Hepatol 2018;12(5):503–513.
[23] Albillos A, Martin-Mateos R, Van der Merwe S, Wiest R, Jalan R, Alvarez-Mon M. Cirrhosis-associated immune dysfunction. Nat Rev Gastroenterol Hepatol 2022;19(2):112–134.
[24] Xu R, Huang H, Zhang Z, Wang FS. The role of neutrophils in the development of liver diseases. Cell Mol Immunol 2014;11(3):224–231.
[25] Cho Y, Szabo G. Two faces of neutrophils in liver disease development and progression. Hepatology 2021;74(1):503–512.
[26] Liu K, Wang FS, Xu R. Neutrophils in liver diseases: pathogenesis and therapeutic targets. Cell Mol Immunol 2021;18(1):38–44.
[27] Ley K, Hoffman HM, Kubes P, Cassatella MA, Zychlinsky A, Hedrick CC, Catz SD. Neutrophils: new insights and open questions. Sci Immunol 2018;3(30).
[28] Nauseef WM, Borregaard N. Neutrophils at work. Nat Immunol 2014;15(7):602–611.
[29] Witko-Sarsat V, Rieu P, Descamps-Latscha B, Lesavre P, Halbwachs-Mecarelli L. Neutrophils: molecules, functions and pathophysiological aspects. Lab Invest 2000;80(5):617–653.
[30] Rajkovic IA, Yousif-Kadaru AG, Wyke RJ, Williams R. Polymorphonuclear leucocyte locomotion and aggregation in patients with alcoholic liver disease. Clin Exp Immunol 1984;58(3):654–662.
[31] Fiuza C, Salcedo M, Clemente G, Tellado JM. Granulocyte colony-stimulating factor improves deficient in vitro neutrophil transendothelial migration in patients with advanced liver disease. Clin Diagn Lab Immun 2002;9(2):433–439.
[32] Onishi S, Saibara T, Maeda T, Yamamoto Y, Ito K. Serum inhibition of complement derived leukocyte chemotaxis and levels of immunoglobulin A subclass in alcoholic liver disease. Gastroenterol Jpn 1989;24(3):284–289.
[33] Campbell AC, Dronfield MW, Toghill PJ, Reeves WG. Neutrophil function in chronic liver disease. Clin Exp Immunol 1981;45(1):81–89.
[34] Fiuza C, Salcedo M, Clemente G, Tellado JM. In vivo neutrophil dysfunction in cirrhotic patients with advanced liver disease. J Infect Dis 2000;182(2):526–533.
[35] Vanepps DE, Strickland RG, Williams RC. Inhibitors of leukocyte chemotaxis in alcoholic liver-disease. Am J Med 1975;59(2):200–207.
[36] Claria J, Titos E, Jimenez W, Ros J, Gines P, Arroyo V, Rivera F, Rodes J. Altered biosynthesis of leukotrienes and lipoxins and host defense disorders in patients with cirrhosis and ascites. Gastroenterology 1998;115(1):147–156.
[37] Lister PD, Gentry MJ, Preheim LC. Ethanol impairs neutrophil chemotaxis in vitro but not adherence or recruitment to lungs of rats with experimental pneumococcal pneumonia. J Infect Dis 1993;167(5):1131–1137.
[38] **Stadlbauer V, Horvath A**, Komarova I, Schmerboeck B, Feldbacher N, Wurm S, et al. A single alcohol binge impacts on neutrophil function without changes in gut barrier function and gut microbiome composition in healthy volunteers. PLoS One 2019;14(2):e0211703.
[39] Hallengren B, Forsgren A. Effect of alcohol on chemotaxis, adherence and phagocytosis of human polymorphonuclear leucocytes. Acta Med Scand 1978;204(1–2):43–48.
[40] Schleiffenbaum B, Moser R, Patarroyo M, Fehr J. The cell surface glycoprotein Mac-1 (CD11b/CD18) mediates neutrophil adhesion and modulates degranulation independently of its quantitative cell surface expression. J Immunol 1989;142(10):3537–3545.
[41] Zhang D, Chen G, Manwani D, Mortha A, Xu C, Faith JJ, Burk RD, Kunisaki Y, Jang JE, Scheiermann C, Merad M, Frenette PS. Neutrophil ageing is regulated by the microbiome. Nature 2015;525(7570):528–532.
[42] Artru F, Bou Saleh M, Maggiotto F, Lassailly G, Ningarhari M, Demaret J, Ntandja-Wandji LC, Pais de Barros JP, Labreuche J, Drumez E, Helou DG, Dharancy S, Gantier E, Perianin A, Chollet-Martin S, Bataller R, Mathurin P, Dubuquoy L, Louvet A. IL-33/ST2 pathway regulates neutrophil migration and predicts outcome in patients with severe alcoholic hepatitis. J Hepatol 2020;72(6):1052–1061.
[43] Langer MM, Sichelschmidt S, Bauschen A, Bornemann L, Guckenbiehl S, Gunzer M, Lange CM. Pathological neutrophil migration predicts adverse outcomes in hospitalized patients with cirrhosis. Liver Int 2023;43(4):896–905.
[44] Satsangi S, Duseja A, Sachdeva M, Tomer S, Arora SK, Taneja S, Dhiman RK, Chawla YK. Monocyte human leukocyte antigen - antigen D related, neutrophil oxidative burst and cytokine analysis in patients of decompensated cirrhosis with and without acute-on chronic liver failure. PLoS One 2018;13(7):e0200644.
[45] **Xiao L, Tang S**, Zhang L, Ma S, Zhao Y, Zhang F, Xie Z, Li L. Serum CXCL1 is a prognostic factor for patients with hepatitis B virus-related acute-on-chronic liver failure. Front Med (Lausanne) 2021;8:657076.
[46] Zimmermann HW, Seidler S, Gassler N, Nattermann J, Luedde T, Trautwein C, Tacke F. Interleukin-8 is activated in patients with chronic liver diseases and associated with hepatic macrophage accumulation in human liver fibrosis. PLoS One 2011;6(6):e21381.
[47] Chen X, Yang D, Shen W, Dong HF, Wang JM, Oppenheim JJ, Howard MZ. Characterization of chenodeoxycholic acid as an endogenous antagonist of the G-coupled formyl peptide receptors. Inflamm Res 2000;49(12):744–755.
[48] Laffi G, Carloni V, Baldi E, Rossi ME, Azzari C, Gresele P, Marra F, Gentilini P. Impaired superoxide anion, platelet-activating factor, and leukotriene B4 synthesis by neutrophils in cirrhosis. Gastroenterology 1993;105(1):170–177.
[49] Knooihuizen SAI, Alexander NJ, Hopke A, Barros N, Viens A, Scherer A, Atallah NJ, Dagher Z, Irimia D, Chung RT, Mansour MK. Loss of coordinated neutrophil responses to the human fungal pathogen, Candida albicans, in patients with cirrhosis. Hepatol Commun 2021;5(3):502–515.
[50] Rajkovic IA, Williams R. Abnormalities of neutrophil phagocytosis, intracellular killing and metabolic activity in alcoholic cirrhosis and hepatitis. Hepatology 1986;6(2):252–262.
[51] Mookerjee RP, Stadlbauer V, Lidder S, Wright GA, Hodges SJ, Davies NA, Jalan R. Neutrophil dysfunction in alcoholic hepatitis superimposed on cirrhosis is reversible and predicts the outcome. Hepatology 2007;46(3):831–840.
[52] Stadlbauer V, Mookerjee RP, Hodges S, Wright GA, Davies NA, Jalan R. Effect of probiotic treatment on deranged neutrophil function and cytokine responses in patients with compensated alcoholic cirrhosis. J Hepatol 2008;48(6):945–951.
[53] Taylor NJ, Vijay GKM, Abeles RD, Auzinger G, Bernal W, Ma Y, Wendon JA, Shawcross DL. The severity of circulating neutrophil dysfunction in patients with cirrhosis is associated with 90-day and 1-year mortality. Aliment Pharmacol Ther 2014;40(6):705–715.
[54] Leber, B.; Balázs, I.; Horvath, A.; Posch, A.; Streit, A.; Spindelböck, W.; Feldbacher, N.; Stiegler, P.; Stauber, R. E.; Rechberger, G. N.; Kollroser, M.; Sattler, W.; Nusshold, C.; Stadlbauer, V., Direct acting antiviral therapy rescues neutrophil dysfunction and reduces hemolysis in hepatitis C infection. Translational Res.
[55] Horvath A, Leber B, Schmerboeck B, Tawdrous M, Zettel G, Hartl A, Madl T, Stryeck S, Fuchs D, Lemesch S, Douschan P, Krones E, Spindelboeck W, Durchschein F, Rainer F, Zollner G, Stauber RE, Fickert P, Stiegler P, Stadlbauer V. Randomised clinical trial: the effects of a multispecies probiotic vs. placebo on innate immune function, bacterial translocation and gut permeability in patients with cirrhosis. Aliment Pharmacol Ther 2016;44(9):926–935.
[56] De Fernandez MA, Clark A, Triger DR. Neutrophil phagocytic and bactericidal function in primary biliary cirrhosis and other chronic liver diseases. Clin Exp Immunol 1987;67(3):655–661.
[57] Sehgal R, Kaur N, Maiwall R, Ramakrishna G, Maras JS, Trehanpati N. Plasma proteomic analysis identified proteins associated with faulty

neutrophils functionality in decompensated cirrhosis patients with sepsis. Cells 2022;11(11).
[58] Makkar K, Tomer S, Verma N, Rathi S, Arora SK, Taneja S, Duseja A, Chawla YK, Dhiman RK. Neutrophil dysfunction predicts 90-day survival in patients with acute on chronic liver failure: a longitudinal case-control study. JGH Open 2020;4(4):595–602.
[59] Huang CH, Jeng WJ, Ho YP, Teng W, Hsieh YC, Chen WT, Chen YC, Lin HH, Sheen IS, Lin CY. Increased EMR2 expression on neutrophils correlates with disease severity and predicts overall mortality in cirrhotic patients. Sci Rep 2016;6:38250.
[60] **Leber B**, **Balazs I**, Horvath A, Posch A, Streit A, Spindelbock W, Feldbacher N, Stiegler P, Stauber RE, Rechberger GN, Kollroser M, Sattler W, Nusshold C, Stadlbauer V. Direct acting antiviral therapy rescues neutrophil dysfunction and reduces hemolysis in hepatitis C infection. Transl Res 2021;232:103–114.
[61] Tritto G, Bechlis Z, Stadlbauer V, Davies N, Frances R, Shah N, Mookerjee RP, Such J, Jalan R. Evidence of neutrophil functional defect despite inflammation in stable cirrhosis. J Hepatol 2011;55(3):574–581.
[62] Balazs I, Horvath A, Leber B, Feldbacher N, Sattler W, Rainer F, Fauler G, Vermeren S, Stadlbauer V. Serum bile acids in cirrhosis promote neutrophil dysfunction. Clin Transl Med 2022;12(2):e735.
[63] **Wu W**, **Sun S**, Wang Y, Zhao R, Ren H, Li Z, Zhao H, Zhang Y, Sheng J, Chen Z, Shi Y. Circulating neutrophil dysfunction in HBV-related acute-on-chronic liver failure. Front Immunol 2021;12:620365.
[64] Boussif A, Rolas L, Weiss E, Bouriche H, Moreau R, Perianin A. Impaired intracellular signaling, myeloperoxidase release and bactericidal activity of neutrophils from patients with alcoholic cirrhosis. J Hepatol 2016;64(5):1041–1048.
[65] **Bruns T**, **Peter J**, Hagel S, Herrmann A, Stallmach A. The augmented neutrophil respiratory burst in response to Escherichia coli is reduced in cirrhosis during infection. Clin Exp Immunol 2011;164(3):346–356.
[66] Garfia C, Garcia-Ruiz I, Solis-Herruzo JA. Deficient phospholipase C activity in blood polimorphonuclear neutrophils from patients with cirrhosis. J Hepatol 2004;40(5):749–756.
[67] Rolas L, Makhezer N, Hadjoudj S, El-Benna J, Djerdjouri B, Elkrief L, Moreau R, Perianin A. Inhibition of mammalian target of rapamycin aggravates the respiratory burst defect of neutrophils from decompensated patients with cirrhosis. Hepatology 2013;57(3):1163–1171.
[68] Rolas L, Boussif A, Weiss E, Letteron P, Haddad O, El-Benna J, Rautou PE, Moreau R, Perianin A. NADPH oxidase depletion in neutrophils from patients with cirrhosis and restoration via toll-like receptor 7/8 activation. Gut 2018;67(8):1505–1516.
[69] Masini E, Mugnai L, Foschi M, Laffi G, Gentilini P, Mannaioni PF. Changes in the production of nitric oxide and superoxide by inflammatory cells in cirrhosis. Int Arch Allergy Immunol 1995;107(1–3):197–198.
[70] Clement MV, Pervaiz S. Intracellular superoxide and hydrogen peroxide concentrations: a critical balance that determines survival or death. Redox Rep 2001;6(4):211–214.
[71] Li PF, Dietz R, von Harsdorf R. Differential effect of hydrogen peroxide and superoxide anion on apoptosis and proliferation of vascular smooth muscle cells. Circulation 1997;96(10):3602–3609.
[72] Tranah TH, Vijay GKM, Ryan JM, Abeles RD, Middleton PK, Shawcross DL. Dysfunctional neutrophil effector organelle mobilization and microbicidal protein release in alcohol-related cirrhosis. Am J Physiol Gastrointest Liver Physiol 2017;313(3):G203–G211.
[73] Brinkmann V, Reichard U, Goosmann C, Fauler B, Uhlemann Y, Weiss DS, Weinrauch Y, Zychlinsky A. Neutrophil extracellular traps kill bacteria. Science 2004;303(5663):1532–1535.
[74] Agraz-Cibrian JM, Segura-Ortega JE, Delgado-Rizo V, Fafutis-Morris M. Alterations in neutrophil extracellular traps is associated with the degree of decompensation of cirrhosis. J Infect Dev Ctries 2016;10(5):512–517.
[75] Agraz-Cibrian JM, Delgado-Rizo V, Segura-Ortega JE, Maldonado-Gomez HA, Zambrano-Zaragoza JF, Duran-Avelar MJ, Vibanco-Perez N, Fafutis-Morris M. Impaired neutrophil extracellular traps and inflammatory responses in the peritoneal fluid of patients with cirrhosis. Scand J Immunol 2018;88(5):e12714.
[76] Zenlander R, Havervall S, Magnusson M, Engstrand J, Agren A, Thalin C, Stal P. Neutrophil extracellular traps in patients with cirrhosis and hepatocellular carcinoma. Sci Rep 2021;11(1):18025.
[77] **Blasi A**, **Patel VC**, Adelmeijer J, Azarian S, Aziz F, Fernandez J, Bernal W, Lisman T. Plasma levels of circulating DNA are associated with outcome, but not with activation of coagulation in decompensated cirrhosis and ACLF. JHEP Rep 2019;1(3):179–187.
[78] Kusaba N, Kumashiro R, Ogata H, Sata M, Tanikawa K. In vitro study of neutrophil apoptosis in cirrhosis. Intern Med 1998;37(1):11–17.
[79] Ramirez MJ, Titos E, Claria J, Navasa M, Fernandez J, Rodes J. Increased apoptosis dependent on caspase-3 activity in polymorphonuclear leukocytes from patients with cirrhosis and ascites. J Hepatol 2004;41(1):44–48.
[80] Hempel SL, Buettner GR, O'Malley YQ, Wessels DA, Flaherty DM. Dihydrofluorescein diacetate is superior for detecting intracellular oxidants: comparison with 2',7'-dichlorodihydrofluorescein diacetate, 5(and 6)-carboxy-2',7'-dichlorodihydrofluorescein diacetate, and dihydrorhodamine 123. Free Radic Biol Med 1999;27(1–2):146–159.
[81] Dikalov S, Griendling KK, Harrison DG. Measurement of reactive oxygen species in cardiovascular studies. Hypertension 2007;49(4):717–727.
[82] Rajecky M, Lojek A, Ciz M. Differentiating between intra- and extracellular chemiluminescence in diluted whole-blood samples. Int J Lab Hematol 2012;34(2):136–142.
[83] **Xie X**, **Shi Q**, Wu P, Zhang X, Kambara H, Su J, Yu H, Park SY, Guo R, Ren Q, Zhang S, Xu Y, Silberstein LE, Cheng T, Ma F, Li C, Luo HR. Single-cell transcriptome profiling reveals neutrophil heterogeneity in homeostasis and infection. Nat Immunol 2020;21(9):1119–1133.
[84] **Huang J**, **Zhu Z**, **Ji D**, Sun R, Yang Y, Liu L, Shao Y, Chen Y, Li L, Sun B. Single-cell transcriptome profiling reveals neutrophil heterogeneity and functional multiplicity in the early stage of severe burn patients. Front Immunol 2021;12:792122.
[85] Hilscher MB, Sehrawat T, Arab JP, Zeng Z, Gao J, Liu M, Kostallari E, Gao Y, Simonetto DA, Yaqoob U, Cao S, Revzin A, Beyder A, Wang RA, Kamath PS, Kubes P, Shah VH. Mechanical stretch increases expression of CXCL1 in liver sinusoidal endothelial cells to recruit neutrophils, generate sinusoidal microthombi, and promote portal hypertension. Gastroenterology 2019;157(1):193–209 e9.
[86] Vairappan B. Endothelial dysfunction in cirrhosis: role of inflammation and oxidative stress. World J Hepatol 2015;7(3):443–459.
[87] Hassouna MM, Mostafa MS, Omar HM, Mohammed EA, Abdallah HM. Platelet transfusion in patients with cirrhosis cross-talk with neutrophil: prospective study. Hematol Transfus Cell Ther 2022. S2531-1379(22)01424-9.
[88] Sadiku P, Willson JA, Ryan EM, Sammut D, Coelho P, Watts ER, Grecian R, Young JM, Bewley M, Arienti S, Mirchandani AS, Sanchez Garcia MA, Morrison T, Zhang A, Reyes L, Griessler T, Jheeta P, Paterson GG, Graham CJ, Thomson JP, Baillie K, Thompson AAR, Morgan JM, Acosta-Sanchez A, Darde VM, Duran J, Guinovart JJ, Rodriguez-Blanco G, Von Kriegsheim A, Meehan RR, Mazzone M, Dockrell DH, Ghesquiere B, Carmeliet P, Whyte MKB, Walmsley SR. Neutrophils fuel effective immune responses through gluconeogenesis and glycogenesis. Cell Metab 2021;33(5):1062–1064.
[89] Tsiaoussis GI, Assimakopoulos SF, Tsamandas AC, Triantos CK, Thomopoulos KC. Intestinal barrier dysfunction in cirrhosis: current concepts in pathophysiology and clinical implications. World J Hepatol 2015;7(17):2058–2068.
[90] Stadlbauer V, Mookerjee RP, Wright GA, Davies NA, Jurgens G, Hallstrom S, Jalan R. Role of Toll-like receptors 2, 4, and 9 in mediating neutrophil dysfunction in alcoholic hepatitis. Am J Physiol Gastrointest Liver Physiol 2009;296(1):G15–G22.
[91] Albillos A, de-la-Hera A, Alvarez-Mon M. Serum lipopolysaccharide-binding protein prediction of severe bacterial infection in cirrhotic patients with ascites. Lancet 2004;363(9421):1608–1610.
[92] Dentener MA, Von Asmuth EJ, Francot GJ, Marra MN, Buurman WA. Antagonistic effects of lipopolysaccharide binding protein and bactericidal/permeability-increasing protein on lipopolysaccharide-induced cytokine release by mononuclear phagocytes. Competition for binding to lipopolysaccharide. J Immunol 1993;151(8):4258–4265.
[93] Such J, Frances R, Munoz C, Zapater P, Casellas JA, Cifuentes A, Rodriguez-Valera F, Pascual S, Sola-Vera J, Carnicer F, Uceda F, Palazon JM, Perez-Mateo M. Detection and identification of bacterial DNA in patients with cirrhosis and culture-negative, nonneutrocytic ascites. Hepatology 2002;36(1):135–141.
[94] Frances R, Benlloch S, Zapater P, Gonzalez JM, Lozano B, Munoz C, Pascual S, Casellas JA, Uceda F, Palazon JM, Carnicer F, Perez-Mateo M, Such J. A sequential study of serum bacterial DNA in patients with advanced cirrhosis and ascites. Hepatology 2004;39(2):484–491.
[95] Frances R, Zapater P, Gonzalez-Navajas JM, Munoz C, Cano R, Moreu R, Pascual S, Bellot P, Perez-Mateo M, Such J. Bacterial DNA in patients with cirrhosis and noninfected ascites mimics the soluble immune response

established in patients with spontaneous bacterial peritonitis. Hepatology 2008;47(3):978–985.
[96] Gonzalez-Navajas JM, Bellot P, Frances R, Zapater P, Munoz C, Garcia-Pagan JC, Pascual S, Perez-Mateo M, Bosch J, Such J. Presence of bacterial-DNA in cirrhosis identifies a subgroup of patients with marked inflammatory response not related to endotoxin. J Hepatol 2008;48(1):61–67.
[97] Spinella R, Sawhney R, Jalan R. Albumin in chronic liver disease: structure, functions and therapeutic implications. Hepatol Int 2016;10(1):124–132.
[98] Oettl K, Stadlbauer V, Petter F, Greilberger J, Putz-Bankuti C, Hallstrom S, Lackner C, Stauber RE. Oxidative damage of albumin in advanced liver disease. Bba-mol Basis Dis 2008;1782(7–8):469–473.
[99] Claria J, Stauber RE, Coenraad MJ, Moreau R, Jalan R, Pavesi M, Amoros A, Titos E, Alcaraz-Quiles J, Oettl K, Morales-Ruiz M, Angeli P, Domenicali M, Alessandria C, Gerbes A, Wendon J, Nevens F, Trebicka J, Laleman W, Saliba F, Welzel TM, Albillos A, Gustot T, Benten D, Durand F, Gines P, Bernardi M, Arroyo V, Consortium, C. S. I. o. t. E.-C.; the European Foundation for the Study of Chronic Liver, F.. Systemic inflammation in decompensated cirrhosis: characterization and role in acute-on-chronic liver failure. Hepatology 2016;64(4):1249–1264.
[100] Klammt S, Mitzner S, Stange J, Brinkmann B, Drewelow B, Emmrich J, Liebe S, Schmidt R. Albumin-binding function is reduced in patients with decompensated cirrhosis and correlates inversely with severity of liver disease assessed by model for end-stage liver disease. Eur J Gastroenterol Hepatol 2007;19(3):257–263.
[101] Jalan R, Schnurr K, Mookerjee RP, Sen S, Cheshire L, Hodges S, Muravsky V, Williams R, Matthes G, Davies NA. Alterations in the functional capacity of albumin in patients with decompensated cirrhosis is associated with increased mortality. Hepatology 2009;50(2):555–564.
[102] Han Z, He X, Peng S. Neutrophil count to albumin ratio as a prognostic indicator for HBV-associated decompensated cirrhosis. J Clin Lab Anal 2021;35(4):e23730.
[103] **Du X**, **Wei X**, Ma L, Liu X, Guo H, Liu Y, Zhang J. Higher levels of neutrophil percentage-to-albumin ratio predict increased mortality risk in patients with cirrhosis: a retrospective cohort study. Eur J Gastroenterol Hepatol 2023;35(2):198–203.
[104] Arroyo V, Garcia-Martinez R, Salvatella X. Human serum albumin, systemic inflammation, and cirrhosis. J Hepatol 2014;61(2):396–407.
[105] Jaisson S, Delevallee-Forte C, Toure F, Rieu P, Garnotel R, Gillery P. Carbamylated albumin is a potent inhibitor of polymorphonuclear neutrophil respiratory burst. FEBS Lett 2007;581(7):1509–1513.
[106] Shalimar, Sheikh MF, Mookerjee RP, Agarwal B, Acharya SK, Jalan R. Prognostic role of ammonia in patients with cirrhosis. Hepatology 2019;70(3):982–994.
[107] Shawcross DL, Wright GA, Stadlbauer V, Hodges SJ, Davies NA, Wheeler-Jones C, Pitsillides AA, Jalan R. Ammonia impairs neutrophil phagocytic function in liver disease. Hepatology 2008;48(4):1202–1212.
[108] **Trieb M**, **Rainer F**, Stadlbauer V, Douschan P, Horvath A, Binder L, Trakaki A, Knuplez E, Scharnagl H, Stojakovic T, Heinemann A, Mandorfer M, Paternostro R, Reiberger T, Pitarch C, Amoros A, Gerbes A, Caraceni P, Alessandria C, Moreau R, Claria J, Marsche G, Stauber RE. HDL-related biomarkers are robust predictors of survival in patients with chronic liver failure. J Hepatol 2020;73(1):113–120.
[109] Levine DM, Parker TS, Donnelly TM, Walsh A, Rubin AL. In vivo protection against endotoxin by plasma high density lipoprotein. Proc Natl Acad Sci U S A 1993;90(24):12040–12044.
[110] Trieb M, Horvath A, Birner-Gruenberger R, Spindelboeck W, Stadlbauer V, Taschler U, Curcic S, Stauber RE, Holzer M, Pasterk L, Heinemann A, Marsche G. Liver disease alters high-density lipoprotein composition, metabolism and function. Biochim Biophys Acta 2016;1861(7):630–638.
[111] Wewers MD, Rinehart JJ, She ZW, Herzyk DJ, Hummel MM, Kinney PA, Davis WB. Tumor necrosis factor infusions in humans prime neutrophils for hypochlorous acid production. Am J Physiol 1990;259(4 Pt 1):L276–L282.
[112] Laichalk LL, Danforth JM, Standiford TJ. Interleukin-10 inhibits neutrophil phagocytic and bactericidal activity. FEMS Immunol Med Microbiol 1996;15(4):181–187.
[113] Fielding CA, McLoughlin RM, McLeod L, Colmont CS, Najdovska M, Grail D, Ernst M, Jones SA, Topley N, Jenkins BJ. IL-6 regulates neutrophil trafficking during acute inflammation via STAT3. J Immunol 2008;181(3):2189–2195.
[114] Florentin J, Zhao J, Tai YY, Vasamsetti SB, O'Neil SP, Kumar R, Arunkumar A, Watson A, Sembrat J, Bullock GC, Sanders L, Kassa B, Rojas M, Graham BB, Chan SY, Dutta P. Interleukin-6 mediates neutrophil mobilization from bone marrow in pulmonary hypertension. Cell Mol Immunol 2021;18(2):374–384.
[115] Kaiser R, Leunig A, Pekayvaz K, Popp O, Joppich M, Polewka V, Escaig R, Anjum A, Hoffknecht ML, Gold C, Brambs S, Engel A, Stockhausen S, Knottenberg V, Titova A, Haji M, Scherer C, Muenchhoff M, Hellmuth JC, Saar K, Schubert B, Hilgendorff A, Schulz C, Kaab S, Zimmer R, Hubner N, Massberg S, Mertins P, Nicolai L, Stark K. Self-sustaining IL-8 loops drive a prothrombotic neutrophil phenotype in severe COVID-19. JCI Insight 2021;6(18).
[116] Wright HL, Cross AL, Edwards SW, Moots RJ. Effects of IL-6 and IL-6 blockade on neutrophil function in vitro and in vivo. Rheumatology (Oxford) 2014;53(7):1321–1331.
[117] Prince LR, Allen L, Jones EC, Hellewell PG, Dower SK, Whyte MK, Sabroe I. The role of interleukin-1beta in direct and toll-like receptor 4-mediated neutrophil activation and survival. Am J Pathol 2004;165(5):1819–1826.
[118] Meher AK, Spinosa M, Davis JP, Pope N, Laubach VE, Su G, Serbulea V, Leitinger N, Ailawadi G, Upchurch Jr GR. Novel role of IL (Interleukin)-1beta in neutrophil extracellular trap formation and abdominal aortic aneurysms. Arterioscler Thromb Vasc Biol 2018;38(4):843–853.
[119] Liu R, Lauridsen HM, Amezquita RA, Pierce RW, Jane-Wit D, Fang C, Pellowe AS, Kirkiles-Smith NC, Gonzalez AL, Pober JS. IL-17 promotes neutrophil-mediated immunity by activating microvascular pericytes and not endothelium. J Immunol 2016;197(6):2400–2408.
[120] Homann C, Garred P, Graudal N, Hasselqvist P, Christiansen M, Fagerhol MK, Thomsen AC. Plasma calprotectin: a new prognostic marker of survival in alcohol-induced cirrhosis. Hepatology 1995;21(4):979–985.
[121] Matiollo C, Rateke ECM, Moura EQA, Andrigueti M, de Augustinho FC, Zocche TL, Silva TE, Gomes LO, Farias MR, Narciso-Schiavon JL, Schiavon LL. Elevated calprotectin levels are associated with mortality in patients with acute decompensation of cirrhosis. World J Hepatol 2022;14(11):1964–1976.
[122] Milic S, Mikolasevic I, Orlic L, Devcic E, Starcevic-Cizmarevic N, Stimac D, Kapovic M, Ristic S. The role of iron and iron overload in chronic liver disease. Med Sci Monit 2016;22:2144–2151.
[123] Martins R, Knapp S. Heme and hemolysis in innate immunity: adding insult to injury. Curr Opin Immunol 2018;50:14–20.
[124] Schaer DJ, Buehler PW, Alayash AI, Belcher JD, Vercellotti GM. Hemolysis and free hemoglobin revisited: exploring hemoglobin and hemin scavengers as a novel class of therapeutic proteins. Blood 2013;121(8):1276–1284.
[125] Arruda MA, Rossi AG, de Freitas MS, Barja-Fidalgo C, Graca-Souza AV. Heme inhibits human neutrophil apoptosis: involvement of phosphoinositide 3-kinase, MAPK, and NF-kappaB. J Immunol 2004;173(3):2023–2030.
[126] Porto BN, Alves LS, Fernandez PL, Dutra TP, Figueiredo RT, Graca-Souza AV, Bozza MT. Heme induces neutrophil migration and reactive oxygen species generation through signaling pathways characteristic of chemotactic receptors. J Biol Chem 2007;282(33):24430–24436.
[127] Martins R, Maier J, Gorki AD, Huber KV, Sharif O, Starkl P, Saluzzo S, Quattrone F, Gawish R, Lakovits K, Aichinger MC, Radic-Sarikas B, Lardeau CH, Hladik A, Korosec A, Brown M, Vaahtomeri K, Duggan M, Kerjaschki D, Esterbauer H, Colinge J, Eisenbarth SC, Decker T, Bennett KL, Kubicek S, Sixt M, Superti-Furga G, Knapp S. Heme drives hemolysis-induced susceptibility to infection via disruption of phagocyte functions. Nat Immunol 2016;17(12):1361–1372.
[128] Graca-Souza AV, Arruda MA, de Freitas MS, Barja-Fidalgo C, Oliveira PL. Neutrophil activation by heme: implications for inflammatory processes. Blood 2002;99(11):4160–4165.
[129] Cantinieaux B, Janssens A, Boelaert JR, Lejeune M, Vermylen C, Kerrels V, Cornu G, Winand J, Fondu P. Ferritin-associated iron induces neutrophil dysfunction in hemosiderosis. J Lab Clin Med 1999;133(4):353–361.
[130] Park W, Jang DH, Kim SH, Han CS, Shin WS, Kim HY, Kim DJ. Impaired polymorphonuclear leukocyte function in chronically hemodialyzed patients with iron overload. Korean J Intern Med 1988;3(1):64–71.
[131] Orino K, Lehman L, Tsuji Y, Ayaki H, Torti SV, Torti FM. Ferritin and the response to oxidative stress. Biochem J 2001;357(Pt 1):241–247.
[132] Tornai D, Antal-Szalmas P, Tornai T, Papp M, Tornai I, Sipeki N, Janka T, Balogh B, Vitalis Z. Abnormal ferritin levels predict development of poor

outcomes in cirrhotic outpatients: a cohort study. BMC Gastroenterol 2021;21(1):94.
[133] Renassia C, Louis S, Cuvellier S, Boussetta N, Deschemin JC, Borderie D, Bailly K, Poupon J, Dang PM, El-Benna J, Manceau S, Lefrere F, Vaulont S, Peyssonnaux C. Neutrophils from hereditary hemochromatosis patients are protected from iron excess and are primed. Blood Adv 2020;4(16):3853–3863.
[134] Kuzmicka W, Manda-Handzlik A, Mroczek A, Cieloch A, Moskalik A, Demkow U, Wachowska M, Ciepiela O. Iron excess affects release of neutrophil extracellular traps and reactive oxygen species but does not influence other functions of neutrophils. Immunol Cell Biol 2022;100(2):87–100.
[135] van Kessel KP, Bestebroer J, van Strijp JA. Neutrophil-Mediated phagocytosis of Staphylococcus aureus. Front Immunol 2014;5:467.
[136] Wyke RJ, Rajkovic IA, Williams R. Impaired opsonization by serum from patients with chronic liver disease. Clin Exp Immunol 1983;51(1): 91–98.
[137] Ono Y, Watanabe T, Matsumoto K, Ito T, Kunii O, Goldstein E. Opsonophagocytic dysfunction in patients with cirrhosis and low responses to tumor necrosis factor-alpha and lipopolysaccharide in patients' blood. J Infect Chemother 2004;10(4):200–207.
[138] Algorri M, Wong-Beringer A. Differential effects of antibiotics on neutrophils exposed to lipoteichoic acid derived from Staphylococcus aureus. Ann Clin Microbiol Antimicrob 2020;19(1):50.
[139] Ettel P, Sehgal ANA, Harrison N, Pickl WF, Grabmeier-Pfistershammer K. Glycopeptide antibiotics impair neutrophil effector functions. Int Arch Allergy Immunol 2023;1:932–948.
[140] Bystrzycka W, Manda-Handzlik A, Sieczkowska S, Moskalik A, Demkow U, Ciepiela O. Azithromycin and chloramphenicol diminish neutrophil extracellular traps (NETs) release. Int J Mol Sci 2017;18(12).
[141] Bongers S, Hellebrekers P, Leenen LPH, Koenderman L, Hietbrink F. Intracellular penetration and effects of antibiotics on Staphylococcus aureus inside human neutrophils: a comprehensive review. Antibiotics (Basel) 2019;8(2).
[142] Labro MT. Interference of antibacterial agents with phagocyte functions: immunomodulation or "immuno-fairy tales"? Clin Microbiol Rev 2000;13(4):615–650.
[143] Gemmell CG. Antibiotics and neutrophil function–potential immunomodulating activities. J Antimicrob Chemother 1993;31(Suppl B):23–33.
[144] Rudman D, Kendall FE. Bile acid content of human serum. II. The binding of cholanic acids by human plasma proteins. J Clin Invest 1957;36(4):538–542.
[145] Kramer W, Buscher HP, Gerok W, Kurz G. Bile salt binding to serum components. Taurocholate incorporation into high-density lipoprotein revealed by photoaffinity labelling. Eur J Biochem 1979;102(1):1–9.
[146] Ceryak S, Bouscarel B, Fromm H. Comparative binding of bile acids to serum lipoproteins and albumin. J Lipid Res 1993;34(10):1661–1674.
[147] Neale G, Lewis B, Weaver V, Panveliwalla D. Serum bile acids in liver disease. Gut 1971;12(2):145–152.
[148] Sadaruddin A, Zuberi SJ. Total serum bile acids in liver disease. JPMA J Pakistan Med Assoc 1981;31(4):82–84.
[149] Luo L, Aubrecht J, Li D, Warner RL, Johnson KJ, Kenny J, Colangelo JL. Assessment of serum bile acid profiles as biomarkers of liver injury and liver disease in humans. PloS one 2018;13(3):e0193824.
[150] Greco AV, Mingrone G. Serum bile acid concentrations in mild cirrhosis. Clin Chim Acta 1993;221(1–2):183–189.
[151] Ferslew BC, Xie G, Johnston CK, Su M, Stewart PW, Jia W, Brouwer KL, Barritt AST. Altered bile acid metabolome in patients with nonalcoholic steatohepatitis. Dig Dis Sci 2015;60(11):3318–3328.
[152] Amuro Y, Endo T, Higashino K, Uchida K, Yamamura Y. Serum, fecal and urinary bile acids in patients with mild and advanced cirrhosis. Gastroenterol Jpn 1981;16(5):506–513.
[153] Liu N, Feng J, Lv Y, Liu Q, Deng J, Xia Y, Guo C, Zhou Y. Role of bile acids in the diagnosis and progression of cirrhosis: a prospective observational study. Exp Ther Med 2019;18(5):4058–4066.
[154] Parks DJ, Blanchard SG, Bledsoe RK, Chandra G, Consler TG, Kliewer SA, Stimmel JB, Willson TM, Zavacki AM, Moore DD, Lehmann JM. Bile acids: natural ligands for an orphan nuclear receptor. Science 1999;284(5418):1365–1368.
[155] Makishima M, Okamoto AY, Repa JJ, Tu H, Learned RM, Luk A, Hull MV, Lustig KD, Mangelsdorf DJ, Shan B. Identification of a nuclear receptor for bile acids. Science 1999;284(5418):1362–1365.
[156] Wang H, Chen J, Hollister K, Sowers LC, Forman BM. Endogenous bile acids are ligands for the nuclear receptor FXR/BAR. Mol Cell 1999;3(5):543–553.
[157] Kawamata Y, Fujii R, Hosoya M, Harada M, Yoshida H, Miwa M, Fukusumi S, Habata Y, Itoh T, Shintani Y, Hinuma S, Fujisawa Y, Fujino M. A G protein-coupled receptor responsive to bile acids. J Biol Chem 2003;278(11):9435–9440.
[158] Maruyama T, Miyamoto Y, Nakamura T, Tamai Y, Okada H, Sugiyama E, Nakamura T, Itadani H, Tanaka K. Identification of membrane-type receptor for bile acids (M-BAR). Biochem Biophys Res Commun 2002;298(5):714–719.
[159] Chavez-Talavera O, Tailleux A, Lefebvre P, Staels B. Bile acid control of metabolism and inflammation in obesity, type 2 diabetes, dyslipidemia, and nonalcoholic fatty liver disease. Gastroenterology 2017;152(7):1679–1694 e3.
[160] Gruner N, Mattner J. Bile acids and microbiota: multifaceted and versatile regulators of the liver-gut Axis. Int J Mol Sci 2021;22(3).
[161] Chiang JYL, Ferrell JM. Bile acid metabolism in liver pathobiology. Gene Expr 2018;18(2):71–87.
[162] Levy R, Schlaeffer F, Keynan A, Nagauker O, Yaari A, Sikuler E. Increased neutrophil function induced by bile duct ligation in a rat model. Hepatology 1993;17(5):908–914.
[163] Tjandra K, Woodman RC, Swain MG. Impaired neutrophil microbicidal activity in rat cholestasis. Gastroenterology 1997;112(5):1692–1698.
[164] Swain MG, Tjandra K, Kanwar S, Kubes P. Neutrophil adhesion is impaired in a rat model of cholestasis. Gastroenterology 1995;109(3):923–932.
[165] Gujral JS, Farhood A, Bajt ML, Jaeschke H. Neutrophils aggravate acute liver injury during obstructive cholestasis in bile duct-ligated mice. Hepatology 2003;38(2):355–363.
[166] Dahm LJ, Hewett JA, Roth RA. Bile and bile salts potentiate superoxide anion release from activated, rat peritoneal neutrophils. Toxicol Appl Pharmacol 1988;95(1):82–92.
[167] Dahm LJ, Roth RA. Differential-effects of lithocholate on rat neutrophil activation. J Leukoc Biol 1990;47(6):551–560.
[168] Li J, Dawson PA. Animal models to study bile acid metabolism. Biochim Biophys Acta Mol Basis Dis 2019;1865(5):895–911.
[169] Thakare R, Alamoudi JA, Gautam N, Rodrigues AD, Alnouti Y. Species differences in bile acids I. Plasma and urine bile acid composition. J Appl Toxicol 2018;38(10):1323–1335.
[170] Thakare R, Alamoudi JA, Gautam N, Rodrigues AD, Alnouti Y. Species differences in bile acids II. Bile acid metabolism. J Appl Toxicol 2018;38(10):1336–1352.
[171] Ohshio G, Miyachi Y, Kudo H, Niwa Y, Manabe T, Tobe T. Effects of sera from patients with obstructive jaundice on the generation of oxygen intermediates by normal polymorphonuclear leukocytes. Liver 1988;8(6):366–371.
[172] Santoro P, Raimondi F, Annunziata S, Paludetto R, Annella T, Ciccimarra F. Unconjugated bile acids modulate adult and neonatal neutrophil chemotaxis induced in vitro by N-formyl-met-leu-phe-peptide. Pediatr Res 2002;51(3):392–396.
[173] Chen X, Mellon RD, Yang L, Dong H, Oppenheim JJ, Howard OM. Regulatory effects of deoxycholic acid, a component of the anti-inflammatory traditional Chinese medicine Niuhuang, on human leukocyte response to chemoattractants. Biochem Pharmacol 2002;63(3):533–541.
[174] Fernandez J, Angeli P, Trebicka J, Merli M, Gustot T, Alessandria C, et al. Efficacy of albumin treatment for patients with cirrhosis and infections unrelated to spontaneous bacterial peritonitis. Clin Gastroenterol Hepatol 2019;18(4):963–973.e14.
[175] Roda A, Cappelleri G, Aldini R, Roda E, Barbara L. Quantitative aspects of the interaction of bile acids with human serum albumin. J Lipid Res 1982;23(3):490–495.
[176] Takikawa H, Sugiyama Y, Hanano M, Kurita M, Yoshida H, Sugimoto T. A novel binding site for bile acids on human serum albumin. Biochim Biophys Acta 1987;926(2):145–153.
[177] Bernardi M, Angeli P, Claria J, Moreau R, Gines P, Jalan R, Caraceni P, Fernandez J, Gerbes AL, O'Brien AJ, Trebicka J, Thevenot T, Arroyo V. Albumin in decompensated cirrhosis: new concepts and perspectives. Gut 2020;69(6):1127–1138.
[178] Papp M, Sipeki N, Vitalis Z, Tornai T, Altorjay I, Tornai I, Udvardy M, Fechner K, Jacobsen S, Teegen B, Sumegi A, Veres G, Lakatos PL, Kappelmayer J, Antal-Szalmas P. High prevalence of IgA class antineutrophil cytoplasmic antibodies (ANCA) is associated with increased risk of bacterial infection in patients with cirrhosis. J Hepatol 2013;59(3):457–466.
[179] Nakazawa D, Shida H, Tomaru U, Yoshida M, Nishio S, Atsumi T, Ishizu A. Enhanced formation and disordered regulation of NETs in myeloperoxidase-ANCA-associated microscopic polyangiitis. J Am Soc Nephrol 2014;25(5):990–997.

[180] Kessenbrock K, Krumbholz M, Schonermarck U, Back W, Gross WL, Werb Z, Grone HJ, Brinkmann V, Jenne DE. Netting neutrophils in autoimmune small-vessel vasculitis. Nat Med 2009;15(6):623–625.
[181] Caraceni P, Riggio O, Angeli P, Alessandria C, Neri S, Foschi FG, Levantesi F, Airoldi A, Boccia S, Svegliati-Baroni G, Fagiuoli S, Romanelli RG, Cozzolongo R, Di Marco V, Sangiovanni V, Morisco F, Toniutto P, Tortora A, De Marco R, Angelico M, Cacciola I, Elia G, Federico A, Massironi S, Guarisco R, Galioto A, Ballardini G, Rendina M, Nardelli S, Piano S, Elia C, Prestianni L, Cappa FM, Cesarini L, Simone L, Pasquale C, Cavallin M, Andrealli A, Fidone F, Ruggeri M, Roncadori A, Baldassarre M, Tufoni M, Zaccherini G, Bernardi M, Investigators AS. Long-term albumin administration in decompensated cirrhosis (ANSWER): an open-label randomised trial. Lancet 2018;391(10138): 2417–2429.
[182] Chen TA, Tsao YC, Chen A, Lo GH, Lin CK, Yu HC, Cheng LC, Hsu PI, Tsai WL. Effect of intravenous albumin on endotoxin removal, cytokines, and nitric oxide production in patients with cirrhosis and spontaneous bacterial peritonitis. Scand J Gastroenterol 2009;44(5):619–625.
[183] **Fernandez J**, **Claria J**, Amoros A, Aguilar F, Castro M, Casulleras M, Acevedo J, Duran-Guell M, Nunez L, Costa M, Torres M, Horrillo R, Ruiz-Del-Arbol L, Villanueva C, Prado V, Arteaga M, Trebicka J, Angeli P, Merli M, Alessandria C, Aagaard NK, Soriano G, Durand F, Gerbes A, Gustot T, Welzel TM, Salerno F, Banares R, Vargas V, Albillos A, Silva A, Morales-Ruiz M, Carlos Garcia-Pagan J, Pavesi M, Jalan R, Bernardi M, Moreau R, Paez A, Arroyo V. Effects of albumin treatment on systemic and portal hemodynamics and systemic inflammation in patients with decompensated cirrhosis. Gastroenterology 2019;157(1):149–162.
[184] Castro-Narro G, Moctezuma-Velazquez C, Male-Velazquez R, Trejo-Estrada R, Bosques FJ, Moreno-Alcantar R, Rodriguez-Hernandez H, Bautista-Santos A, Cortez-Hernandez C, Cerda-Reyes E, Perez-Escobar J, Aldana-Ledesma JM, Aguirre-Valadez J, Ruiz-Velasco JAV, Contreras-Omana R, Miranda-Zazueta G, Reyes-Bastidas MDR, Meza-Cardona JM, Chavez-Tapia N, Fernandez-Perez NJ, Garcia-Jimenez ES, Torre A. Position statement on the use of albumin in cirrhosis. Ann Hepatol 2022;27(4):100708.
[185] Agarwal B, Salba F, Tomescu DR, Canizares RB, Martin D, Stadlbauer V, et al. P076 A multi-centre, randomized controlled study, to evaluate the safety and performance of the DIALIVE liver dialysis device in patients with acute on chronic liver failure (ACLF) versus standard of care (SOC) (ALIVER Consortium). Gut 2021;70(Suppl 3):A54.
[186] Rolando N, Clapperton M, Wade J, Wendon J. Administering granulocyte colony-stimulating factor to acute liver failure patients corrects neutrophil defects. Eur J Gastroenterol Hepatol 2000;12(12):1323–1328.
[187] Venkitaraman A, De A, Verma N, Kumari S, Leishangthem B, Sharma RR, Kalra N, Grover S, Singh V. Multiple cycles of granulocyte colony-stimulating factor in decompensated cirrhosis: a double-blind RCT. Hepatol Int 2022;16(5):1127–1136.
[188] De A, Kumari S, Singh A, Kaur A, Sharma R, Bhalla A, Sharma N, Kalra N, Singh V. Multiple cycles of granulocyte colony-stimulating factor increase survival times of patients with decompensated cirrhosis in a randomized trial. Clin Gastroenterol Hepatol 2021;19(2):375–383 e5.
[189] Verma N, Kaur A, Sharma R, Bhalla A, Sharma N, De A, Singh V. Outcomes after multiple courses of granulocyte colony-stimulating factor and growth hormone in decompensated cirrhosis: a randomized trial. Hepatology 2018;68(4):1559–1573.
[190] Gimenez P, Garcia-Martinez I, Frances R, Gonzalez-Navajas JM, Mauri M, Alfayate R, Almenara S, Miralles C, Palazon JM, Carnicer F, Pascual S, Such J, Horga JF, Zapater P. Treatment with non-selective beta-blockers affects the systemic inflammatory response to bacterial DNA in patients with cirrhosis. Liver Int 2018;38(12):2219–2227.
[191] Zhang D, Frenette PS. Cross talk between neutrophils and the microbiota. Blood 2019;133(20):2168–2177.
[192] Macnaughtan J, Figorilli F, Garcia-Lopez E, Lu H, Jones H, Sawhney R, Suzuki K, Fairclough S, Marsden J, Moratella A, Cox IJ, Thomas L, Davies N, Williams R, Mookerjee R, Wright G, Jalan R. A double-blind, randomized placebo-controlled trial of probiotic Lactobacillus casei shirota in stable cirrhotic patients. Nutrients 2020;12(6).
[193] Wahlstrom A, Sayin SI, Marschall HU, Backhed F. Intestinal crosstalk between bile acids and microbiota and its impact on host metabolism. Cell Metab 2016;24(1):41–50.
[194] Hagi T, Geerlings SY, Nijsse B, Belzer C. The effect of bile acids on the growth and global gene expression profiles in Akkermansia muciniphila. Appl Microbiol Biotechnol 2020;104(24):10641–10653.
[195] Gu Y, Wang X, Li J, Zhang Y, Zhong H, Liu R, Zhang D, Feng Q, Xie X, Hong J, Ren H, Liu W, Ma J, Su Q, Zhang H, Yang J, Wang X, Zhao X, Gu W, Bi Y, Peng Y, Xu X, Xia H, Li F, Xu X, Yang H, Xu G, Madsen L, Kristiansen K, Ning G, Wang W. Analyses of gut microbiota and plasma bile acids enable stratification of patients for antidiabetic treatment. Nat Commun 2017;8(1):1785.
[196] Li M, Liu S, Wang M, Hu H, Yin J, Liu C, Huang Y. Gut microbiota dysbiosis associated with bile acid metabolism in neonatal cholestasis disease. Sci Rep 2020;10(1):7686.
[197] Salvioli G, Salati R, Bondi M, Fratalocchi A, Sala BM, Gibertini A. Bile acid transformation by the intestinal flora and cholesterol saturation in bile. Effects of Streptococcus faecium administration. Digestion 1982;23(2):80–88.
[198] Macdonald IA, White BA, Hylemon PB. Separation of 7 alpha- and 7 beta-hydroxysteroid dehydrogenase activities from clostridium absonum ATCC# 27555 and cellular response of this organism to bile acid inducers. J Lipid Res 1983;24(9):1119–1126.
[199] Lepercq P, Gerard P, Beguet F, Raibaud P, Grill JP, Relano P, Cayuela C, Juste C. Epimerization of chenodeoxycholic acid to ursodeoxycholic acid by Clostridium baratii isolated from human feces. FEMS Microbiol Lett 2004;235(1):65–72.
[200] Lepercq P, G P, Béguet F, Grill J-P, Relano P, Cayuela C, Juste C. Isolates from normal human intestinal flora but not lactic acid bacteria exhibit 7a- and 7b-hydroxysteroid dehydrogenase activities. Microb Ecol Health Dis 2004;16(4):195–201.
[201] Ridlon JM, Kang DJ, Hylemon PB. Bile salt biotransformations by human intestinal bacteria. J Lipid Res 2006;47(2):241–259.
[202] Yoon S, Yu J, McDowell A, Kim SH, You HJ, Ko G. Bile salt hydrolase-mediated inhibitory effect of Bacteroides ovatus on growth of Clostridium difficile. J Microbiol 2017;55(11):892–899.
[203] Gopal-Srivastava R, Hylemon PB. Purification and characterization of bile salt hydrolase from Clostridium perfringens. J Lipid Res 1988;29(8):1079–1085.
[204] Chand D, Panigrahi P, Varshney N, Ramasamy S, Suresh CG. Structure and function of a highly active Bile Salt Hydrolase (BSH) from Enterococcus faecalis and post-translational processing of BSH enzymes. Biochim Biophys Acta Proteins Proteom 2018;1866(4):507–518.
[205] Kim GB, Yi SH, Lee BH. Purification and characterization of three different types of bile salt hydrolases from Bifidobacterium strains. J Dairy Sci 2004;87(2):258–266.
[206] Allain T, Chaouch S, Thomas M, Vallee I, Buret AG, Langella P, Grellier P, Polack B, Bermudez-Humaran LG, Florent I. Bile-salt-Hydrolases from the probiotic strain Lactobacillus johnsonii La1 mediate anti-giardial activity in vitro and in vivo. Front Microbiol 2017;8:2707.
[207] Prete R, Long SL, Gallardo AL, Gahan CG, Corsetti A, Joyce SA. Beneficial bile acid metabolism from Lactobacillus plantarum of food origin. Sci Rep 2020;10(1):1165.
[208] Lepercq P, Hermier D, David O, Michelin R, Gibard C, Beguet F, Relano P, Cayuela C, Juste C. Increasing ursodeoxycholic acid in the enterohepatic circulation of pigs through the administration of living bacteria. Br J Nutr 2005;93(4):457–469.
[209] Markwick LJ, Riva A, Ryan JM, Cooksley H, Palma E, Tranah TH, Manakkat Vijay GK, Vergis N, Thursz M, Evans A, Wright G, Tarff S, O'Grady J, Williams R, Shawcross DL, Chokshi S. Blockade of PD1 and TIM3 restores innate and adaptive immunity in patients with acute alcoholic hepatitis. Gastroenterology 2015;148(3):590–602 e10.
[210] Lee KC, Baker LA, Stanzani G, Alibhai H, Chang YM, Jimenez Palacios C, Leckie PJ, Giordano P, Priestnall SL, Antoine DJ, Jenkins RE, Goldring CE, Park BK, Andreola F, Agarwal B, Mookerjee RP, Davies NA, Jalan R. Extracorporeal liver assist device to exchange albumin and remove endotoxin in acute liver failure: results of a pivotal pre-clinical study. J Hepatol 2015;63(3):634–642.
[211] Stadlbauer V, Krisper P, Aigner R, Haditsch B, Jung A, Lackner C, Stauber RE. Effect of extracorporeal liver support by MARS and Prometheus on serum cytokines in acute-on-chronic liver failure. Crit Care 2006;10(6):R169.
[212] **Guevara M**, **Terra C**, Nazar A, Sola E, Fernandez J, Pavesi M, Arroyo V, Gines P. Albumin for bacterial infections other than spontaneous bacterial peritonitis in cirrhosis. A randomized, controlled study. J Hepatol 2012;57(4):759–765.
[213] **Sola E**, **Sole C**, Simon-Talero M, Martin-Llahi M, Castellote J, Garcia-Martinez R, Moreira R, Torrens M, Marquez F, Fabrellas N, de Prada G, Huelin P, Lopez Benaiges E, Ventura M, Manriquez M, Nazar A, Ariza X, Sune P, Graupera I, Pose E, Colmenero J, Pavesi M, Guevara M, Navasa M, Xiol X, Cordoba J, Vargas V, Gines P. Midodrine and albumin for prevention of complications in patients with cirrhosis awaiting liver transplantation. A randomized placebo-controlled trial. J Hepatol 2018;69(6):1250–1259.

[214] China L, Freemantle N, Forrest E, Kallis Y, Ryder SD, Wright G, Portal AJ, Becares Salles N, Gilroy DW, O'Brien A, Investigators AT. A randomized trial of albumin infusions in hospitalized patients with cirrhosis. N Engl J Med 2021;384(9):808–817.
[215] Sehgal R, Maiwall R, Rajan V, Islam M, Baweja S, Kaur N, Kumar G, Ramakrishna G, Sarin SK, Trehanpati N. Granulocyte-macrophage colony-stimulating factor modulates myeloid-derived suppressor cells and treg activity in decompensated cirrhotic patients with sepsis. Front Immunol 2022;13:828949.
[216] Kedarisetty CK, Anand L, Bhardwaj A, Bhadoria AS, Kumar G, Vyas AK, David P, Trehanpati N, Rastogi A, Bihari C, Maiwall R, Garg HK, Vashishtha C, Kumar M, Bhatia V, Sarin SK. Combination of granulocyte colony-stimulating factor and erythropoietin improves outcomes of patients with decompensated cirrhosis. Gastroenterology 2015;148(7):1362–13670 e7.
[217] Prajapati R, Arora A, Sharma P, Bansal N, Singla V, Kumar A. Granulocyte colony-stimulating factor improves survival of patients with decompensated cirrhosis: a randomized-controlled trial. Eur J Gastroenterol Hepatol 2017;29(4):448–455.
[218] Newsome PN, Fox R, King AL, Barton D, Than NN, Moore J, Corbett C, Townsend S, Thomas J, Guo K, Hull D, Beard HA, Thompson J, Atkinson A, Bienek C, McGowan N, Guha N, Campbell J, Hollyman D, Stocken D, Yap C, Forbes SJ. Granulocyte colony-stimulating factor and autologous CD133-positive stem-cell therapy in cirrhosis (REALISTIC): an open-label, randomised, controlled phase 2 trial. Lancet Gastroenterol Hepatol 2018;3(1):25–36.
[219] Spahr L, Lambert JF, Rubbia-Brandt L, Chalandon Y, Frossard JL, Giostra E, Hadengue A. Granulocyte-colony stimulating factor induces proliferation of hepatic progenitors in alcoholic steatohepatitis: a randomized trial. Hepatology 2008;48(1):221–229.
[220] Gaia S, Olivero A, Smedile A, Ruella M, Abate ML, Fadda M, Rolle E, Omede P, Bondesan P, Passera R, Risso A, Aragno M, Marzano A, Ciancio A, Rizzetto M, Tarella C. Multiple courses of G-CSF in patients with decompensated cirrhosis: consistent mobilization of immature cells expressing hepatocyte markers and exploratory clinical evaluation. Hepatol Int 2013;7(4):1075–1083.

Journal of Hepatology, Volume *5*

Supplemental information

Circulating neutrophil anti-pathogen dysfunction in cirrhosis

Irina Balazs and Vanessa Stadlbauer

Circulating neutrophil anti-pathogen dysfunction in cirrhosis

Irina Balazs, Vanessa Stadlbauer

Table of contents

Table S1..2

Supplementary references..15

Table S1. Overview of neutrophil functional defects in patients with cirrhosis including patients 'demographics, liver cirrhosis severity and etiology, neutrophil isolation technique and methodology used to study neutrophil function.

Study	Patient demographics	Severity and etiology of cirrhosis	Bacterial infections	Neutrophil isolation	Method	Result
**Chemotaxis**						
Rajkovic *et al.,* 1984 [1]	48 patients alcohol associated liver disease – 45 patients with cirrhosis, 12 patients with acute alcoholic hepatitis, 3 patients with fatty change of liver (27-66 years old; 27 males, 21 females); 21 healthy controls	alcohol	7 patients with bacterial infections	Ficoll-Triosil gradient centrifugation	"Leading front" technique	Reduced migration towards healthy serum as chemoattractant compared to healthy donor neutrophils. No correlation with bacterial infections.
Fiuza *et al.,* 2000 [2]	22 cirrhotic patients with ascites (15 male, 7 female; age 55.8+/-10.4); 6 healthy volunteers	Child-Pugh B-10 patients, Child-Pugh C – 12 patients; etiology: viral (50%), alcohol (22%), both alcohol and viral (18%), unknown (9%)	12 patients with 1 or more episodes of bacterial infection in the 6 months before inclusion in the study	Not applicable	Skin window technique	Patients with cirrhosis have a decreased neutrophil migration into skin windows filled with gram-negative or gram-positive bacteria and it correlates with the liver dysfunction severity and presence of previous infections.
Onishi *et al.,* 1989 [3]	17 patients with alcohol associated liver disease including 9 patients with liver cirrhosis (1 female, 8 male; age 38-62 years old); 16 patients with non-alcoholic liver cirrhosis (8 female, 8 male; age 31-75 years old); 18 healthy volunteers	Alcohol and non-alcoholic	-	Dextran sedimentation	Modification of a Boyden technique using chemotactic chambers	Serum chemotactic inhibitory activity higher in alcoholic liver disease compared to healthy controls and higher in alcohol associated cirrhosis compared to non-alcoholic cirrhosis
Van Epps *et al.,* 1975 [4]	42 patients with alcohol associated liver disease (37 male, 5 female, age 23-68 years old) including 38 patients with cirrhosis, 20 patients with alcoholic hepatitis, 4 patients with fatty change.	Alcohol	9 patients with active infection	Plasma gel sedimentation of erythrocytes	Boyden technic	Chemotactic inhibitory activity is in 21 of 42 patients and does not correlate with the type of liver disease and degree of liver function, but is associated with infections
Campbell *et al.,* 1981 [5]	44 patients with alcohol associated liver disease (22 male, 22 female; age 17-81), including 31 patients with cirrhosis	Alcohol (13 patients), chronic active hepatitis (10 patients), primary biliary cirrhosis (8 patients), cryptogenic cirrhosis (13 patients)	-	Dextran sedimentation	"Leading front" technique	Neutrophil migration reduced in patients with alcohol related liver disease in presence of autologous plasma, but not healthy donor plasma; depressed migration, but to a lesser extent, in patients with cryptogenic cirrhosis, but not in patients with chronic active hepatitis or

						primary biliary cirrhosis
Fiuza *et al.*, 2002 [6]	14 cirrhotic patients (56.42 ± 11.62 years; 9 male, 5 female); 14 healthy controls; no patient consuming alcohol at the time of the study	Child-Pugh classes B (6 patients) and C (8 patients); hepatitis C (6 patients), alcohol (3 patients), hepatitis B (3 patients), Budd-Chiari syndrome (1 patient), hepatitis C+alcohol (1 patient)	No infections within the preceding week	Dextran sedimentation and gradient centrifugation	Transwell inserts with 3um pores seeded with HMEC-1 cells monolayers; neutrophils migrated through the pores; neutrophils counted with hemacytometer .	Decreased transendothelial migration towards fMLF of neutrophils from cirrhotic patients
Artru *et al.*, 2020 [7]	24 patients with cirrhosis (19 male, 5 female; age 52.7 – 66.9)	Child-Pugh score 7-11; alcohol	-	MACSxpress Neutrophil isolation human kit	Transwell inserts with 3um pores, migrated neutrophils counted with flow cytometry	Decreased neutrophil migration towards IL-8 in cirrhotic patients
Claria *et al.*, 1998 [8]	12 patients with cirrhosis and ascites; 13 healthy donors	-	Patients without evidence of bacterial infections	Ficoll-Hypaque gradient centrifugation, Boyum method	Transwell inserts with 3um pores, migrated neutrophils are counted by lysing cells with 0.025 mol/L NaOH with 0.1% SDS and then measuring the fluorescence	Decreased migration of neutrophils isolated from patients with cirrhosis in response to leukotriene B4
Langer *et al.*, 2023 [9]	11 patients with compensated cirrhosis (mean age 53.18; 6 male, 5 female), 84 patients with acute decompensation (AD) of cirrhosis without acute-on-chronic liver failure (ACLF) (mean age 54.87, 46 male, 38 female), 30 patients with ACLF (mean age 56.7; 20 male, 10 female); 24 healthy controls	Compensated (Child-Pugh score mean 5.27), AD (Child-Pugh score mean 8.54), ACLF (Child-Pugh score mean 9.37); etiologies: viral, non-alcoholic steatohepatitis (NASH), alcohol, cholestatic, others	AD – 26 patients with infections, ACLF – 19 patients with infections	MACSxpress® Whole Blood Neutrophil Isolation Kit	96-well flat-bottom plates in X-Vivo 10 Medium (Lonza) is used; neutrophil migration is counted by imaging with a Leica DMI6000 B (Leica Microsystems)	Decreased migration towards fMLF (proportion of migrating neutrophils) in patients with AD of cirrhosis and ACLF, correlated with severity of cirrhosis
Laffi *et al.*, 1993 [10]	10 cirrhotic patients (5 female, 5 male; age 42-65); 10 healthy controls	Child-Pugh B (4 patients), Child-Pugh C (6 patients); hepatitis C (8 patients), hepatitis B (1 patient), hepatitis B and C (1 patient)	No infections	Ficoll-Hypaque gradient	Modification of a Boyden technique using chemotactic chambers	No neutrophil chemotaxis defect in response to casein in cirrhosis; no differences in random migration of neutrophils from cirrhotic patients

Knooihuizen *et al.,* 2021[11]	21 patients with cirrhosis, 23 healthy controls	Alcohol (57%), non-alcoholic fatty liver disease (29%), hepatitis C virus (14%), other (19%); 6 patients with AD, 15 patients had ACLF	4 patients with spontaneous bacterial peritonitis	EasySep Direct Human Neutrophil Isolation Kit	Time-lapse imaging of swarming performed with Nikon Ti-E microscope	Swarming of cirrhotic patients' neutrophils in order to control Candida albicans hyphae growth is impaired compared to healthy controls
**Phagocytosis**						
De Fernandez *et al.,* 1987 [12]	57 patients with liver disease; no significant alcohol consumption within 10 days before study	19 males and 3 females with alcohol associated liver disease (mean age 54) including 14 patients with cirrhosis; 2 males and 19 females with primary biliary cirrhosis (mean age 60.4); 5 male and 9 female patients with chronic active hepatitis (mean age 65) of mainly autoimmune etiology;	All patients clinically free from infections at the time of study	Dextran sedimentation	Neutrophils are incubated with *S.aureus* and phagocytosis is calculated as original number of *S.aureus* minus viable extracellular *S.aureus* after incubation divided by the original number of *S.aureus* and multiplied by 100	Defective phagocytosis in alcohol associated liver disease and primary biliary cirrhosis, but not chronic active hepatitis
Rajkovic *et al.,* 1986 [13]	40 patients with alcohol associated liver disease (39-66 years), all had cirrhosis, including 18 patients with superimposed acute alcoholic hepatitis; 20 healthy controls	alcohol	No active bacterial infection at inclusion	Dextran sedimentation and Ficoll-Hypaque gradient centrifugation	Neutrophils are incubated with bacteria in 96 well plate with added [^{3}H]uridine; phagocytosis index is calculated based on the radioactivity measurements	Defective phagocytosis of *S.aureus* and *E.coli* in neutrophils from patients with alcohol associated cirrhosis, no association with disease severity
Fiuza *et al.,* 2000 [2]	22 cirrhotic patients with ascites (15 male, 7 female; age 55.8+/-10.4) and 6 healthy volunteers	Child-Pugh B - 10 patients, Child-Pugh C – 12 patients; etiology: viral (50%), alcohol (22%), both alcohol and viral (18%), unknown (9%)	12 patients with 1 or more episodes of bacterial infection in the 6 months before inclusion in the study	Not applicable	Skin window technique, phagocytosis assessed with flow cytometry	Phagocytosis of *E.coli* in neutrophils from cirrhotic patients is impaired compared to healthy controls; the decrease in phagocytosis of *E.coli* is more pronounced in patients with previous bacterial infections and correlates with the severity of liver dysfunction
Mookerjee *et al.,* 2007 [14]	63 patients with AD of alcohol associated cirrhosis; 20 healthy controls	Alcohol cirrhosis and superimposed inflammatory alcoholic hepatitis	No clinical or microbiological evidence of infection	Not applicable	The Phagotest kit (Orpegen Pharma) with whole blood	Neutrophil phagocytic capacity of *E. coli* is impaired in cirrhosis (cirrhosis +alcoholic hepatitis)
Stadlbauer *et al.,* 2008 [15]	20 patients with alcohol associated cirrhosis (18-75 years; 15 male, 5 female); 13 healthy controls	Alcohol	No clinical or microbiological evidence of infection	Not applicable	The Phagotest kit (Orpegen Pharma) with whole blood	Reduced neutrophil phagocytic capacity of *E.coli* in cirrhotic patients
Huang *et al.,* 2016 [16]	48 healthy volunteers and 100 cirrhotic patients (77 male, 23	26 patients with Child-Pugh A, 26 patients with Child-Pugh B, 48 patients in Child-Pugh C; Etiologies:	-	Not applicable	The Phagotest kit (Orpegen Pharma) with whole blood	Reduced neutrophil phagocytic capacity of *E.coli* in cirrhotic patients of Child-Pugh C

	female; mean age: 55 ± 13 years)	alcohol, hepatitis B and hepatitis C				
Sehgal *et al.,* 2022 [17]	70 patients with decompensated cirrhosis (63 male, 7 female; age 22-60); 15 healthy controls	Decompensated cirrhosis; alcohol predominant etiology (70%)	40 patients with sepsis	Not applicable	The Phagotest kit (Celonic) with whole blood	Reduced neutrophil phagocytic capacity of *E.coli* in cirrhotic patients
Horvath *et al.,* 2016 [18]	80 patients with cirrhosis (mean age probiotics group 60, placebo group 56; 58 male, 22 female); 51 healthy controls; no alcohol abuse within 2 weeks before the study	Alcohol (44), hepatitis C (13), other (22); Child-Pugh A (61), Child-Pugh B+C (19)	No active infection at screening	Not applicable	The Phagotest kit (Glycotope) with whole blood	Percentage of phagocytic neutrophils is decreased in blood of cirrhotic patients
Taylor *et al.,* 2014 [19]	62 patients with cirrhosis (age 39-61; 17 female)	Stable cirrhosis (49), ACLF (13); etiologies: alcohol, hepatitis C, autoimmune liver disease, other	7 patients with active infection in ACLF group	Not applicable	The Phagotest kit (Orpegen Pharma) with whole blood	Percentage of phagocytic neutrophils is decreased in blood of cirrhotic patients; degree of this dysfunction is increasing with increasing severity of cirrhosis, but is not different between the etiologies of cirrhosis
Wu *et al.,* 2021 [20]	18 patients with hepatitis B virus related ACLF, 14 patients with compensated liver cirrhosis, 13 healthy controls	Hepatitis B virus ACLF or compensated liver cirrhosis	No active bacterial infection	Not applicable	The Phagotest kit (Glycotope) with whole blood	Decreased percentage of phagocytosing neutrophils (*E.coli*) in both compensated cirrhosis and ACLF, with the more pronounce defect in ACLF. Phagocytic activity is significantly decreased in ACLF compared to healthy controls.
Balazs *et al.,* 2022 [21]	109 cirrhotic patients (mean age alcohol 56, hepatitis C 60, other 55; alcohol 42 male, 12 female, hepatitis C 22 male, 10 female, other 13 male, 10 female); 21 healthy controls	Alcohol (54 patients), hepatitis C (32 patients), other (23 patients); Child-Pugh A (79 patients), Child-Pugh B+C (30 patients)	No active bacterial infection	Not applicable	The Phagotest kit (Celonic) with whole blood	Percentage of phagocytic neutrophils is decreased in blood of cirrhotic patients
Leber, Balazs *et al.,* 2021 [22]	85 chronic hepatitis C patients, including 56 patients with cirrhosis (mean age 58; 34 female, 51 male); 21 healthy controls	Hepatitis C; Child-Pugh A (42 patients), Child-Pugh B (12 patients), Child-Pugh C (2 patients)	No active bacterial infection	Not applicable	The Phagotest kit (Glycotope) with whole blood	Phagocytic capacity of *E.coli* is reduced and a percentage of non-phagocytic neutrophils is increased in cirrhotic patients

Laffi *et al.*, 1993 [10]	10 cirrhotic patients (5 female, 5 male; age 42-65); 10 healthy controls	Child-Pugh B (4 patients), Child-Pugh C (6 patients); hepatitis C (8 patients), hepatitis B (1 patient), hepatitis B and C (1 patient)	No infections	Ficoll-Hypaque gradient centrifugation, Böyum method	Light microscopy	No phagocytosis defect of *C. albicans* is shown in neutrophils of cirrhotic patients
Makkar *et al.*, 2020 [23]	40 patients with AD of cirrhosis (age 46.3; 35 male); 10 healthy controls	10 patients of each grade 0, 1, 2 and ACLF; alcohol (30 patients), hepatitis B+alcohol (2 patients), hepaitits C+alcohol (2 patients), autoimmune (4 patients), NASH (2 patients), others (1 patient)	No active infection	Not applicable	Cayman's Phagocytosis Assay Kit with whole blood	In ACLF impaired phagocytosis of latex beads has been reported and associated with 90-day survival
Tritto *et al.*, 2011 [24]	108 patients with liver cirrhosis (79 male, 29 female; mean age 57.5). For whole blood experiments: 8 patients (mean age 48; 6 male, 2 female) and 5 healthy controls	Stable cirrhosis (Child-Pugh A or B 58 patients, Child-Pugh C 50 patients); alcohol 41 patients, HBV/HCV 45 patients, other 22 patients. For whole blood experiments: Child-Pugh A 5 patients, Child-Pugh B – 3 patients; alcohol 8 patients	No bacterial infections at inclusion	Polymorphprep	The Phagotest kit (Orpegen Pharma) - healthy donor neutrophils incubated with patients' or healthy donor plasma or in whole blood	Decreased phagocytic capacity of normal neutrophils incubated with patient's plasma compared to control plasma. It did not correlate with cirrhosis etiology or 3-month mortality and morbidity, but correlated with disease severity. Phagocytic capacity of peripheral blood neutrophils from patients with compensated liver cirrhosis was not different compared to healthy controls.
Campbell *et al.*, 1981 [5]	44 patients with alcohol associated liver disease (22 male, 22 female; age 17-81), including 31 patients with cirrhosis	Alcohol (13 patients), chronic active hepatitis (10 patients), primary biliary cirrhosis (8 patients), cryptogenic cirrhosis (13 patients)	-	Dextran sedimentation	Neutrophils are incubated with *C.albicans* with added [^{3}H]uridine (which is incorporated only by viable extracellular organisms); phagocytosis index is calculated based on the radioactivity measurements	Cirrhotic neutrophil phagocytosis of *C.albicans* opsonized with autologous plasma was not different compared to phagocytosis of *C.albicans* opsonized with healthy donor plasma, except for patients with chronic active hepatitis
**Killing capacity**						
Rajkovic *et al.*, 1986 [13]	40 patients with alcohol associated liver disease (39-66	Alcohol	No active bacterial	Dextran sedimentation and Ficoll-	Neutrophils incubated with bacteria in 96	Intracellular killing of *S. aureus* and *E.*

	years), all had cirrhosis, including 18 patients with superimposed acute alcoholic hepatitis; 20 healthy controls		infection at inclusion	Hypaque gradient centrifugation	well plate with added [^{3}H]uridine; killing index is calculated based on the radioactivity measurements	*coli* is impaired in alcohol related cirrhosis
Boussif *et al.,* 2016 [25]	32 patients with alcohol associated cirrhosis (mean age 57.4; 6 female); Alcohol consumption stopped for at least 3 days	Alcohol; Child-Pugh B or C;	No bacterial infection within one week; 18 patients with previous bacterial infections	Dextran sedimentation and Ficoll-Hypaque gradient centrifugation	The number of viable bacteria after incubation with neutrophils is calculated	Impaired *E.coli* killing upon stimulation with fMLF in neutrophils from cirrhotic patients
De Fernandez *et al.,* 1987 [12]	57 patients with liver disease	19 males and 3 females with alcohol associated liver disease (mean age 54) including 14 patients with cirrhosis; 2 males and 19 females with PBC (mean age 60.4); 5 male and 9 female patients with chronic active hepatitis (mean age 65) of mainly autoimmune etiology; no significant alcohol consumption within 10 days before study	All patients clinically free from infections at the time of study	Dextran sedimentation	Intracellular killing capacity of *S.aureus* calculated as viable intracellular *S.aureus* count divided by the difference between total viable *S.aureus* after incubation with neutrophils and viable extracellular *S.aureus* after incubation with neutrophils	No differences in neutrophil intracellular killing capacity of *S. aureus* in alcohol associated cirrhosis and PBC, despite decreased total bacterial killing, which the authors explain by decreased percentage of phagocytic neutrophils
Knooihuizen *et al.,* 2021[11]	21 patients with cirrhosis, 23 healthy controls	Alcohol (57%), non-alcoholic fatty liver disease (29%), hepatitis C virus (14%), other (19%); 6 patients with AD, 15 patients had ACLF	4 patients had spontaneous bacterial peritonitis	EasySep Direct Human Neutrophil Isolation Kit	Neutrophils are incubated with *Candida albicans* in 96 well plates; percentage of remaining live pathogen is measured with PrestoBlue Cell Viability Reagent	Impaired killing capacity of *Candida albicans* by neutrophils from cirrhotic patients
Laffi *et al.,* 1993 [10]	10 cirrhotic patients (5 female, 5 male; age 42-65); 10 healthy controls	Child-Pugh B (4 patients), Child-Pugh C (6 patients); hepatitis C (8 patients), hepatitis B (1 patient), hepatitis B and C (1 patient)	No infections	Ficoll-Hypaque gradient	Killing index is determined as the ratio of killed *C.albicans* to total input number; dead *C.albicans* is identified by staining with methylene blue after incubation with neutrophils	No *C.albicans* killing capacity defect in neutrophils from cirrhotic patients
Campbell *et al.,* 1981 [5]	44 patients with alcohol associated liver disease (22 male, 22 female; age 17-81), including 31	Alcohol (13 patients), chronic active hepatitis (10 patients), primary biliary cirrhosis (8		Dextran sedimentation	Neutrophils are incubated with *C.albicans* with added	Cirrhotic neutrophil killing of *C.albicans* opsonized with autologous plasma was not different

	patients with cirrhosis	patients), cryptogenic cirrhosis (13 patients)			[^{3}H]uridine (which is incorporated only by viable extracellular organisms); killing activity is calculated based on the radioactivity measurements	compared to killing of *C.albicans* opsonized with healthy donor plasma
**ROS production**						
Horvath *et al.,* 2016 [18]	80 patients with cirrhosis (mean age probiotics group 60, placebo group 56; 58 male, 22 female); 51 healthy controls; no alcohol abuse within 2 weeks before the study	Alcohol (44 patients), hepatitis C (13 patients), other (22 patients); Child-Pugh A (61 patients), Child-Pugh B+C (19 patients)	No active infection at screening	Not applicable	The Phagoburst kit (Glycotope) with whole blood	Elevated percentage of neutrophils with basal ROS production and in response to fMLF, but unchanged in response to *E.coli* in cirrhosis
Mookerjee *et al.,* 2007 [14]	63 patients with AD of alcohol associated cirrhosis; 20 healthy controls	Alcohol; cirrhosis and superimposed inflammatory alcoholic hepatitis	No clinical or microbiological evidence of infection	Not applicable	The Phagoburst kit (Orpegen Pharma) with whole blood	Elevated percentage of neutrophils with basal ROS production and in response to fMLF, but unchanged in response to *E.coli* in cirrhosis
Leber, Balazs *et al.,* 2021 [22]	85 chronic hepatitis C patients, including 56 patients with cirrhosis (mean age 58; 34 female, 51 male); 21 healthy controls	Hepatitis C; Child-Pugh A (42 patients), Child-Pugh B (12 patients), Child-Pugh C (2 patients)	No active bacterial infection	Not applicable	The Phagoburst kit (Glycotope) with whole blood	Elevated percentage of neutrophils with basal ROS production in cirrhosis, but unchanged ROS production in response to fMLF and *E.coli*
Bruns *et al.,* 2011 [26]	84 patients with liver cirrhosis (male 77%, age 37-82); no active alcohol consumers; 29 healthy controls	Alcohol associated cirrhosis (80%), 4 patients with viral cirrhosis, 6 patients with cryptogenic cirrhosis, 7 patients with other etiologies of cirrhosis; Child-Pugh A (7 patiens), Child-Pugh B (43 patients), Child-Pugh C (34 patients)	45 patients had signs of infection at the inclusion	Not applicable	The Phagoburst kit (Glycotope) with whole blood	Elevated percentage of neutrophils with basal ROS production, as well as basal intracellular ROS produced in cirrhosis; no difference between infected and not infected patients, but association with disease severity. No difference in percentage of neutrophils producing ROS in response to fMLF between cirrhosis and healthy, however, intracellular ROS upon fMLF stimulation is higher in cirrhosis – no correlation with infections. Percentage of neutrophils produced ROS in response to *E.coli* in

						cirrhosis is unchanged compared to healthy, the amount of intracellular ROS in response to *E.coli* is increased in cirrhosis without infection.
Makkar *et al.,* 2020 [23]	40 patients with AD of cirrhosis (age 46.3; 35 male); 10 healthy controls	10 patients of each grade 0, 1, 2 and ACLF; alcohol (30 patients), hepatitis B+alcohol (2), hepatitis C+alcohol (2 patients), autoimmune (4 patients), NASH (2), others (1 patient)	No active infection	Not applicable	Oxidation of dihydrorhodamine−123 to rhodamine measured by flow cytometry	Elevated percentage of basal ROS production in neutrophils from patients with AD and ACLF
Balazs *et al.,* 2022 [21]	109 cirrhotic patients (mean age alcohol 56, hepatitis C 60, other 55; alcohol 42 male, 12 female, hepatitis C 22 male, 10 female, other 13 male, 10 female); 21 healthy controls	Alcohol (54 patients), hepatitis C (32 patients), other (23 patients); Child-Pugh A (79 patients), Child-Pugh B+C (30 patients)	No active bacterial infection	Not applicable	The Phagoburst kit (Celonic) with whole blood	Elevated percentage of neutrophils with basal ROS production and ROS production in response to fMLF, but decreased ROS production in response to *E.coli* in cirrhosis. Intracellular ROS production is unchanged
Huang *et al.,* 2016 [16]	48 healthy volunteers and 100 cirrhotic patients (77 male, 23 female; mean age: 55 ± 13 years)	26 patients with Child-Pugh A, 26 patients with Child-Pugh B, 48 patients in Child-Pugh C; etiologies: alcohol, hepatitis B and hepatitis C	-	Not applicable	The Phagoburst kit (Orpegen Pharma) with whole blood	Significantly higher level of intracellular basal ROS production in neutrophils from cirrhotic patients of Child-Pugh C
Rolas *et al.,* 2013 [27]	17 patients with cirrhosis (age mean 56; 8 female, 9 male);	Child-Pugh B (4 patients) or C (13) patients; alcohol associated	No current bacterial infections	Ficoll-Hypaque gradient	Cytochrome C reduction assay with isolated neutrophils and chemiluminescence in whole blood in presence of luminol	Reduced intracellular basal ROS production and ROS production in response to fMLF; decreased extracellular superoxide release in response to fMLF
Rajkovic *et al.,* 1986 [13]	40 patients with alcohol associated liver disease (39-66 years), all had cirrhosis, including 18 patients with superimposed acute alcoholic hepatitis; 20 healthy controls	Alcohol	No active bacterial infection at inclusion	Dextran sedimentation and Ficoll-Hypaque gradient centrifugation	Cytochrome C reduction assay to measure superoxide, with addition of phenol red to measure peroxide	Extracellular basal superoxide production is unchanged, but reduced in response to zymosan in cirrhotic patients; extracellular hydrogen peroxide levels produced by neutrophils in response to zymosan are not different or higher in cirrhotic

						patients compared to healthy controls
Laffi *et al.*, 1993 [10]	10 cirrhotic patients (5 female, 5 male; age 42-65); 10 healthy controls	Child-Pugh B (4 patients), Child-Pugh C (6 patients); hepatitis C (8 patients), hepatitis B (1 patient), hepatitis B and C (1 patient)	No infections	Ficoll-Hypaque gradient centrifugation, Böyum method	Cytochrome C reduction assay	Extracellular basal superoxide production elevated in cirrhosis, extracellular superoxide production in response to zymosan reduced in cirrhosis
Stadlbauer *et al.,* 2008 [15]	20 patients with alcohol associated cirrhosis (18-75 years; 15 male, 5 female) including 8 cirrhotic patients as disease control; 13 healthy controls	Alcohol	No clinical or microbiological evidence of infection	Not applicable	The Phagoburst kit (Orpegen Pharma) with whole blood	Increased percentage of neutrophils producing ROS in response to fMLF; slightly increased basal ROS production in disease controls
Garfia *et al.,* 2004 [28]	98 patients with liver cirrhosis (66 male, 32 female; age 39-76); 46 healthy controls	Alcohol (45 patients), hepatitis C (17 patients), hepatitis B (7 patients), mixed alcohol and viral (24 patients), autoimmune (1 patient), cryptogenic (4 patients); Child-Pugh A (20 patients), Child-Pugh B (20 patients), Child-Pugh C (58 patients)	24 patients had previous episodes of spontaneous bacterial peritonitis; patients with recent history of bacterial infections excluded	Lymphoprep centrifugation	Cytochrome C reduction assay	Extracellular superoxide release in response to fMLF, TNF-alpha and zymosan decreased in patients with cirrhosis; no association with etiology of cirrhosis, but with severity of liver disease
Rolas *et al.,* 2018 [29]	44 patients with cirrhosis (mean age 59.1 years, 6 female); Alcohol consumption stopped for at least 1 week before the study	Child-Pugh B (14 patients) or C (30 patients); alcohol	16 patients with previous bacterial infections, but no current bacterial infections	Ficoll-Hypaque gradient	Cytochrome C reduction assay	Extracellular superoxide production decreased in response to fMLF in patients with cirrhosis
Weiss *et al.,* 2021 [30]	31 patients with cirrhosis (age 52-64; 23 male)	Advanced cirrhosis (7 patients), acute decompensati on of cirrhosis (7 patients), acute-on-chronic liver failure (17 patients); alcohol (21 patients), non-alcohol steatohepatitis (6 patients), chronic hepatits C (3 patients), chronic hepatitis B (1 patient),	No bacterial infections within 1 week before inclusion	Ficoll-Hypaque gradient	Cytochrome C reduction assay	Decrease in superoxide release in response to fMLF significantly more pronounced in patients with ACLF compared to patients with advanced cirrhosis
Taylor *et al.,* 2014 [19]	62 patients with cirrhosis (age 39-61; 17 female)	Stable cirrhosis (49 patients), ACLF (13 patients); etiologies: alcohol, hepatitis C, autoimmune liver disease, other	7 patients with active infection in ACLF group	Not applicable	The Bursttest kit (Orpegen Pharma) with whole blood	Basal ROS production elevated in ACLF compared to healthy controls, but not in stable cirrhosis and not associated with the severity of disease; ROS production in

						response to *E.coli* unchanged in all cirrhotic patients compared to healthy controls and not associated with either severity or etiology of disease; no difference in ROS production in active and abstinent drinkers
Masini *et al.,* 1995 [31]	12 cirrhotic patients with ascites; 10 healthy controls	-	-	Lymphoprep gradient	Superoxide production assessed with cytochrome c reduction assay; nitric oxide production evaluated using neutrophil capacity to inhibit thrombin-induced platelet aggregation and increase cGMP content in thrombin induced platelets co-incubated with neutrophils	Reduced extracellular superoxide production in response to zymosan in patients with cirrhosis; increased nitric oxide production in response to opsonized zymosan in neutrophils from cirrhotic patients
Wu *et al.,* 2021 [20]	18 patients with hepatitis B virus related ACLF, 14 patients with compensated liver cirrhosis, 13 healthy controls	Hepatitis B virus ACLF or compensated liver cirrhosis	No active bacterial infection	Not applicable	The Phagoburst test (Glycotope)	Elevated number of neutrophils with basal ROS production in both compensated cirrhosis and ACLF, decreased intracellular basal ROS production in neutrophils from patients with compensated cirrhosis
Tritto *et al.,* 2011 [24]	108 patients with liver cirrhosis (79 male, 29 female; mean age 57.5). For whole blood experiments: 8 patients (mean age	Stable cirrhosis (Child-Pugh A or B 58 patients, Child-Pugh C 50 patients); alcohol 41 patients, HBV/HCV 45 patients, other 22 patients.	No bacterial infection at inclusion	Polymorphprep	The Phagoburst kit (Orpegen Pharma) - healthy donor neutrophils incubated with patients' or	Basal ROS production in healthy donor neutrophils incubated with patients' plasma was not

	48; 6 male, 2 female) and 5 healthy controls	For whole blood experiments: Child-Pugh A 5 patients, Child-Pugh B – 3 patients; alcohol 8 patients			healthy donor plasma or in whole blood	significantly different from neutrophils incubated with healthy controls' plasma. No difference in basal ROS production and ROS production in response to E.coli in neutrophils from cirrhotic patients compared to healthy controls.
**Degranulation**						
Rajkovic *et al.,* 1986 [13]	40 patients with alcohol associated liver disease (39-66 years), all had cirrhosis, including 18 patients with superimposed acute alcoholic hepatitis; 20 healthy controls	Alcohol	No active bacterial infection at inclusion	Dextran sedimentation and Ficoll-Hypaque gradient centrifugation	Incubation of neutrophils with Cytohalasin B and zymosan and then measurements of enzymes in supernatants	Enzyme intracellular contents (lysozyme, myeloperoxidase (MPO)) and their release from neutrophil granules upon stimulation with zymosan are reduced in neutrophils from cirrhotic patients; however, authors claim that the release reduction is not dependent on the reduction of the enzymes level inside the granules
Boussif *et al.,* 2016 [25]	32 patients with alcohol associated cirrhosis (mean age 57.4; 6 female); Alcohol consumption stopped for at least 3 days	Alcohol; Child-Pugh B or C;	No bacterial infection within one week; 18 patients with previous bacterial infections	Dextran sedimentation and Ficoll-Hypaque gradient centrifugation	Method is based on the horseradish peroxidase-catalysed oxidation of o-dianisidine by H2O2	Intracellular content of MPO is not altered in neutrophils from cirrhotic patients, but its extracellular release in response to fMLF is decreased; MPO activity unchanged
Garfia *et al.,* 2004 [28]	98 patients with liver cirrhosis (66 male, 32 female; age 39-76); 46 healthy controls	Alcohol (45 patients), hepatitis C (17 patients), hepatitis B (7 patients), mixed alcohol and viral (24 patients), autoimmune (1 patient), cryptogenic (4 patients); Child-Pugh A (20 patients), Child-Pugh B (20 patients), Child-Pugh C (58 patients)	24 patients with previous episodes of spontaneous bacterial peritonitis; patients with recent history of bacterial infections excluded from the study	Lymphoprep centrifugation	Absorbance measured in cell supernatant mixed with tetrametilbenzidine, hydrogen peroxide and sodium phosphate buffer	Decreased MPO activity in cirrhosis

Tranah *et al.*, 2017 [32]	29 patients with alcohol associated cirrhosis (age 45-59, 19 male, 10 female); 15 actively drinking patients, 14 patients abstinent; 12 healthy controls	Alcohol; Child-Pugh score 7-12	No evidence of infection at inclusion	Not applicable	Whole blood incubated with either *E.coli* or fMLF, then stained with fluorescent antibodies (CD66b, CD63, CD11b, MPO, CD16), analysed by flow cytometry. For intracellular granular phenotype a permeabilizatio n step is added	In patients with alcohol associated cirrhosis increased mobilization to the cell surface of the primary neutrophil granules is observed. Active alcohol consumption decreases the hyper responsiveness of cirrhotic neutrophils.
Wu *et al.*, 2021 [20]	18 patients with hepatitis B virus related ACLF, 14 patients with compensated liver cirrhosis, 13 healthy controls	Hepatitis B virus ACLF or compensated liver cirrhosis	No active bacterial infection	Not applicable	Whole blood stained with anti-MPO-FITC and analysed with flow cytometry	Decreased number of neutrophils producing MPO and intracellular MPO in neutrophils from patients with compensated cirrhosis, but not with ACLF
**NETs formation**						
Agraz-Cibrian *et al.*, 2016 [33]	60 patients with cirrhosis; 20 healthy controls	Alcohol (42 patients), hepatitis C (6 patients), other (12 patients); compensated cirrhosis (20 patients), decompensate d cirrhosis with ascites (20 patients), decompensate d cirrhosis with spontaneous bacterial peritonitis (20 patients)	20 patients with spontaneous bacterial peritonitis	Ficoll Histopaque 1119/1077 density gradient	Immunofluoresc ence; fluorimetry (DNA/NETs)	Decrease in NETs formation in response to phorbol-12-myristat-13-acetat (PMA) in patients with liver cirrhosis complicated with spontaneous bacterial peritonitis compared to healthy controls
Agraz-Cibrian *et al.*, 2018 [34]	40 patients with liver cirrhosis (27 male, 13 female; age group with ascites 49.56, group with ascites and spontaneous bacterial peritonitis 55.6); 20 healthy controls	Alcohol (34 patients), hepatitis C (4 patients), other (2 patients); decompensate d cirrhosis with ascites (20 patients), decompensate d cirrhosis with ascites and spontaneous bacterial peritonitis (20 patients)	20 patients with spontaneous bacterial peritonitis	Ficoll Histopaque 1119/1077 density gradient	Immunofluoresc ence	Decrease in NETs formation in response to PMA in patients with liver cirrhosis complicated with spontaneous bacterial peritonitis compared to healthy controls
Sehgal *et al.*, 2022 [17]	70 patients with decompensated cirrhosis (63 male, 7 female; age 22-60); 15 healthy controls	Decompensate d cirrhosis; alcohol predominant etiology (70%)	40 patients with sepsis	Polymorphoprep	Immunofluoresc ence; fluorimetry	Plasma of patients with decompensated cirrhosis induces NETs formation in isolated neutrophils from healthy controls
Zenlander *et al.*, 2021 [35]	95 patients with cirrhosis (mean age 63.4; 62 male), 82	Alcohol (34 patients), NASH (19	-	Not applicable	ELISA (H3Cit-DNA, MPO-	Both H3Cit-DNA and MPO-DNA levels

	patients with hepatocellular carcinoma; 50 healthy controls	patients), viral hepatitis (20 patients), other (22 patients); severity in cirrhosis group: Child-Pugh A (47 patients), Child-Pugh B (34 patients), Child-Pugh C (14 patients)			DNA) with plasma	significantly elevated in patients with cirrhosis compared to healthy controls, indicating increased NETs formation in this group; associated with disease severity
Blasi *et al.*, 2019 [36]	52 patients with AD of cirrhosis (mean age 58, 29 male), 57 patients with ACLF (mean age 59, 40 male); 40 healthy controls	Alcohol (AD: 32, ACLF 33), viral (ACLF 11), NASH (AD 10, ACLF 6), biliary (AD 3, ACLF 2), other (AD 7, ACLF 5); Child-Pugh (AD 7-10, ACLF 8-12)	7 patients with AD and 26 patients with ACLF had infections	Not applicable	Quant-iT PicoGreen double strand DNA assay kit (concentration of cell-free DNA in plasma); ELISA (MPO-DNA in plasma)	Cell-free DNA and MPO-DNA complexes in plasma are elevated in both AD and ACLF compared to healthy controls
Wu *et al.*, 2021 [20]	18 patients with hepatitis B virus related ACLF, 14 patients with compensated liver cirrhosis, 13 healthy controls	Hepatitis B virus ACLF or compensated liver cirrhosis	No active bacterial infection	Not applicable	Whole blood stained with FITC-MPO antibodies and SYTOX red and analysed by flow cytometry or imaging flow cytometry	NETs formation in response to *E.coli*, fMLF and PMA as well as spontaneous NETs formation is elevated in patients with compensated cirrhosis and ACLF
**Apoptosis and viability**						
Kusaba *et al.*, 1998 [37]	10 cirrhotic patients (3 men and 7 women; 42-75 years old); 10 healthy controls	Hepatitis C (8 patients), hepatitis B (2 patients);	-	Percoll gradient	Viability is assessed with trypan blue; apoptosis is assessed with light microscopy and TUNEL assay	Increased apoptosis rate and decreased viability of neutrophils isolated from neutropenic patients with viral liver cirrhosis 24 hours after isolation from whole blood
Ramirez *et al.,* 2004 [38]	17 patients with cirrhosis and ascites (mean age 61.8; 10 male, 7 female); 13 patients with compensated chronic liver disease	Hepatitis C (10 patients), hepatitis B (1 patient), alcohol (5 patients), other (1 patient)	-	Ficoll-Hypaque gradient centrifugation, Boyum method	Viability is determined with MTT assay and trypan blue; apoptosis is assessed with light microscopy and TACs apoptotic DNA laddering kit	Decreased viability from cirrhotic patients; increased apoptosis of neutrophils in patients with decompensated cirrhosis

Supplementary references

1. Rajkovic, I. A.; Yousif-Kadaru, A. G.; Wyke, R. J.; Williams, R., Polymorphonuclear leucocyte locomotion and aggregation in patients with alcoholic liver disease. *Clin Exp Immunol* **1984,** 58, (3), 654-62.
2. Fiuza, C.; Salcedo, M.; Clemente, G.; Tellado, J. M., In vivo neutrophil dysfunction in cirrhotic patients with advanced liver disease. *J Infect Dis* **2000,** 182, (2), 526-33.
3. Onishi, S.; Saibara, T.; Maeda, T.; Yamamoto, Y.; Ito, K., Serum inhibition of complement derived leukocyte chemotaxis and levels of immunoglobulin A subclass in alcoholic liver disease. *Gastroenterol Jpn* **1989,** 24, (3), 284-9.
4. Vanepps, D. E.; Strickland, R. G.; Williams, R. C., Inhibitors of Leukocyte Chemotaxis in Alcoholic Liver-Disease. *American Journal of Medicine* **1975,** 59, (2), 200-207.
5. Campbell, A. C.; Dronfield, M. W.; Toghill, P. J.; Reeves, W. G., Neutrophil function in chronic liver disease. *Clin Exp Immunol* **1981,** 45, (1), 81-9.
6. Fiuza, C.; Salcedo, M.; Clemente, G.; Tellado, J. M., Granulocyte colony-stimulating factor improves deficient in vitro neutrophil transendothelial migration in patients with advanced liver disease. *Clinical and Diagnostic Laboratory Immunology* **2002,** 9, (2), 433-439.
7. Artru, F.; Bou Saleh, M.; Maggiotto, F.; Lassailly, G.; Ningarhari, M.; Demaret, J.; Ntandja-Wandji, L. C.; Pais de Barros, J. P.; Labreuche, J.; Drumez, E.; Helou, D. G.; Dharancy, S.; Gantier, E.; Perianin, A.; Chollet-Martin, S.; Bataller, R.; Mathurin, P.; Dubuquoy, L.; Louvet, A., IL-33/ST2 pathway regulates neutrophil migration and predicts outcome in patients with severe alcoholic hepatitis. *J Hepatol* **2020,** 72, (6), 1052-1061.
8. Claria, J.; Titos, E.; Jimenez, W.; Ros, J.; Gines, P.; Arroyo, V.; Rivera, F.; Rodes, J., Altered biosynthesis of leukotrienes and lipoxins and host defense disorders in patients with cirrhosis and ascites. *Gastroenterology* **1998,** 115, (1), 147-56.
9. Langer, M. M.; Sichelschmidt, S.; Bauschen, A.; Bornemann, L.; Guckenbiehl, S.; Gunzer, M.; Lange, C. M., Pathological neutrophil migration predicts adverse outcomes in hospitalized patients with liver cirrhosis. *Liver Int* **2023,** 43, (4), 896-905.
10. Laffi, G.; Carloni, V.; Baldi, E.; Rossi, M. E.; Azzari, C.; Gresele, P.; Marra, F.; Gentilini, P., Impaired superoxide anion, platelet-activating factor, and leukotriene B4 synthesis by neutrophils in cirrhosis. *Gastroenterology* **1993,** 105, (1), 170-7.
11. Knooihuizen, S. A. I.; Alexander, N. J.; Hopke, A.; Barros, N.; Viens, A.; Scherer, A.; Atallah, N. J.; Dagher, Z.; Irimia, D.; Chung, R. T.; Mansour, M. K., Loss of Coordinated Neutrophil Responses to the Human Fungal Pathogen, Candida albicans, in Patients With Cirrhosis. *Hepatol Commun* **2021,** 5, (3), 502-515.
12. De Fernandez, M. A.; Clark, A.; Triger, D. R., Neutrophil phagocytic and bactericidal function in primary biliary cirrhosis and other chronic liver diseases. *Clin Exp Immunol* **1987,** 67, (3), 655-61.
13. Rajkovic, I. A.; Williams, R., Abnormalities of neutrophil phagocytosis, intracellular killing and metabolic activity in alcoholic cirrhosis and hepatitis. *Hepatology* **1986,** 6, (2), 252-62.
14. Mookerjee, R. P.; Stadlbauer, V.; Lidder, S.; Wright, G. A.; Hodges, S. J.; Davies, N. A.; Jalan, R., Neutrophil dysfunction in alcoholic hepatitis superimposed on cirrhosis is reversible and predicts the outcome. *Hepatology* **2007,** 46, (3), 831-40.
15. Stadlbauer, V.; Mookerjee, R. P.; Hodges, S.; Wright, G. A.; Davies, N. A.; Jalan, R., Effect of probiotic treatment on deranged neutrophil function and cytokine responses in patients with compensated alcoholic cirrhosis. *J Hepatol* **2008,** 48, (6), 945-51.
16. Huang, C. H.; Jeng, W. J.; Ho, Y. P.; Teng, W.; Hsieh, Y. C.; Chen, W. T.; Chen, Y. C.; Lin, H. H.; Sheen, I. S.; Lin, C. Y., Increased EMR2 expression on neutrophils correlates with disease severity and predicts overall mortality in cirrhotic patients. *Sci Rep* **2016,** 6, 38250.
17. Sehgal, R.; Kaur, N.; Maiwall, R.; Ramakrishna, G.; Maras, J. S.; Trehanpati, N., Plasma Proteomic Analysis Identified Proteins Associated with Faulty Neutrophils Functionality in Decompensated Cirrhosis Patients with Sepsis. *Cells* **2022,** 11, (11).
18. Horvath, A.; Leber, B.; Schmerboeck, B.; Tawdrous, M.; Zettel, G.; Hartl, A.; Madl, T.; Stryeck, S.; Fuchs, D.; Lemesch, S.; Douschan, P.; Krones, E.; Spindelboeck, W.; Durchschein, F.; Rainer, F.; Zollner, G.; Stauber, R. E.; Fickert, P.; Stiegler, P.; Stadlbauer, V., Randomised clinical trial: the effects of a multispecies probiotic vs. placebo on innate immune function,

bacterial translocation and gut permeability in patients with cirrhosis. *Aliment Pharmacol Ther* **2016,** 44, (9), 926-935.
19. Taylor, N. J.; Vijay, G. K. M.; Abeles, R. D.; Auzinger, G.; Bernal, W.; Ma, Y.; Wendon, J. A.; Shawcross, D. L., The severity of circulating neutrophil dysfunction in patients with cirrhosis is associated with 90-day and 1-year mortality. *Alimentary Pharmacology & Therapeutics* **2014,** 40, (6), 705-715.
20. Wu, W.; Sun, S.; Wang, Y.; Zhao, R.; Ren, H.; Li, Z.; Zhao, H.; Zhang, Y.; Sheng, J.; Chen, Z.; Shi, Y., Circulating Neutrophil Dysfunction in HBV-Related Acute-on-Chronic Liver Failure. *Front Immunol* **2021,** 12, 620365.
21. Balazs, I.; Horvath, A.; Leber, B.; Feldbacher, N.; Sattler, W.; Rainer, F.; Fauler, G.; Vermeren, S.; Stadlbauer, V., Serum bile acids in liver cirrhosis promote neutrophil dysfunction. *Clin Transl Med* **2022,** 12, (2), e735.
22. Leber, B.; Balazs, I.; Horvath, A.; Posch, A.; Streit, A.; Spindelbock, W.; Feldbacher, N.; Stiegler, P.; Stauber, R. E.; Rechberger, G. N.; Kollroser, M.; Sattler, W.; Nusshold, C.; Stadlbauer, V., Direct acting antiviral therapy rescues neutrophil dysfunction and reduces hemolysis in hepatitis C infection. *Transl Res* **2021,** 232, 103-114.
23. Makkar, K.; Tomer, S.; Verma, N.; Rathi, S.; Arora, S. K.; Taneja, S.; Duseja, A.; Chawla, Y. K.; Dhiman, R. K., Neutrophil dysfunction predicts 90-day survival in patients with acute on chronic liver failure: A longitudinal case-control study. *JGH Open* **2020,** 4, (4), 595-602.
24. Tritto, G.; Bechlis, Z.; Stadlbauer, V.; Davies, N.; Frances, R.; Shah, N.; Mookerjee, R. P.; Such, J.; Jalan, R., Evidence of neutrophil functional defect despite inflammation in stable cirrhosis. *J Hepatol* **2011,** 55, (3), 574-581.
25. Boussif, A.; Rolas, L.; Weiss, E.; Bouriche, H.; Moreau, R.; Perianin, A., Impaired intracellular signaling, myeloperoxidase release and bactericidal activity of neutrophils from patients with alcoholic cirrhosis. *Journal of Hepatology* **2016,** 64, (5), 1041-1048.
26. Bruns, T.; Peter, J.; Hagel, S.; Herrmann, A.; Stallmach, A., The augmented neutrophil respiratory burst in response to Escherichia coli is reduced in liver cirrhosis during infection. *Clin Exp Immunol* **2011,** 164, (3), 346-56.
27. Rolas, L.; Makhezer, N.; Hadjoudj, S.; El-Benna, J.; Djerdjouri, B.; Elkrief, L.; Moreau, R.; Perianin, A., Inhibition of mammalian target of rapamycin aggravates the respiratory burst defect of neutrophils from decompensated patients with cirrhosis. *Hepatology* **2013,** 57, (3), 1163-71.
28. Garfia, C.; Garcia-Ruiz, I.; Solis-Herruzo, J. A., Deficient phospholipase C activity in blood polimorphonuclear neutrophils from patients with liver cirrhosis. *Journal of Hepatology* **2004,** 40, (5), 749-756.
29. Rolas, L.; Boussif, A.; Weiss, E.; Letteron, P.; Haddad, O.; El-Benna, J.; Rautou, P. E.; Moreau, R.; Perianin, A., NADPH oxidase depletion in neutrophils from patients with cirrhosis and restoration via toll-like receptor 7/8 activation. *Gut* **2018,** 67, (8), 1505-1516.
30. Weiss, E.; de la Grange, P.; Defaye, M.; Lozano, J. J.; Aguilar, F.; Hegde, P.; Jolly, A.; Moga, L.; Sukriti, S.; Agarwal, B.; Gurm, H.; Tanguy, M.; Poisson, J.; Claria, J.; Abback, P. S.; Perianin, A.; Mehta, G.; Jalan, R.; Francoz, C.; Rautou, P. E.; Lotersztajn, S.; Arroyo, V.; Durand, F.; Moreau, R., Characterization of Blood Immune Cells in Patients With Decompensated Cirrhosis Including ACLF. *Front Immunol* **2020,** 11, 619039.
31. Masini, E.; Mugnai, L.; Foschi, M.; Laffi, G.; Gentilini, P.; Mannaioni, P. F., Changes in the production of nitric oxide and superoxide by inflammatory cells in liver cirrhosis. *Int Arch Allergy Immunol* **1995,** 107, (1-3), 197-8.
32. Tranah, T. H.; Vijay, G. K. M.; Ryan, J. M.; Abeles, R. D.; Middleton, P. K.; Shawcross, D. L., Dysfunctional neutrophil effector organelle mobilization and microbicidal protein release in alcohol-related cirrhosis. *Am J Physiol Gastrointest Liver Physiol* **2017,** 313, (3), G203-G211.
33. Agraz-Cibrian, J. M.; Segura-Ortega, J. E.; Delgado-Rizo, V.; Fafutis-Morris, M., Alterations in neutrophil extracellular traps is associated with the degree of decompensation of liver cirrhosis. *J Infect Dev Ctries* **2016,** 10, (5), 512-7.
34. Agraz-Cibrian, J. M.; Delgado-Rizo, V.; Segura-Ortega, J. E.; Maldonado-Gomez, H. A.; Zambrano-Zaragoza, J. F.; Duran-Avelar, M. J.; Vibanco-Perez, N.; Fafutis-Morris, M., Impaired neutrophil extracellular traps and inflammatory responses in the peritoneal fluid of patients with liver cirrhosis. *Scand J Immunol* **2018,** 88, (5), e12714.

35. Zenlander, R.; Havervall, S.; Magnusson, M.; Engstrand, J.; Agren, A.; Thalin, C.; Stal, P., Neutrophil extracellular traps in patients with liver cirrhosis and hepatocellular carcinoma. *Sci Rep* **2021,** 11, (1), 18025.
36. Blasi, A.; Patel, V. C.; Adelmeijer, J.; Azarian, S.; Aziz, F.; Fernandez, J.; Bernal, W.; Lisman, T., Plasma levels of circulating DNA are associated with outcome, but not with activation of coagulation in decompensated cirrhosis and ACLF. *JHEP Rep* **2019,** 1, (3), 179-187.
37. Kusaba, N.; Kumashiro, R.; Ogata, H.; Sata, M.; Tanikawa, K., In vitro study of neutrophil apoptosis in liver cirrhosis. *Intern Med* **1998,** 37, (1), 11-7.
38. Ramirez, M. J.; Titos, E.; Claria, J.; Navasa, M.; Fernandez, J.; Rodes, J., Increased apoptosis dependent on caspase-3 activity in polymorphonuclear leukocytes from patients with cirrhosis and ascites. *J Hepatol* **2004,** 41, (1), 44-8.
